# Supplementary figures and images for: Steps to Facilitate the Use of Clinical Gait Analysis in Stroke Patients: The Validation of a Single 2D RGB Smartphone Video-Based System for Gait Analysis (part 2 of 2)
Source: Sensors (Basel). 2024 Dec 6;24(23):7819. doi: 10.3390/s24237819 (PMC11644854; doi:10.3390/s24237819)

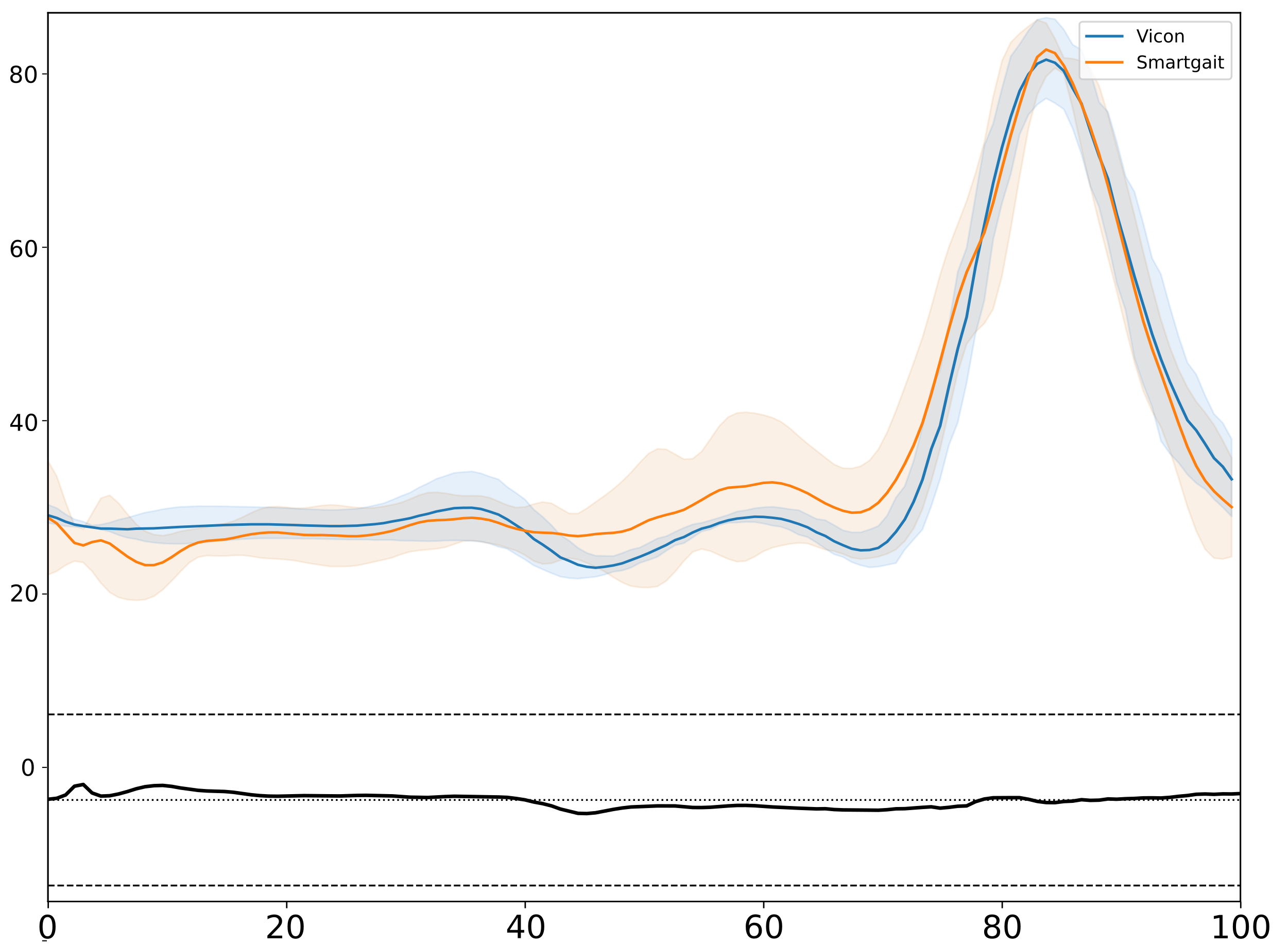

Supplement: Supplementary file 1 [file sensors-24-07819-s001.zip › spm_eval_SE05CH16_sagital/SE05CH16_angle_(2, 5, 5, 8)2.csv_plot_spm_fixed.png]

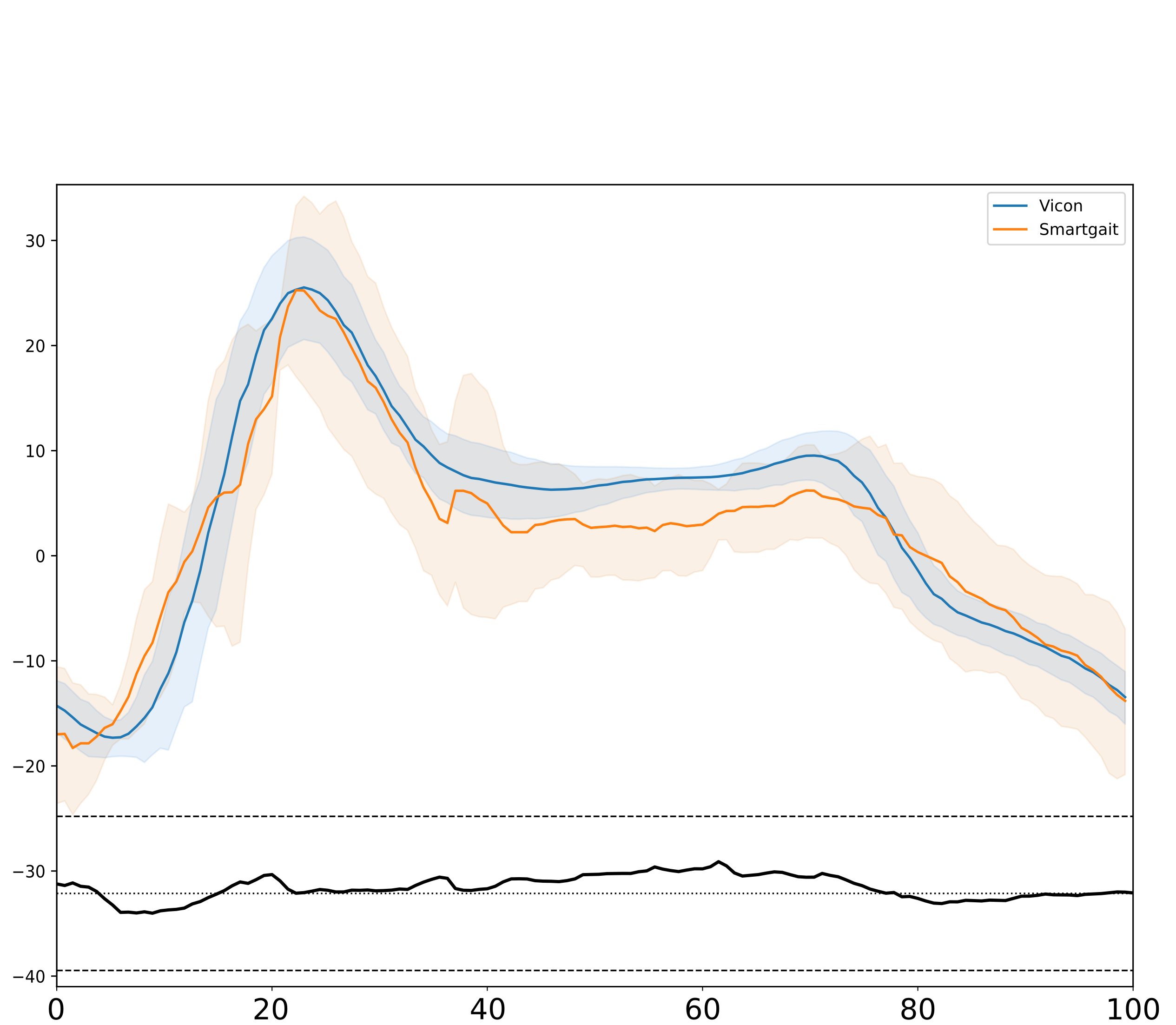

Supplement: Supplementary file 1 [file sensors-24-07819-s001.zip › spm_eval_SE05CH16_sagital/SE05CH16_angle_(2, 5, 12, 0)3.csv_plot_spm_fixed.png]

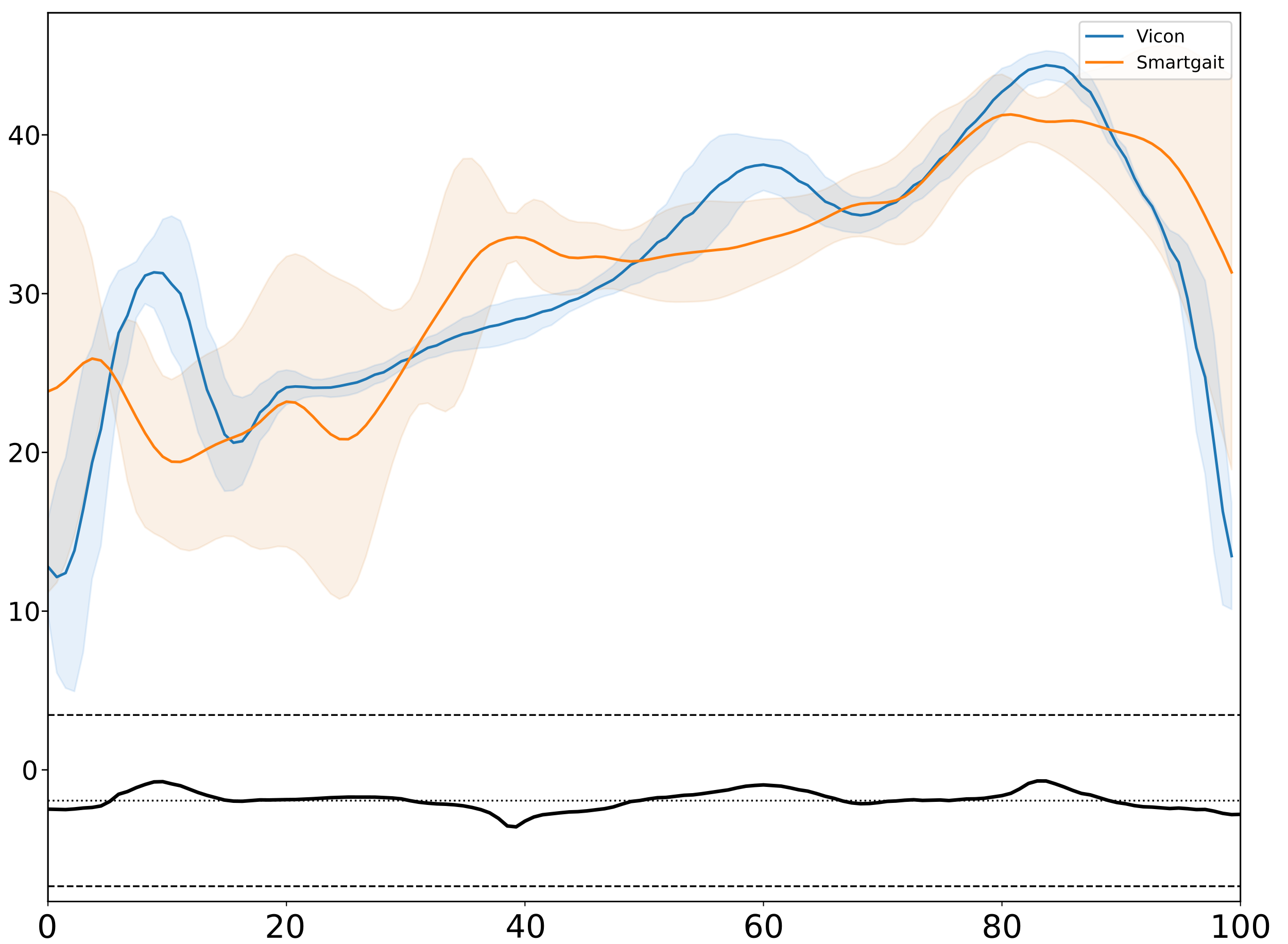

Supplement: Supplementary file 1 [file sensors-24-07819-s001.zip › spm_eval_SE05CH16_sagital/SE05CH16_angle_(5, 8, 8, 11)2.csv_plot_spm_fixed.png]

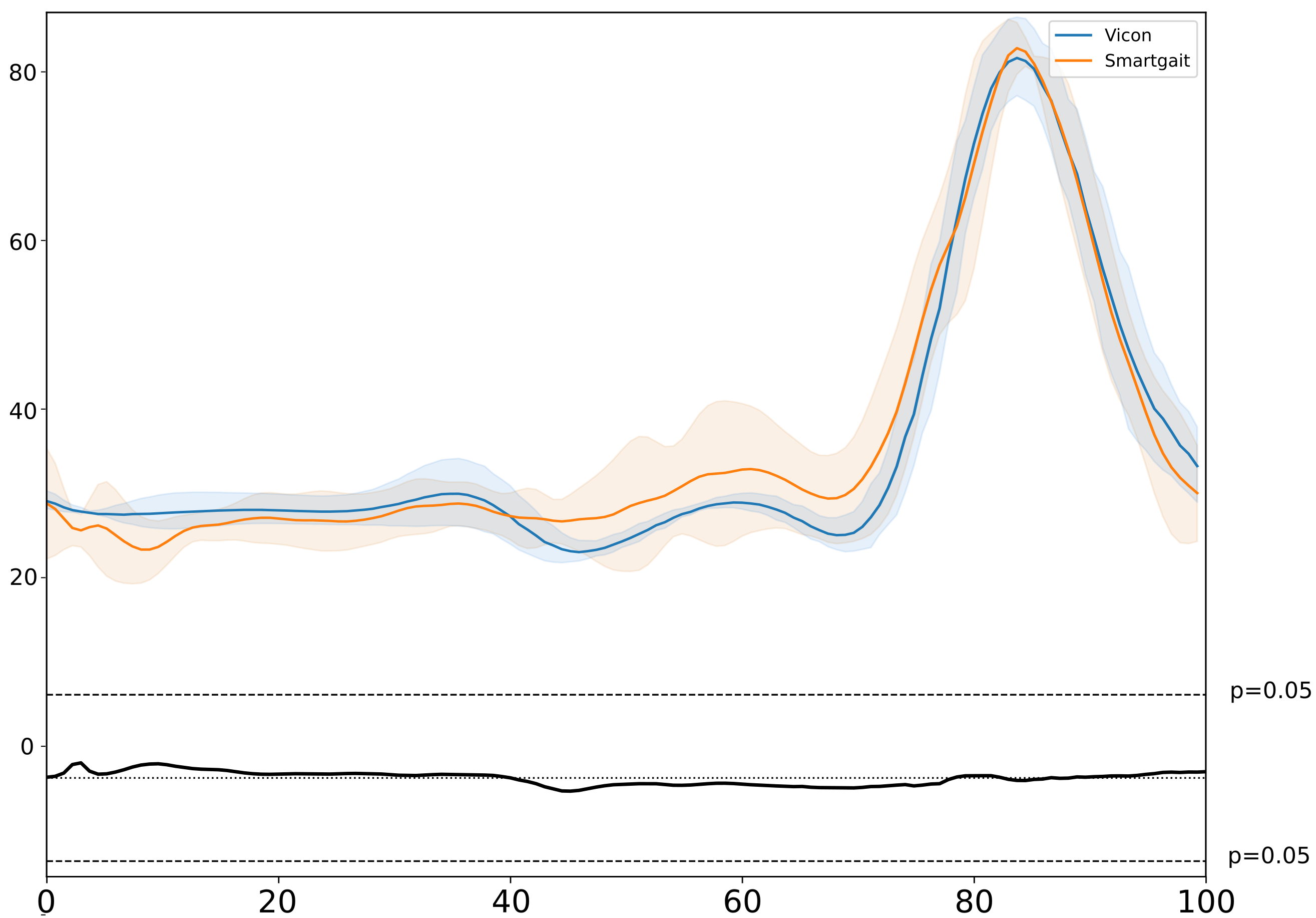

Supplement: Supplementary file 1 [file sensors-24-07819-s001.zip › spm_eval_SE05CH16_sagital/SE05CH16_angle_(2, 5, 5, 8)2.csv_plot_spm_fixed_.png]

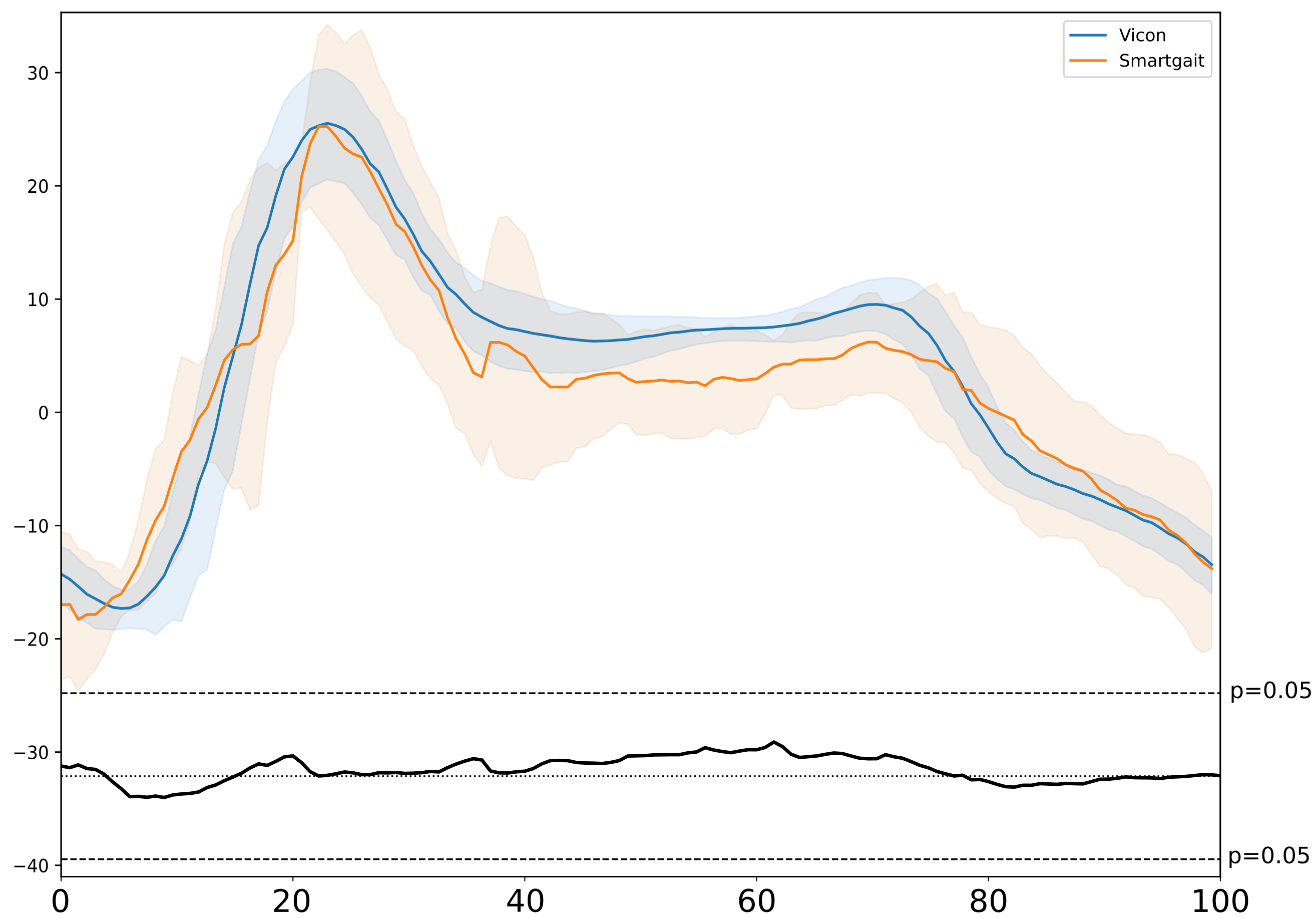

Supplement: Supplementary file 1 [file sensors-24-07819-s001.zip › spm_eval_SE05CH16_sagital/SE05CH16_angle_(2, 5, 12, 0)3.csv_plot_spm_fixed_.png]

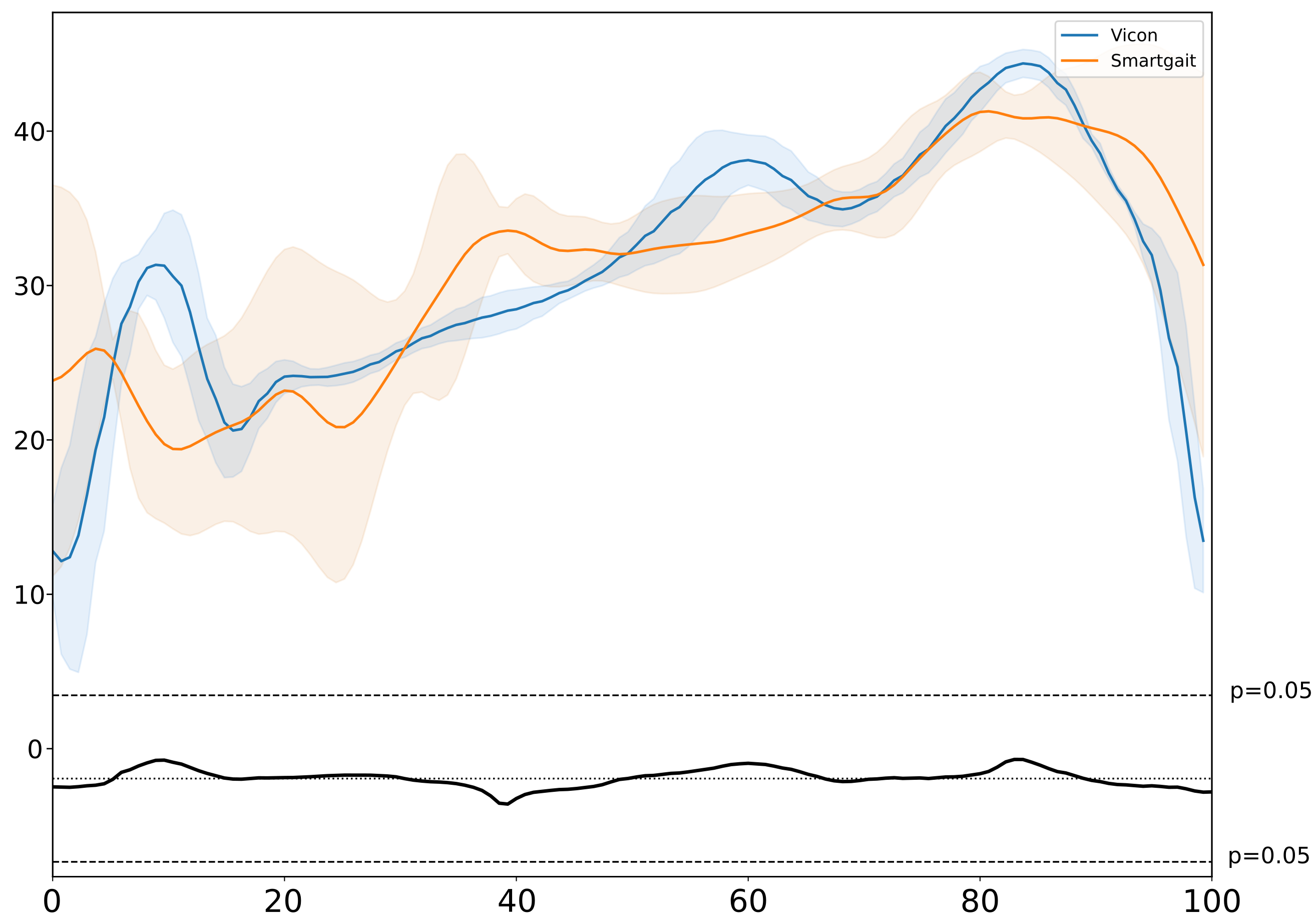

Supplement: Supplementary file 1 [file sensors-24-07819-s001.zip › spm_eval_SE05CH16_sagital/SE05CH16_angle_(5, 8, 8, 11)2.csv_plot_spm_fixed_.png]

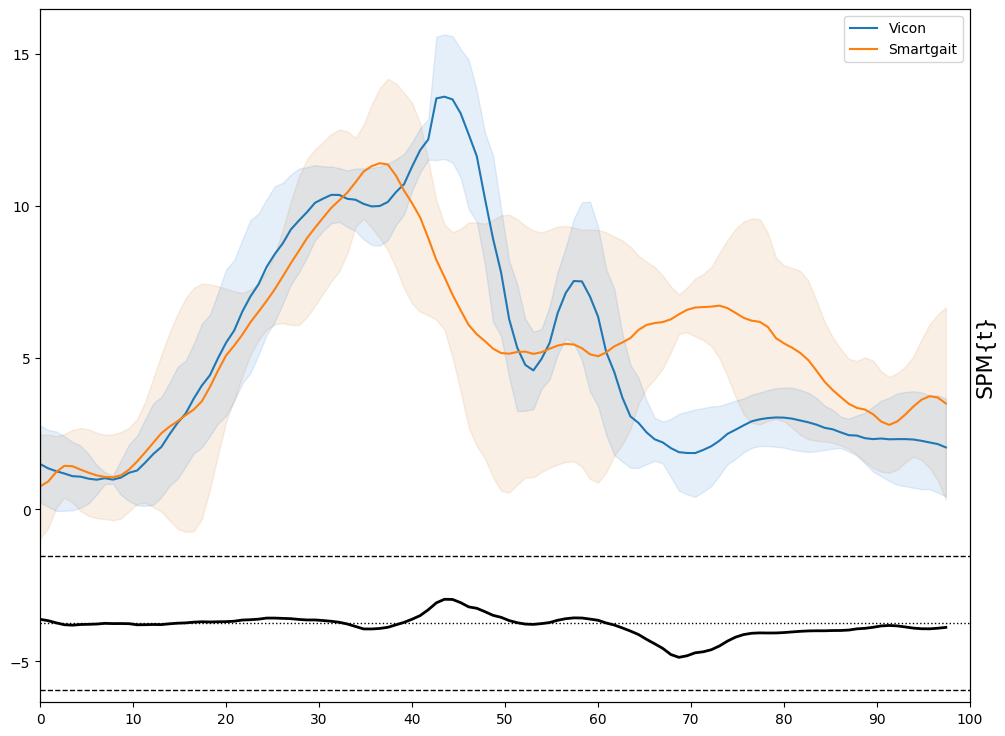

Supplement: Supplementary file 1 [file sensors-24-07819-s001.zip › spm_eval_SI18SC18_frontal/SI18SC18_angle_(2, 5, 12, 0)0.csv_plot_spm.png]

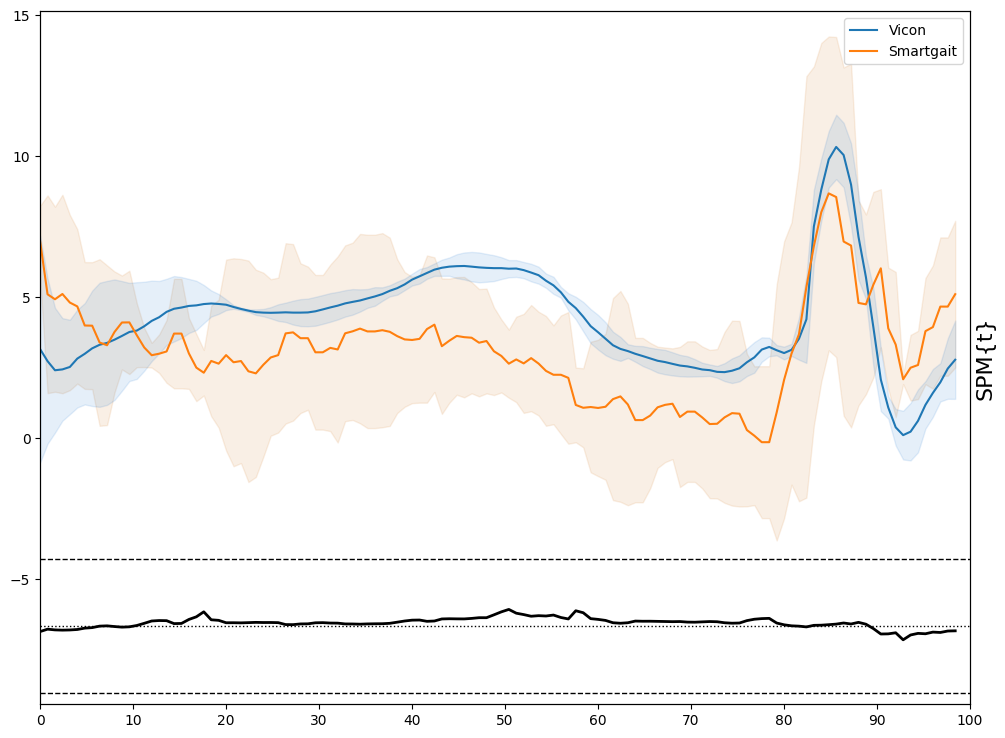

Supplement: Supplementary file 1 [file sensors-24-07819-s001.zip › spm_eval_SI18SC18_frontal/SI18SC18_angle_(2, 5, 5, 8)2.csv_plot_spm.png]

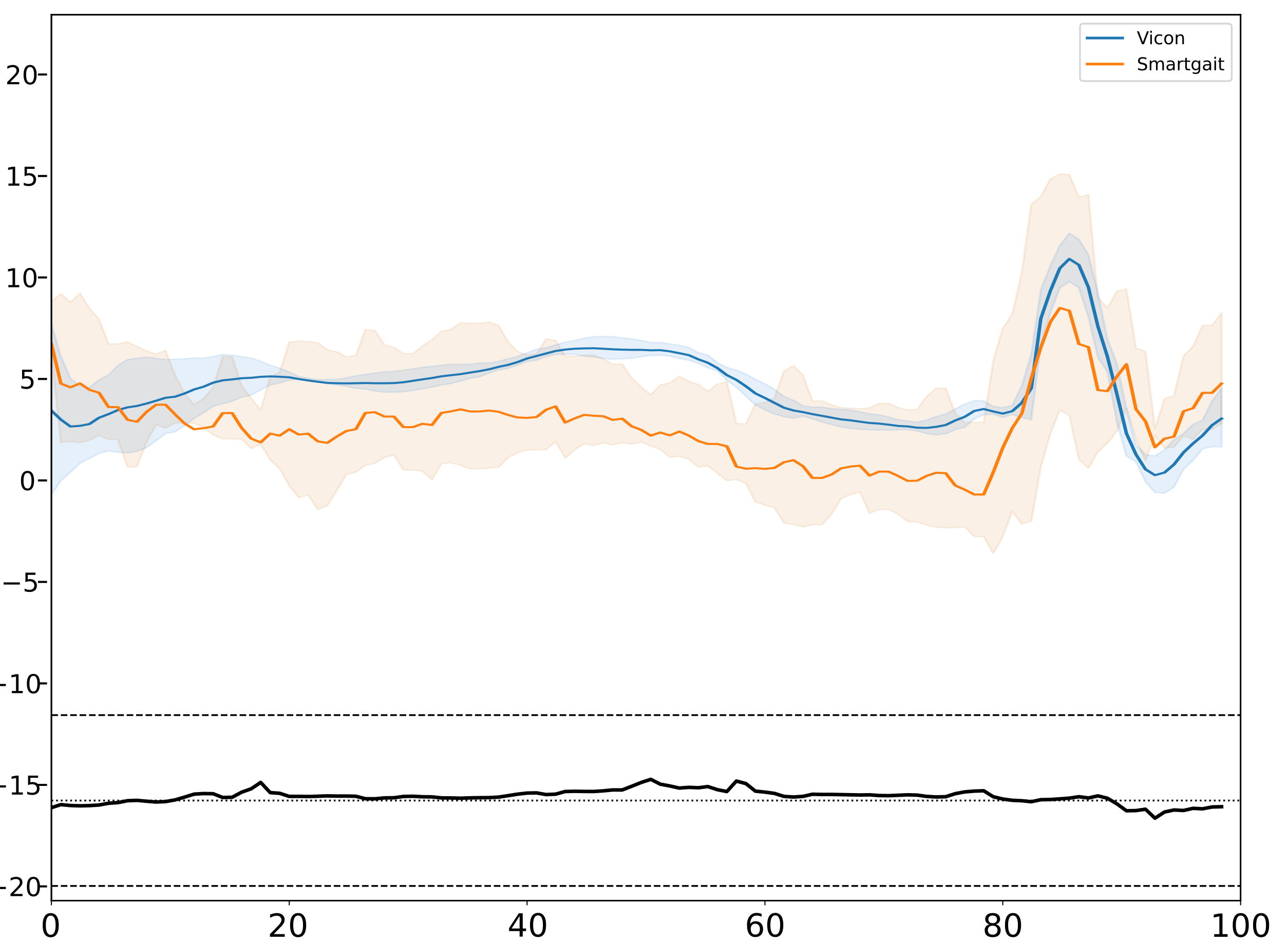

Supplement: Supplementary file 1 [file sensors-24-07819-s001.zip › spm_eval_SI18SC18_frontal/SI18SC18_angle_(2, 5, 5, 8)2.csv_plot_spm_fixed.png]

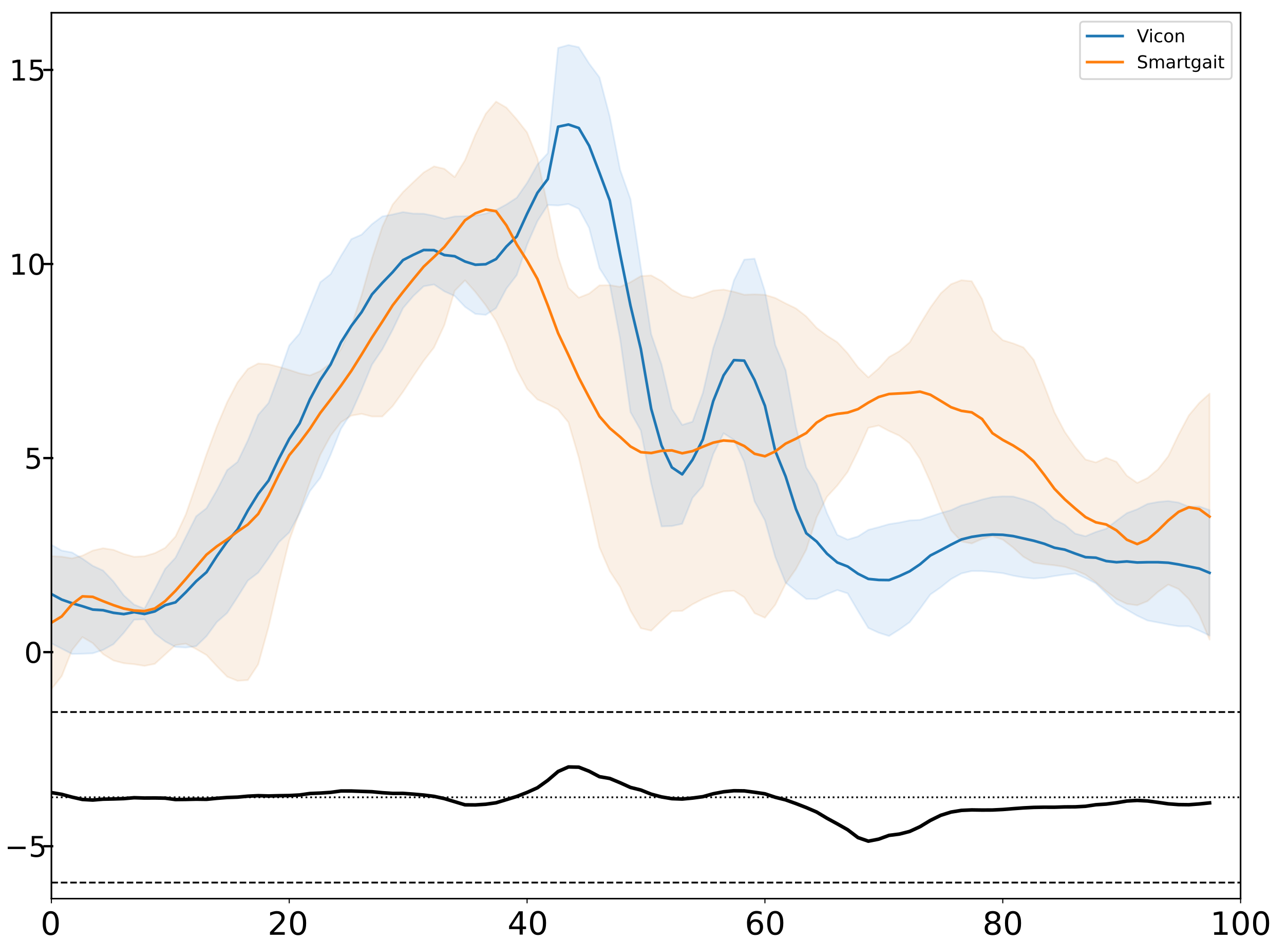

Supplement: Supplementary file 1 [file sensors-24-07819-s001.zip › spm_eval_SI18SC18_frontal/SI18SC18_angle_(2, 5, 12, 0)0.csv_plot_spm_fixed.png]

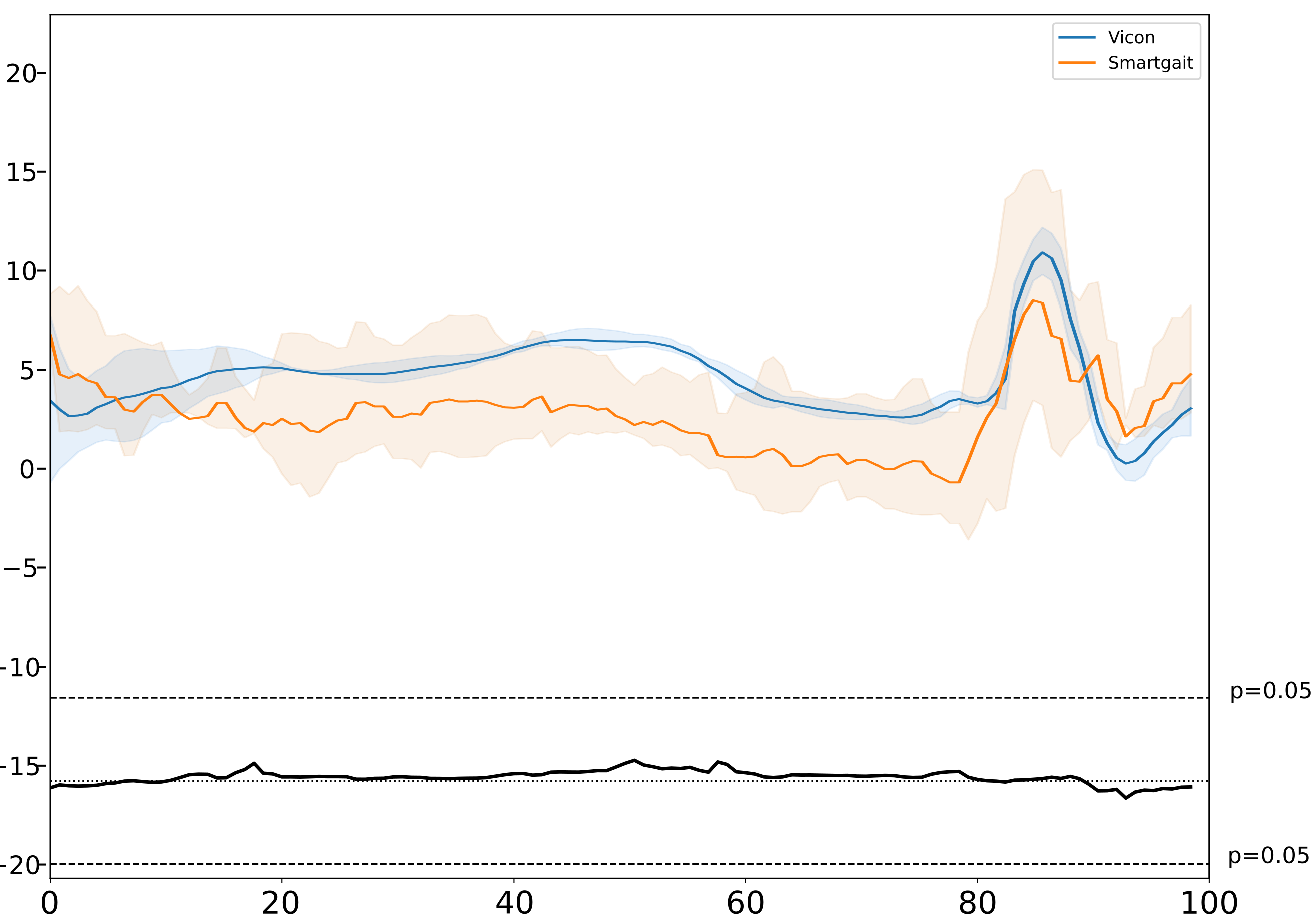

Supplement: Supplementary file 1 [file sensors-24-07819-s001.zip › spm_eval_SI18SC18_frontal/SI18SC18_angle_(2, 5, 5, 8)2.csv_plot_spm_fixed_.png]

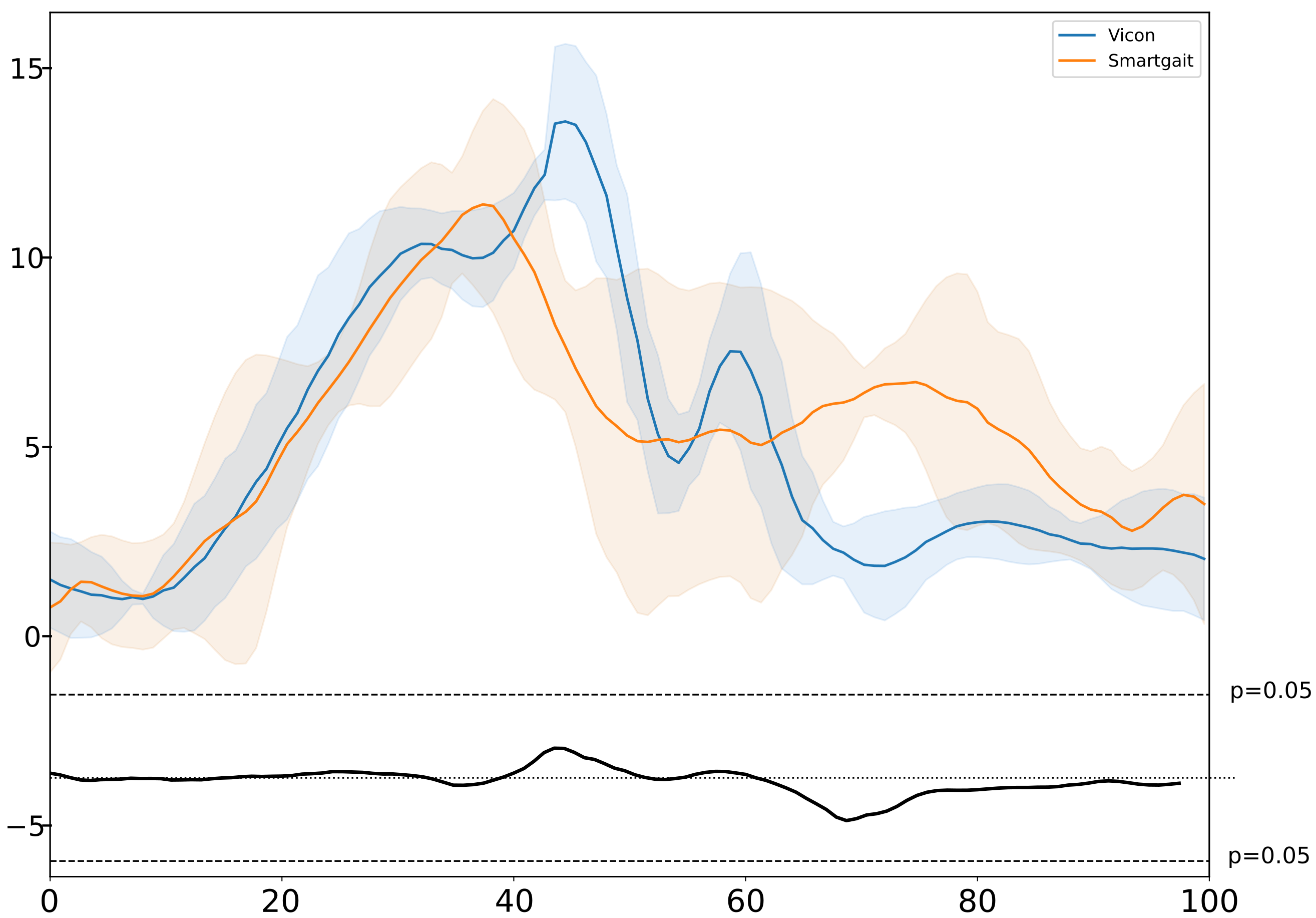

Supplement: Supplementary file 1 [file sensors-24-07819-s001.zip › spm_eval_SI18SC18_frontal/SI18SC18_angle_(2, 5, 12, 0)0.csv_plot_spm_fixed_.png]

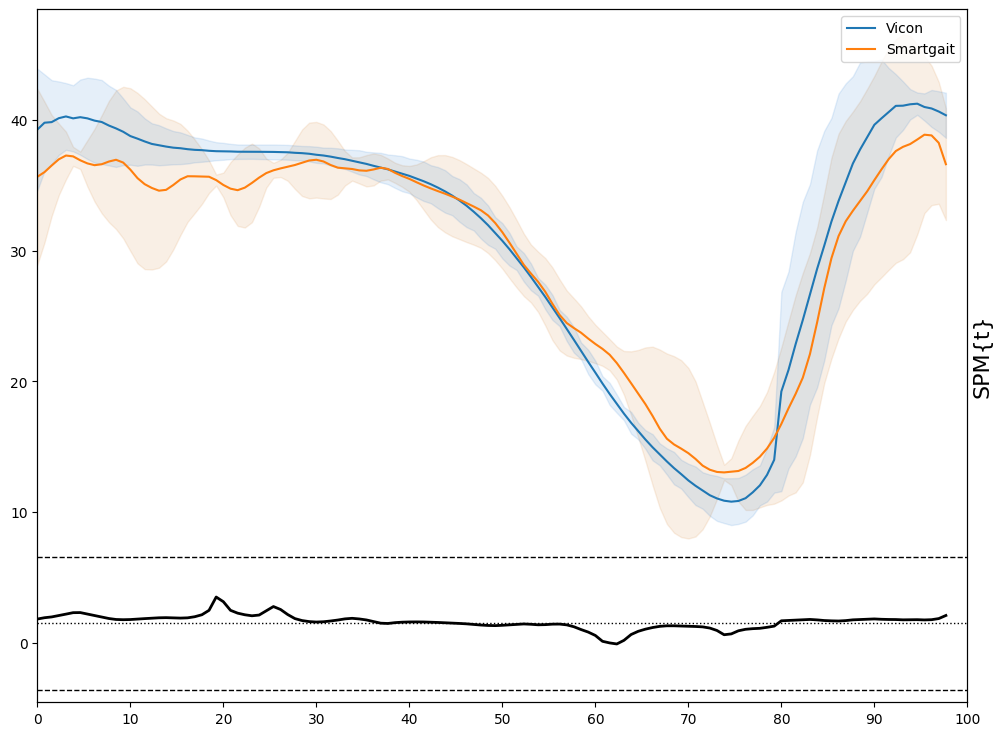

Supplement: Supplementary file 1 [file sensors-24-07819-s001.zip › spm_eval_SI18SC18_sagital/SI18SC18_angle_(2, 5, 12, 0)2.csv_plot_spm.png]

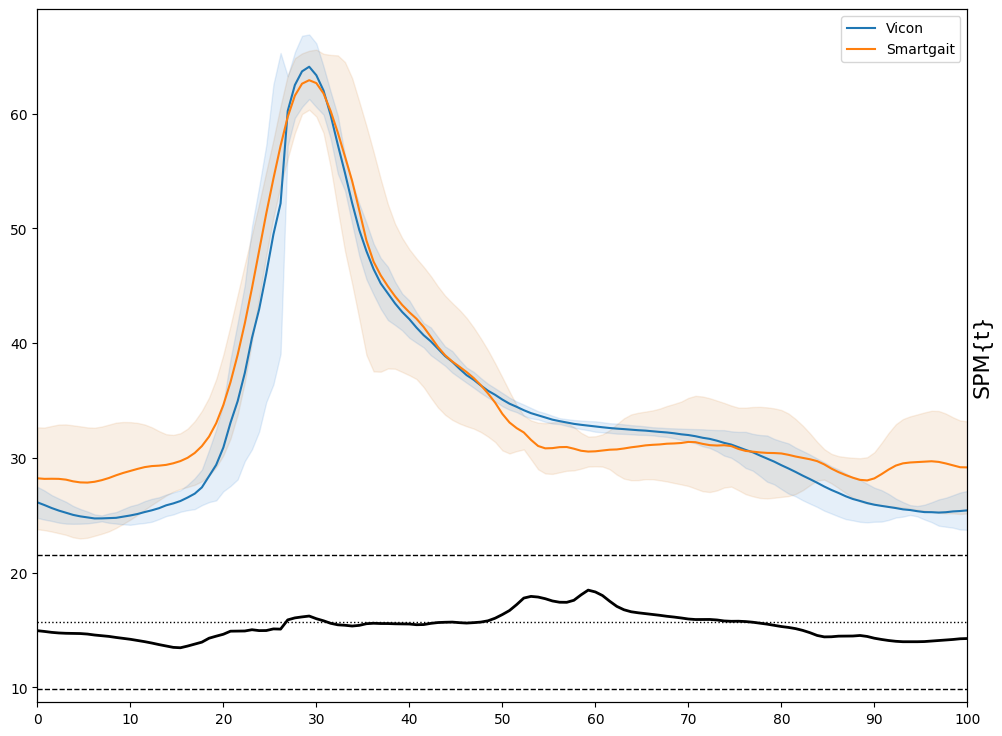

Supplement: Supplementary file 1 [file sensors-24-07819-s001.zip › spm_eval_SI18SC18_sagital/SI18SC18_angle_(2, 5, 5, 8)3.csv_plot_spm.png]

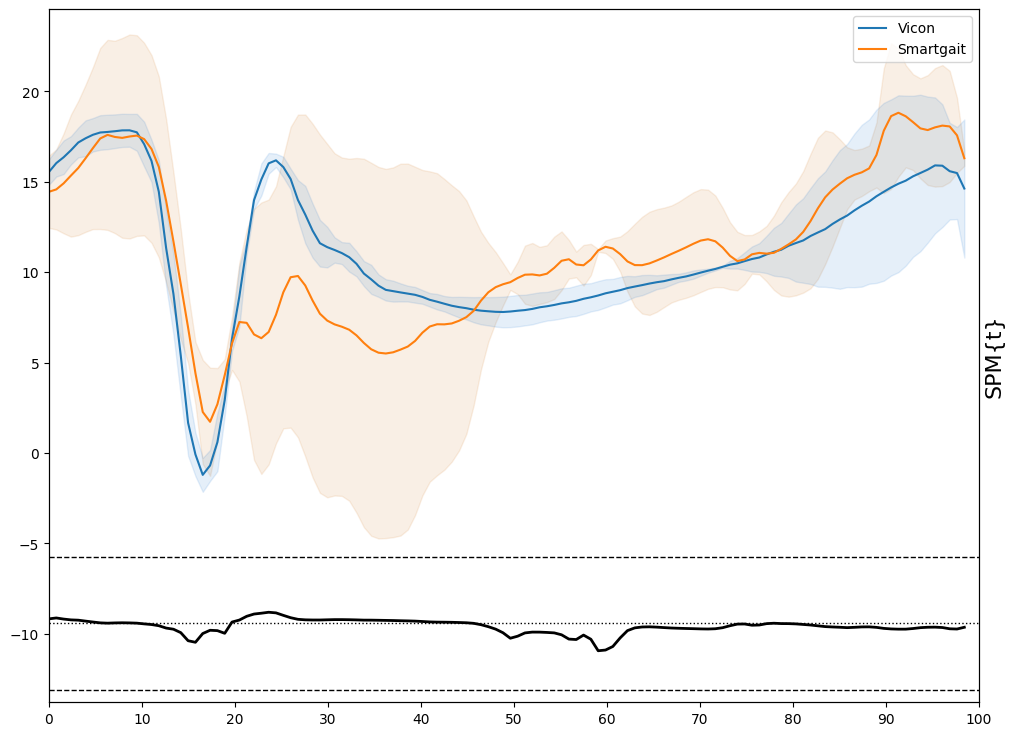

Supplement: Supplementary file 1 [file sensors-24-07819-s001.zip › spm_eval_SI18SC18_sagital/SI18SC18_angle_(5, 8, 8, 11)3.csv_plot_spm.png]

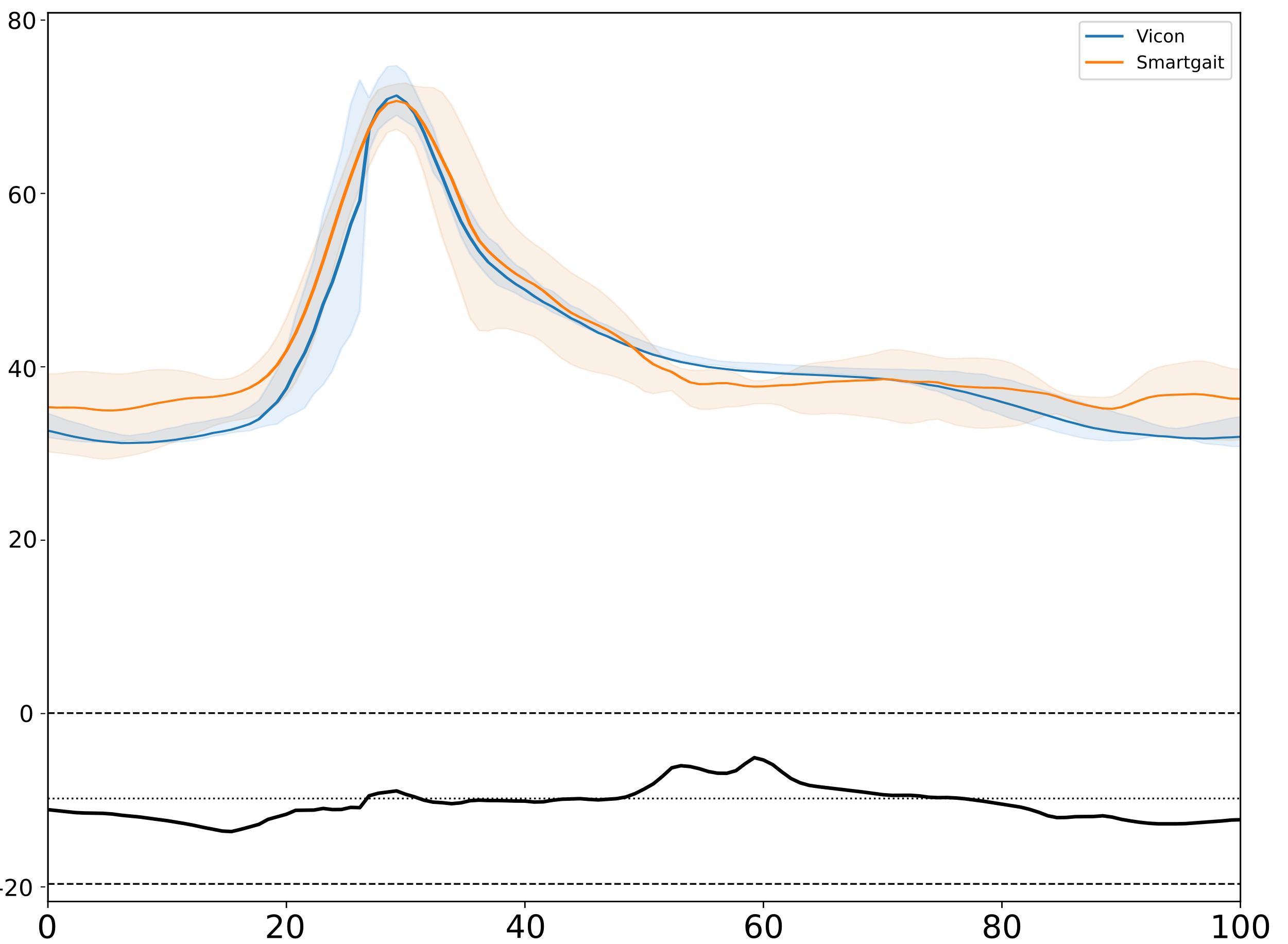

Supplement: Supplementary file 1 [file sensors-24-07819-s001.zip › spm_eval_SI18SC18_sagital/SI18SC18_angle_(2, 5, 5, 8)3.csv_plot_spm_fixed.png]

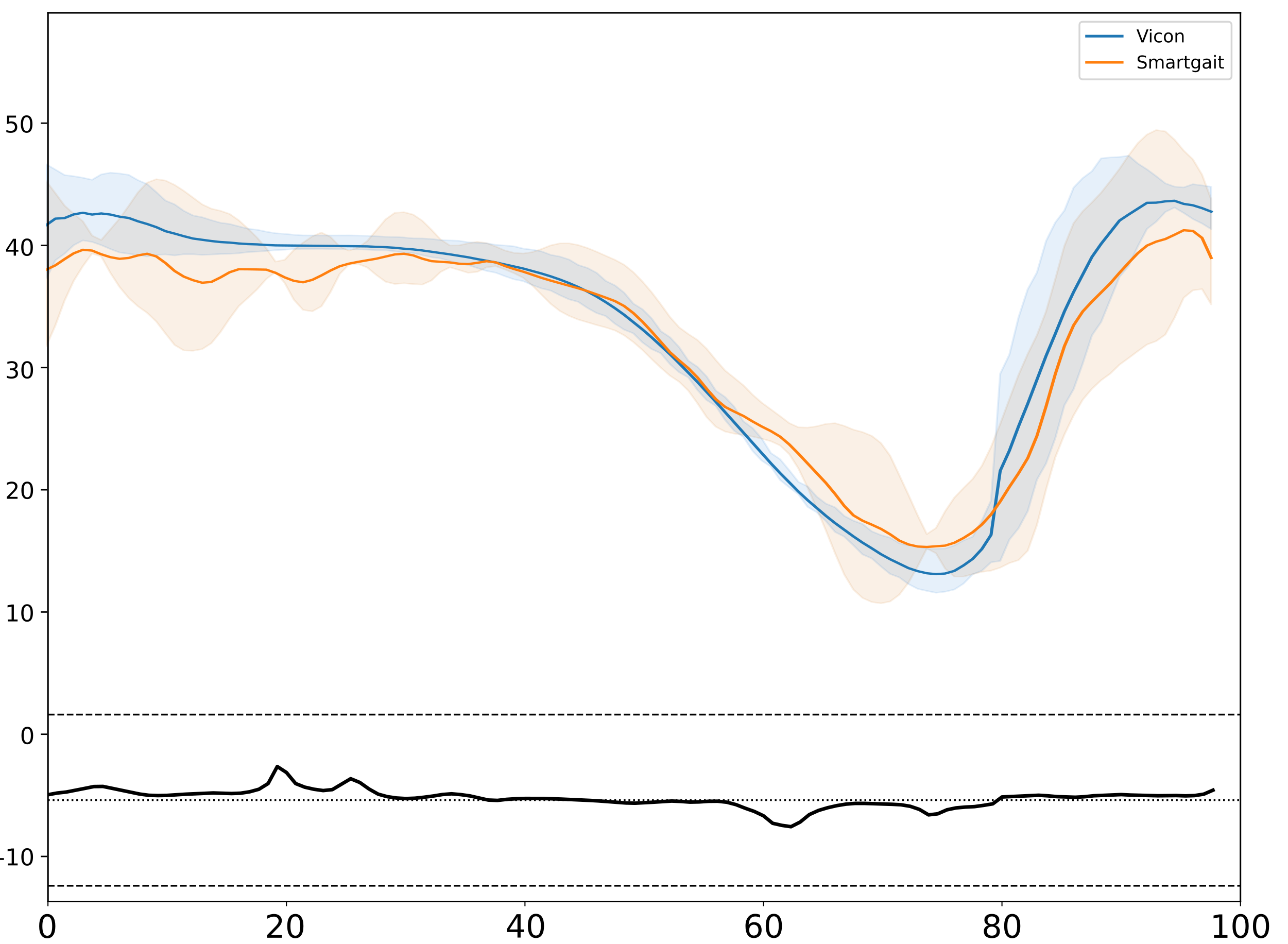

Supplement: Supplementary file 1 [file sensors-24-07819-s001.zip › spm_eval_SI18SC18_sagital/SI18SC18_angle_(2, 5, 12, 0)2.csv_plot_spm_fixed.png]

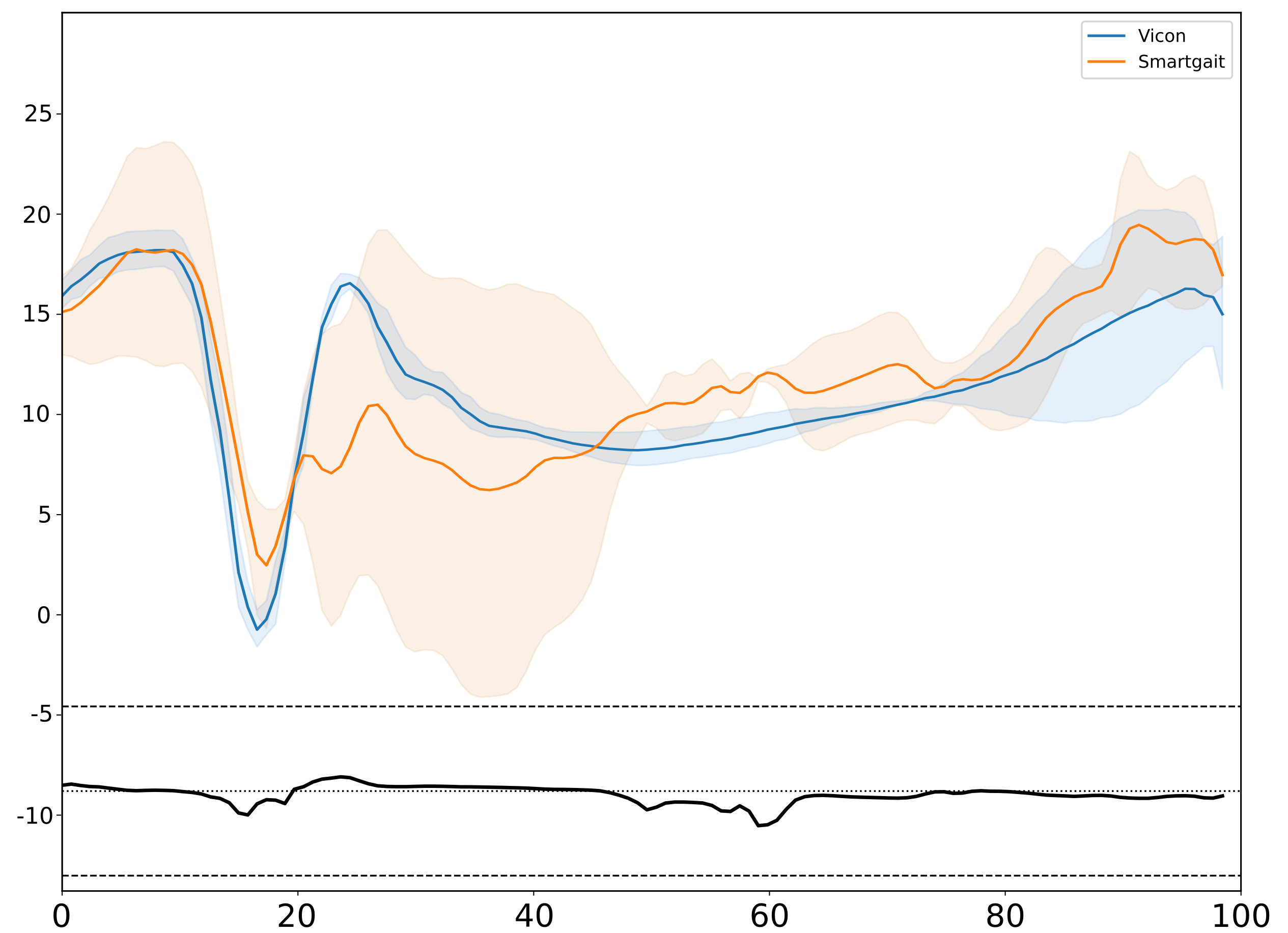

Supplement: Supplementary file 1 [file sensors-24-07819-s001.zip › spm_eval_SI18SC18_sagital/SI18SC18_angle_(5, 8, 8, 11)3.csv_plot_spm_fixed.png]

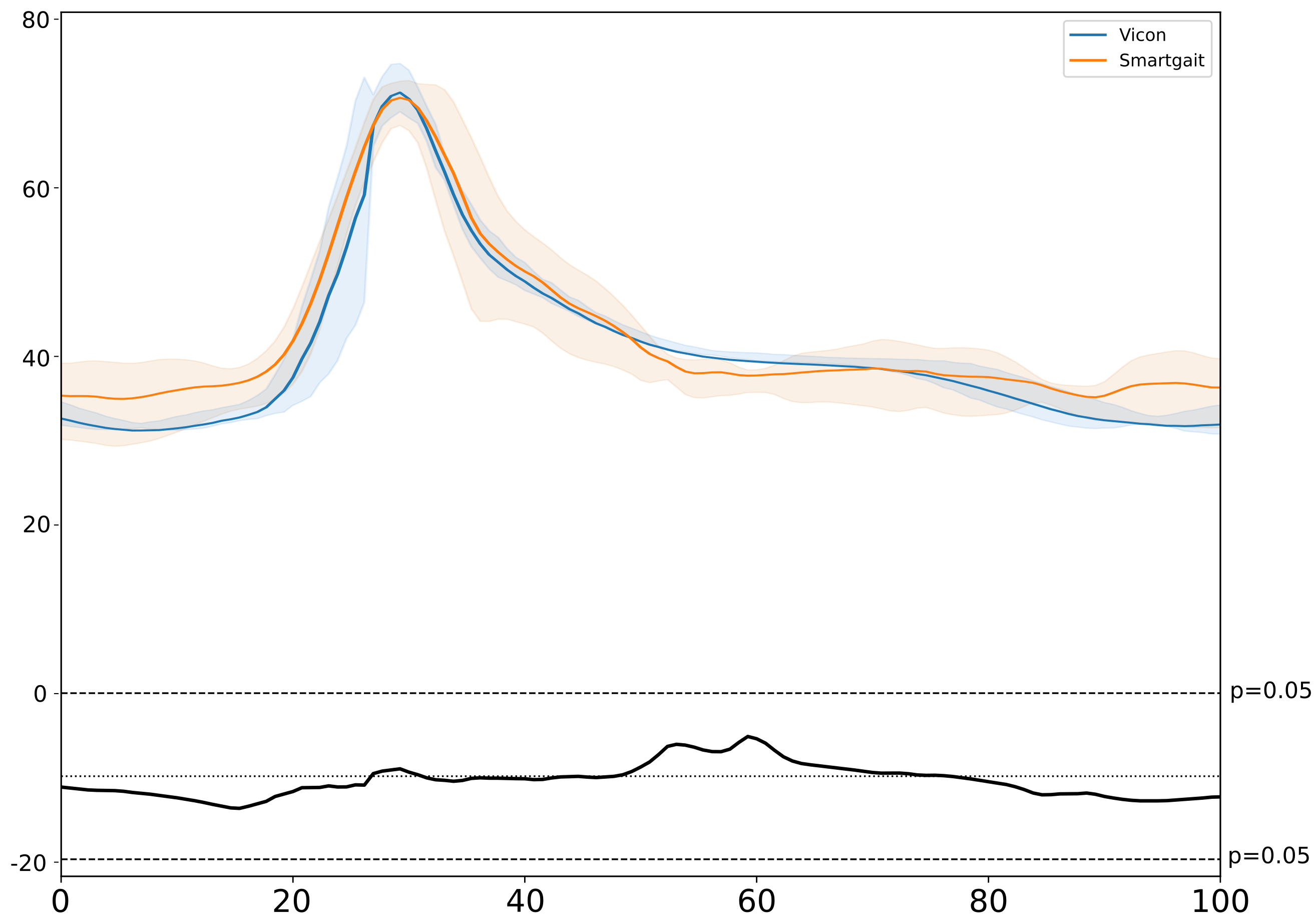

Supplement: Supplementary file 1 [file sensors-24-07819-s001.zip › spm_eval_SI18SC18_sagital/SI18SC18_angle_(2, 5, 5, 8)3.csv_plot_spm_fixed_.png]

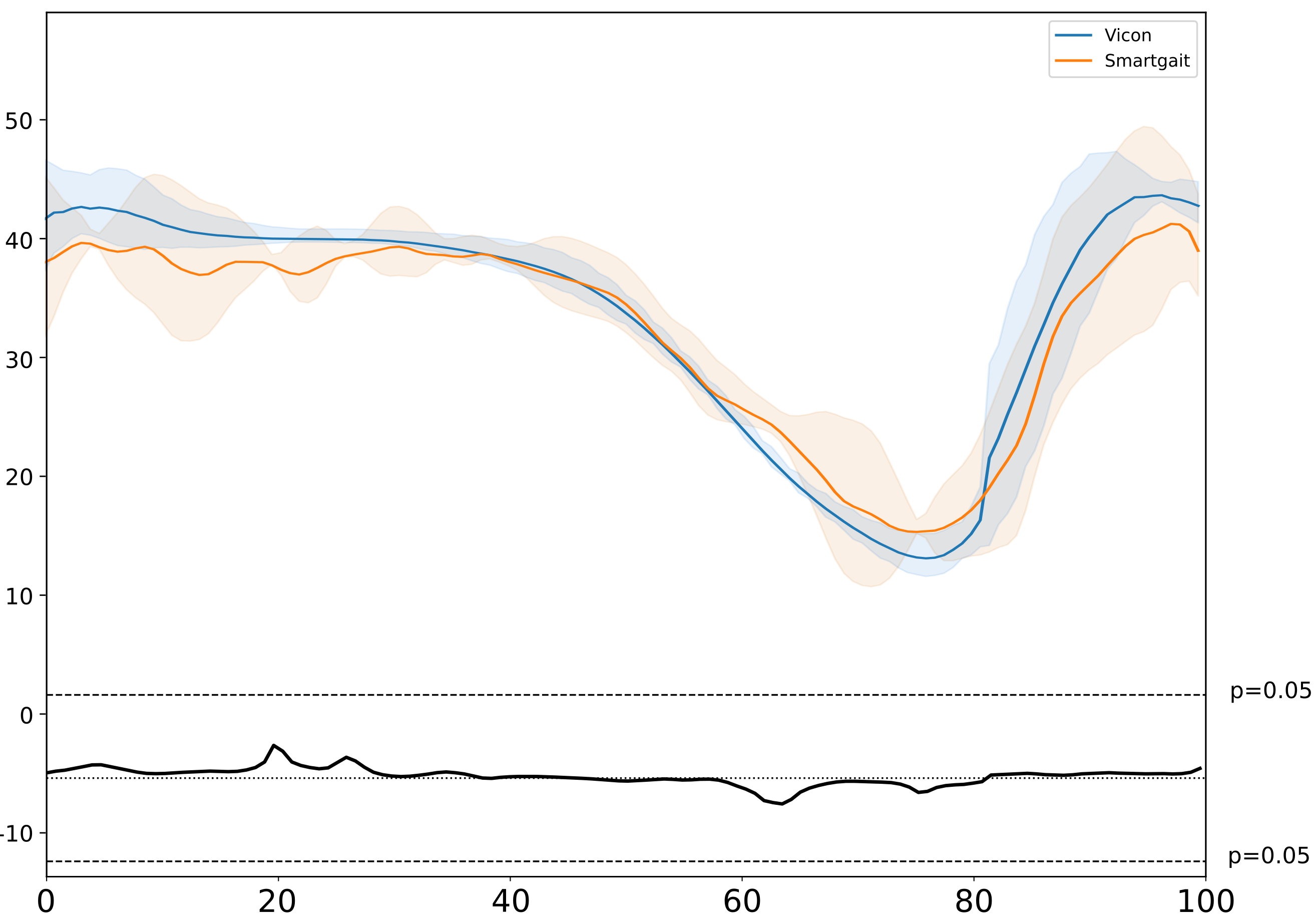

Supplement: Supplementary file 1 [file sensors-24-07819-s001.zip › spm_eval_SI18SC18_sagital/SI18SC18_angle_(2, 5, 12, 0)2.csv_plot_spm_fixed_.png]

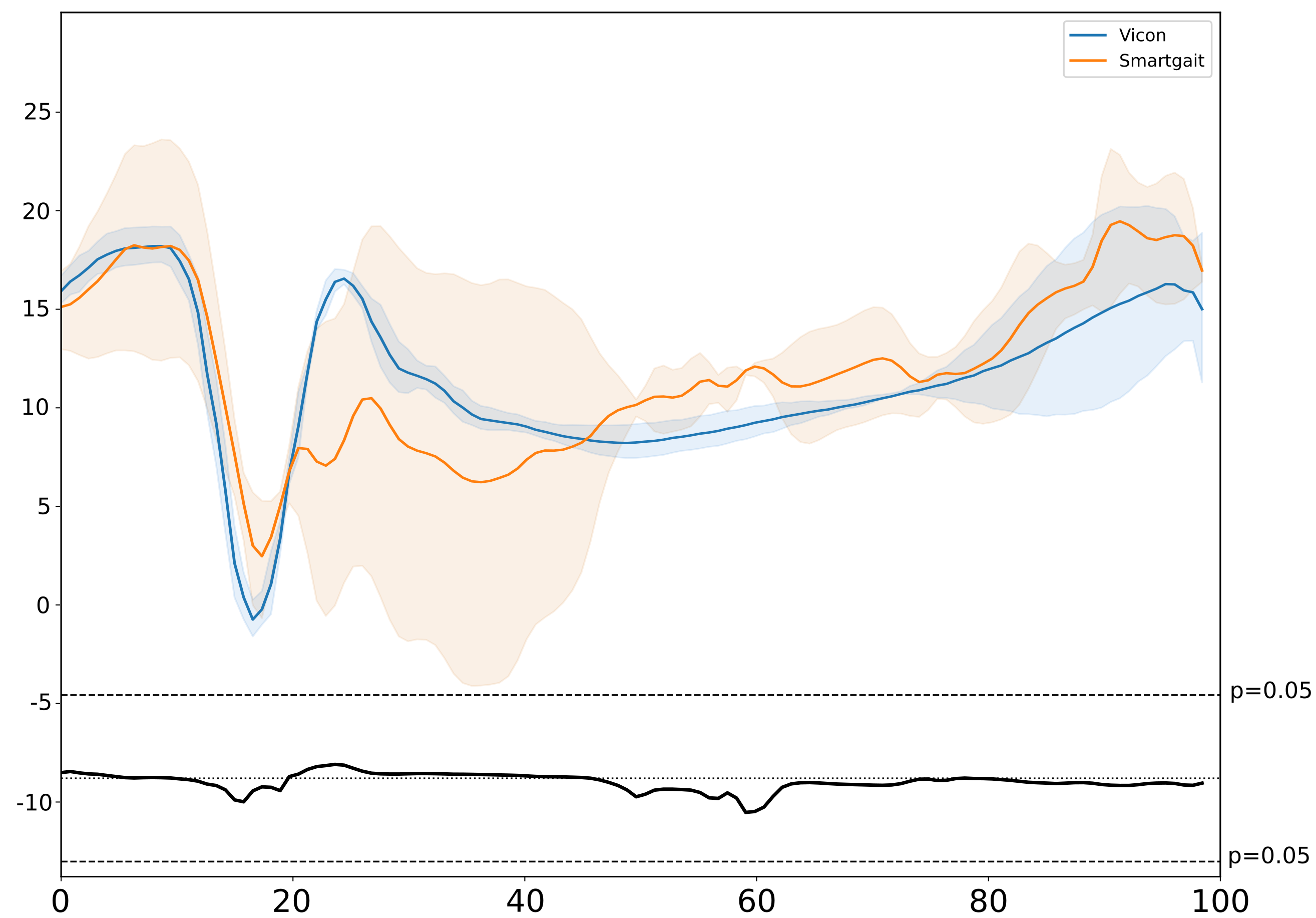

Supplement: Supplementary file 1 [file sensors-24-07819-s001.zip › spm_eval_SI18SC18_sagital/SI18SC18_angle_(5, 8, 8, 11)3.csv_plot_spm_fixed_.png]
