# Supplementary figures and images for: Steps to Facilitate the Use of Clinical Gait Analysis in Stroke Patients: The Validation of a Single 2D RGB Smartphone Video-Based System for Gait Analysis (part 1 of 2)
Source: Sensors (Basel). 2024 Dec 6;24(23):7819. doi: 10.3390/s24237819 (PMC11644854; doi:10.3390/s24237819)

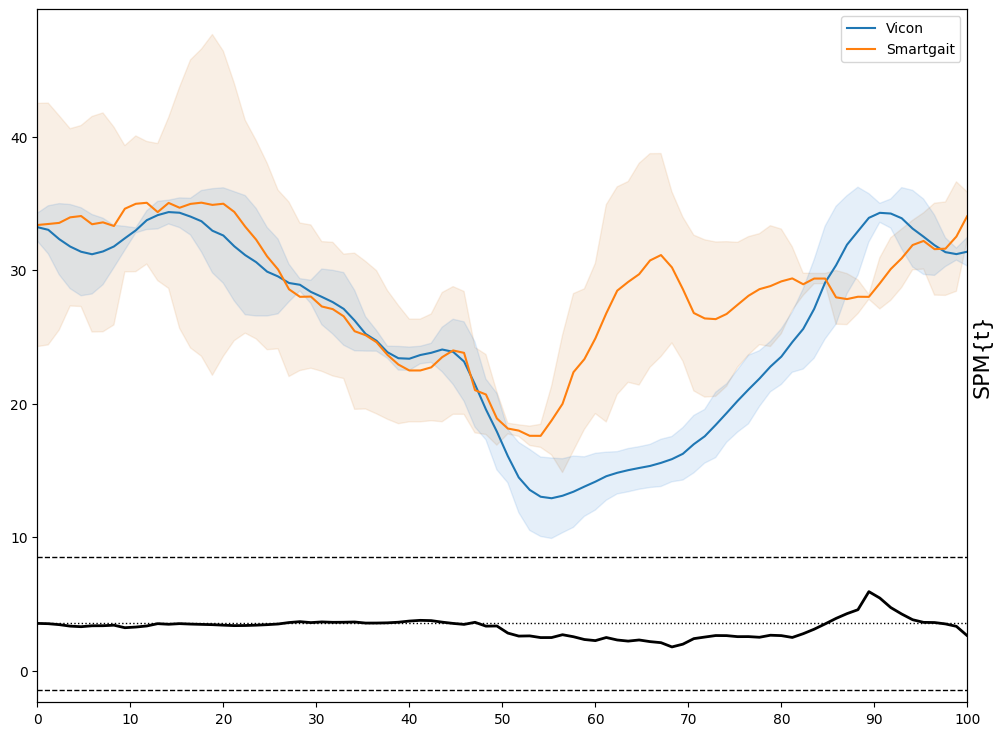

Supplement: Supplementary file 1 [file sensors-24-07819-s001.zip › spm_eval_EU28ÜH31_frontal/EU28ÜH31_angle_(2, 5, 12, 0)1.csv_plot_spm.png]

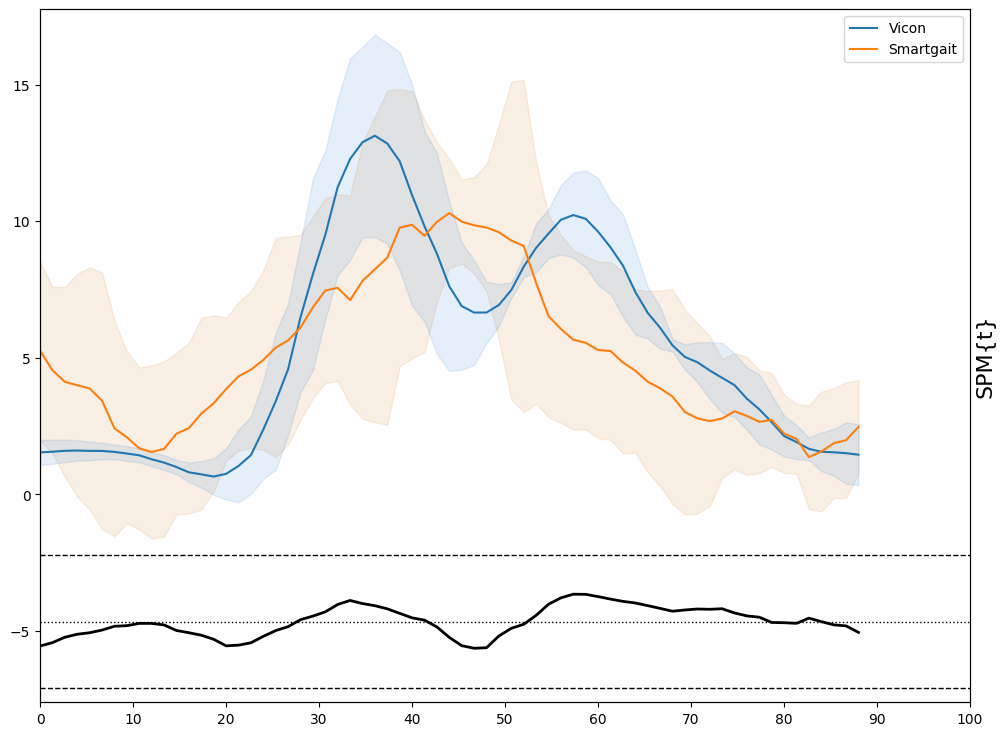

Supplement: Supplementary file 1 [file sensors-24-07819-s001.zip › spm_eval_EU28ÜH31_frontal/EU28ÜH31_angle_(2, 5, 5, 8)3.csv_plot_spm.png]

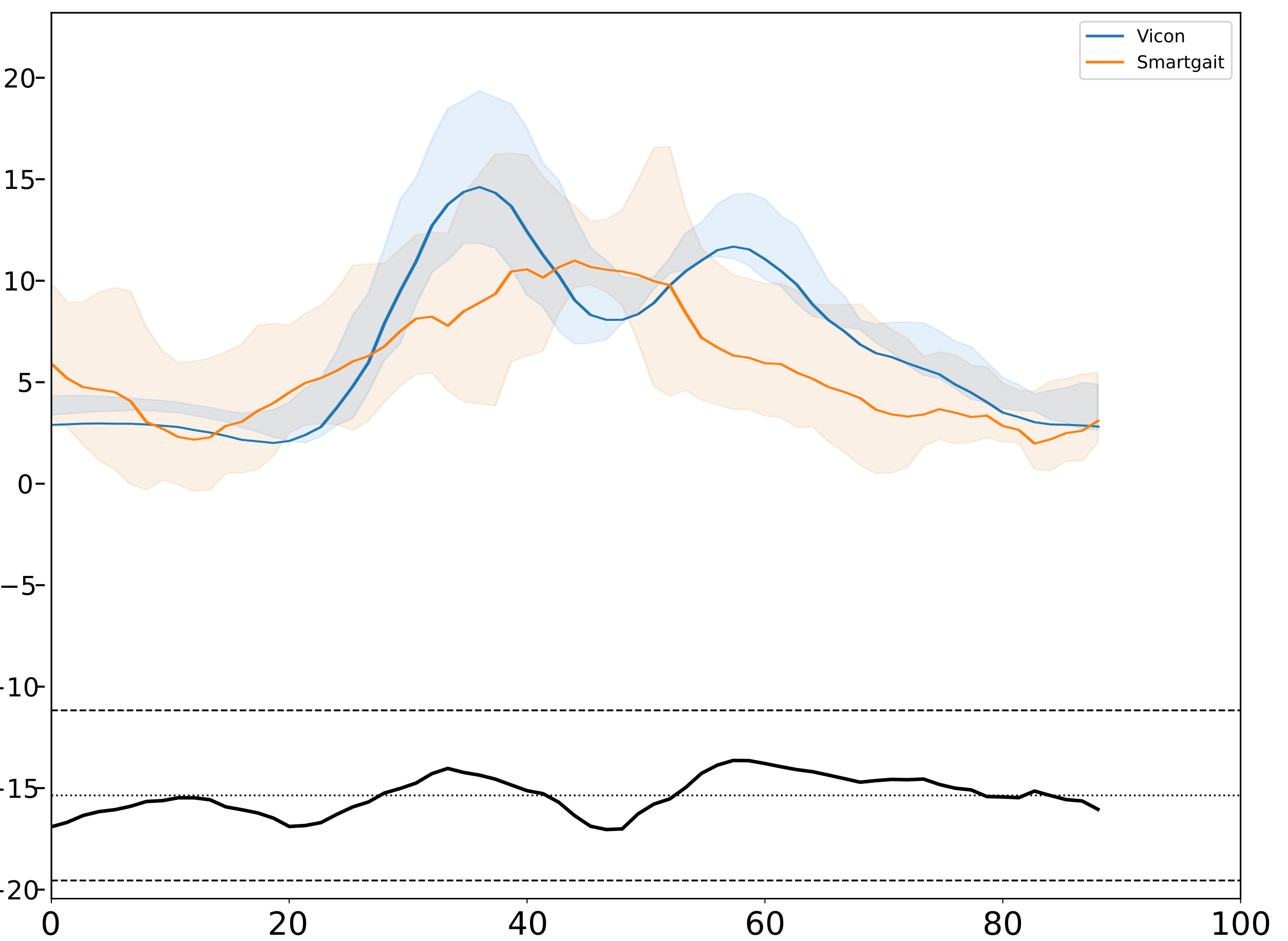

Supplement: Supplementary file 1 [file sensors-24-07819-s001.zip › spm_eval_EU28ÜH31_frontal/EU28ÜH31_angle_(2, 5, 5, 8)3.csv_plot_spm_fixed.png]

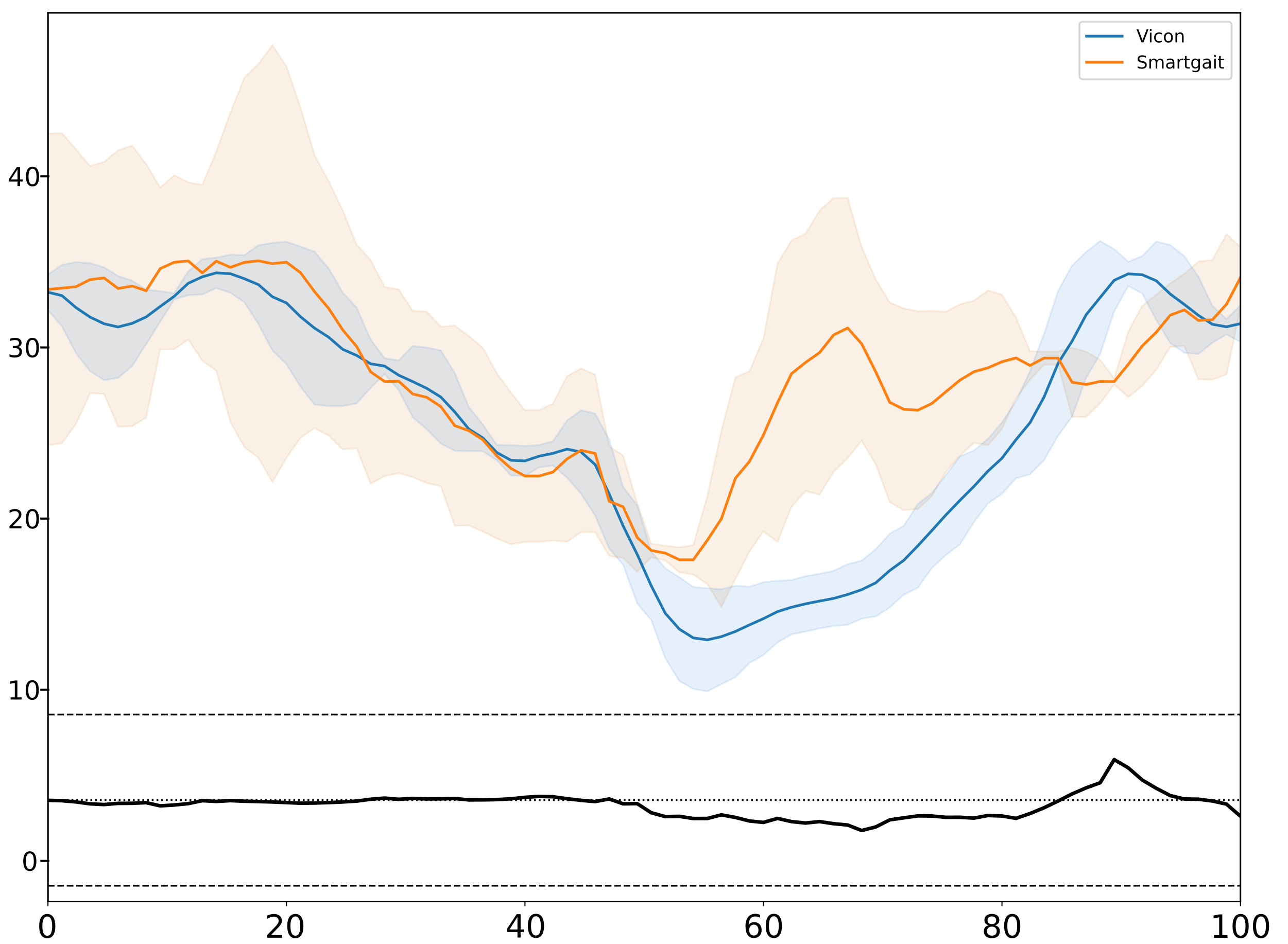

Supplement: Supplementary file 1 [file sensors-24-07819-s001.zip › spm_eval_EU28ÜH31_frontal/EU28ÜH31_angle_(2, 5, 12, 0)1.csv_plot_spm_fixed.png]

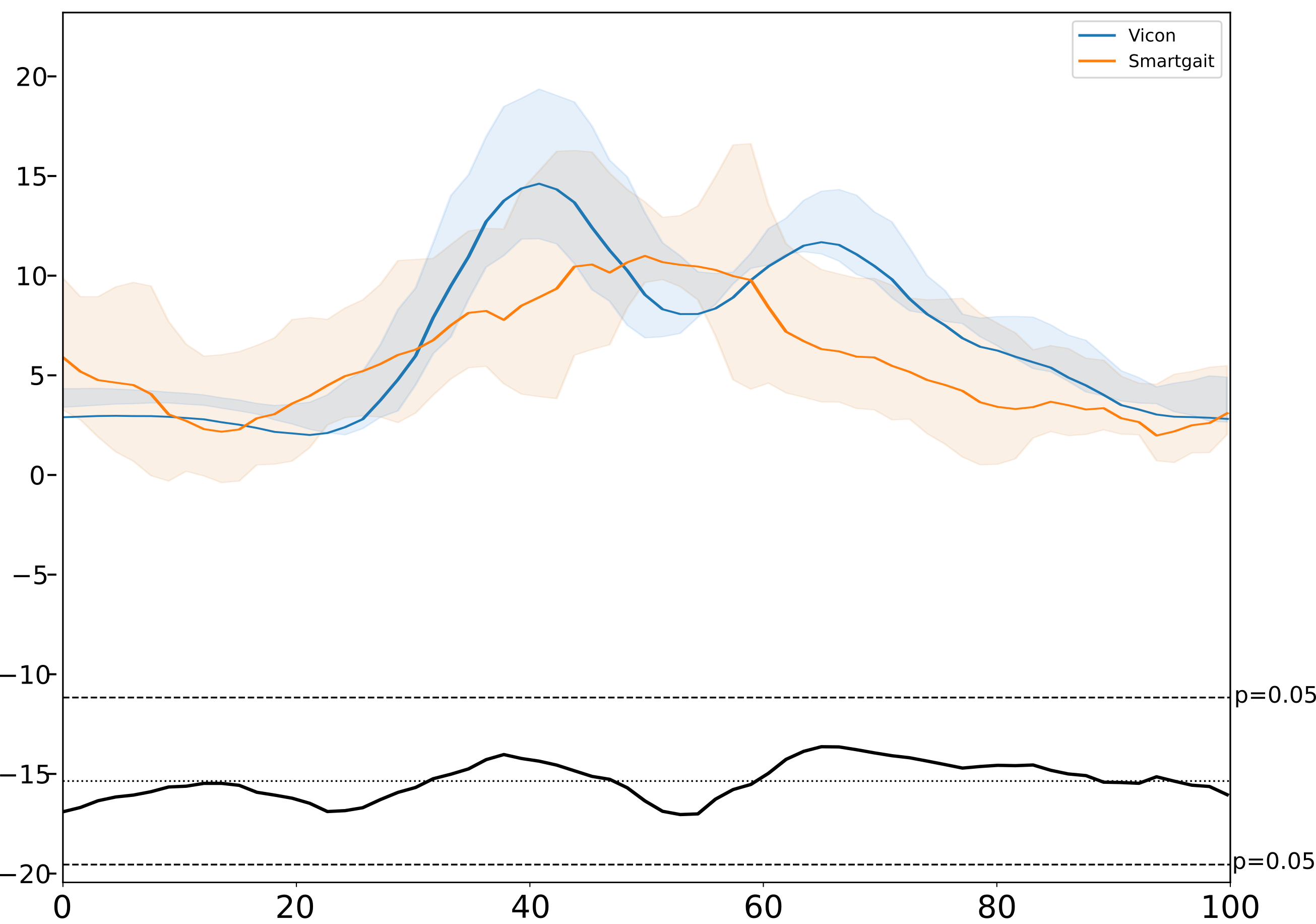

Supplement: Supplementary file 1 [file sensors-24-07819-s001.zip › spm_eval_EU28ÜH31_frontal/EU28ÜH31_angle_(2, 5, 5, 8)3.csv_plot_spm_fixed_.png]

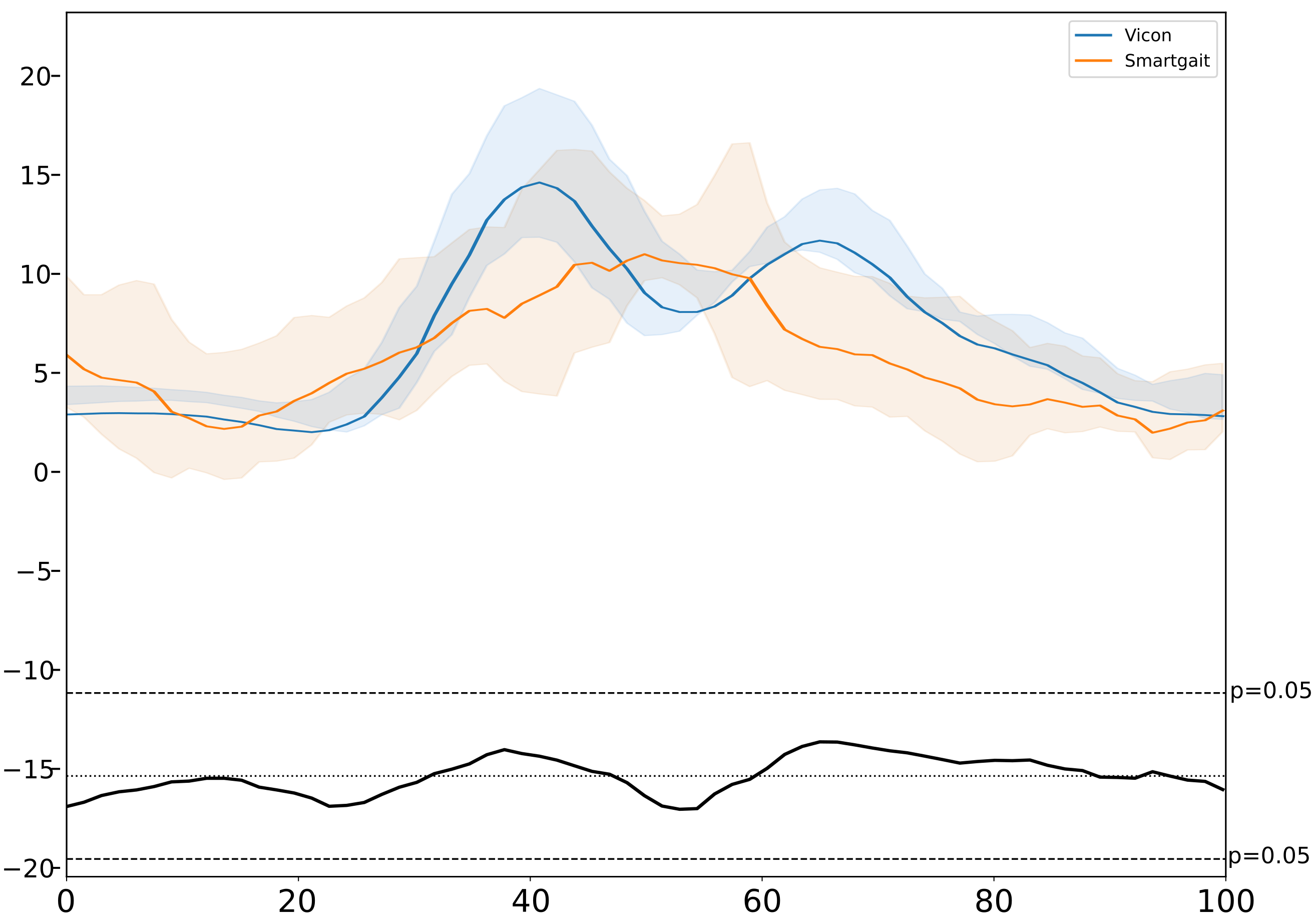

Supplement: Supplementary file 1 [file sensors-24-07819-s001.zip › spm_eval_EU28ÜH31_frontal/patch_1.png]

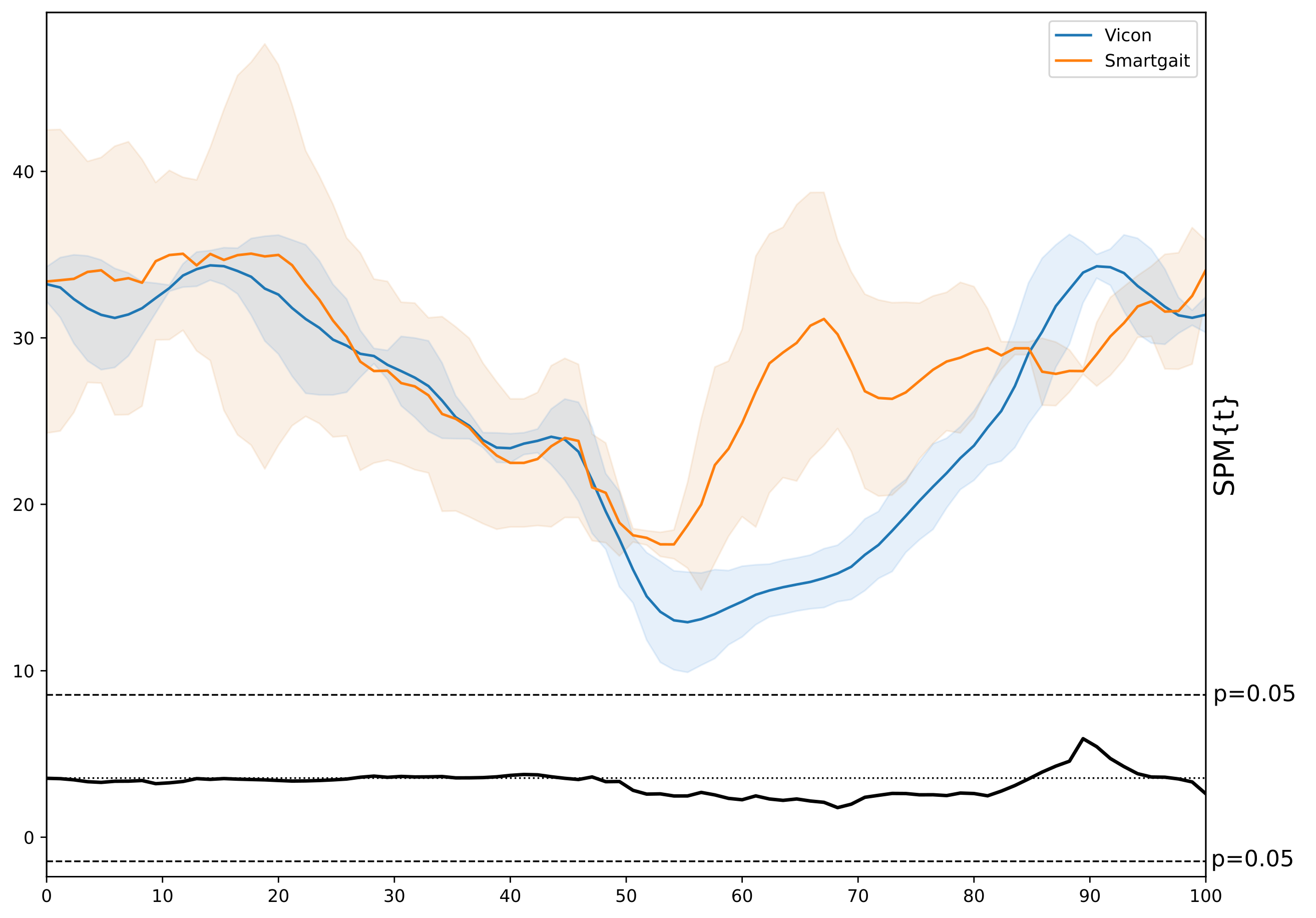

Supplement: Supplementary file 1 [file sensors-24-07819-s001.zip › spm_eval_EU28ÜH31_frontal/EU28ÜH31_angle_(2, 5, 12, 0)1.csv_plot_spm_.png]

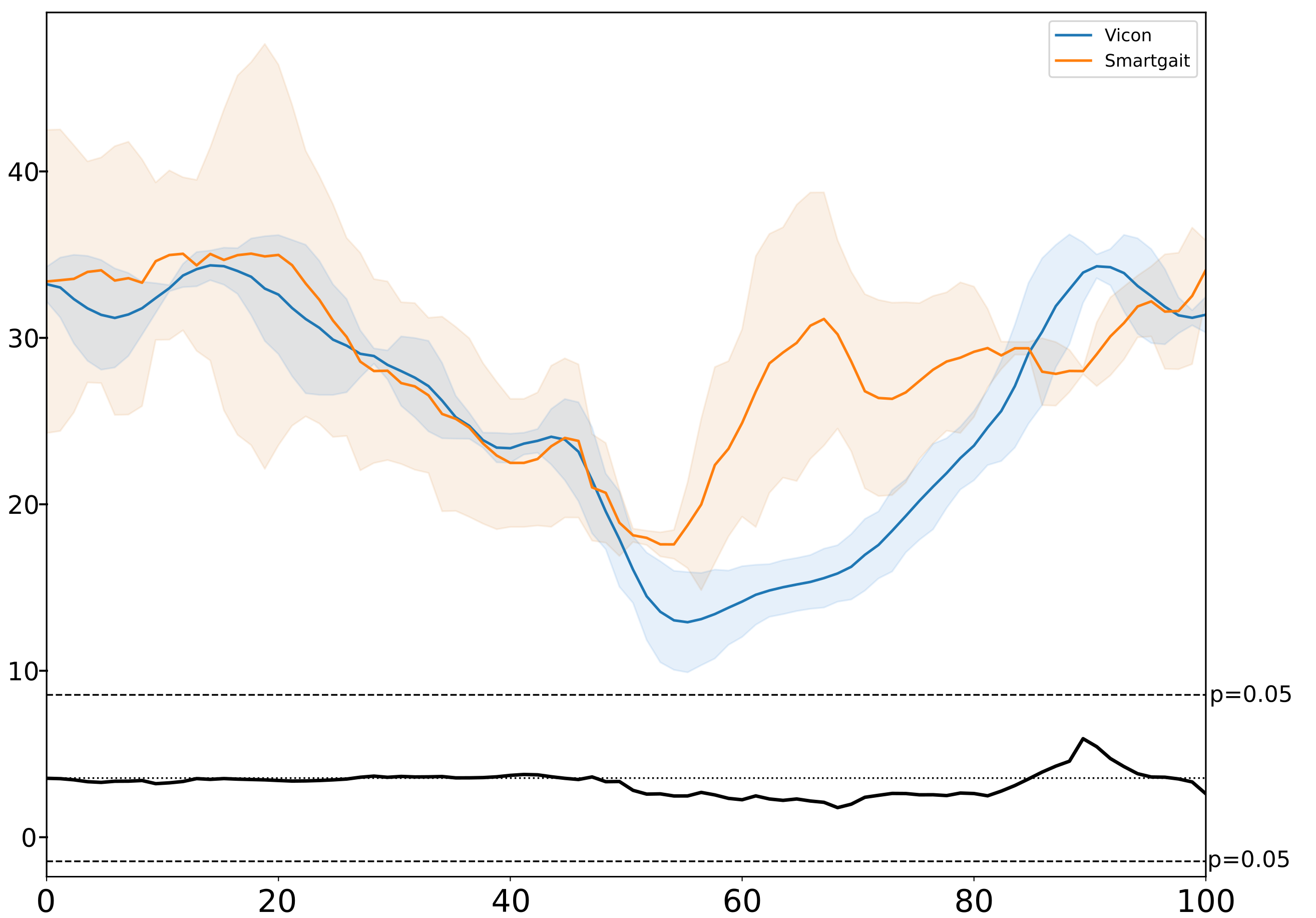

Supplement: Supplementary file 1 [file sensors-24-07819-s001.zip › spm_eval_EU28ÜH31_frontal/EU28ÜH31_angle_(2, 5, 12, 0)1.csv_plot_spm_fixed_.png]

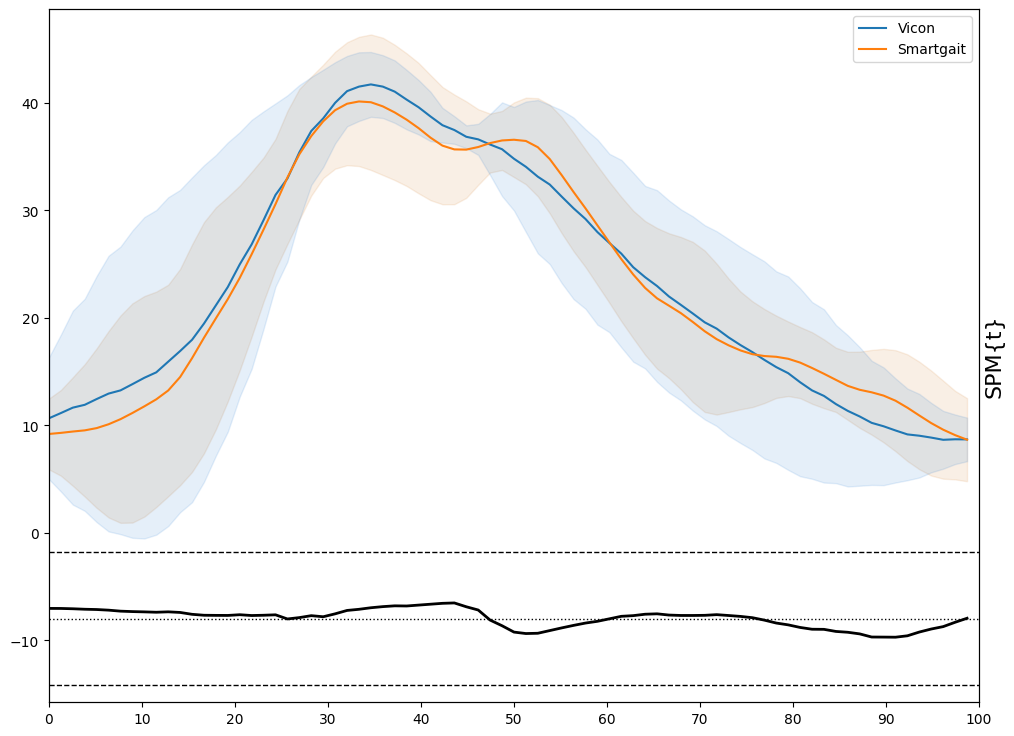

Supplement: Supplementary file 1 [file sensors-24-07819-s001.zip › spm_eval_EU28ÜH31_sagital/EU28ÜH31_angle_(2, 5, 5, 8)1.csv_plot_spm.png]

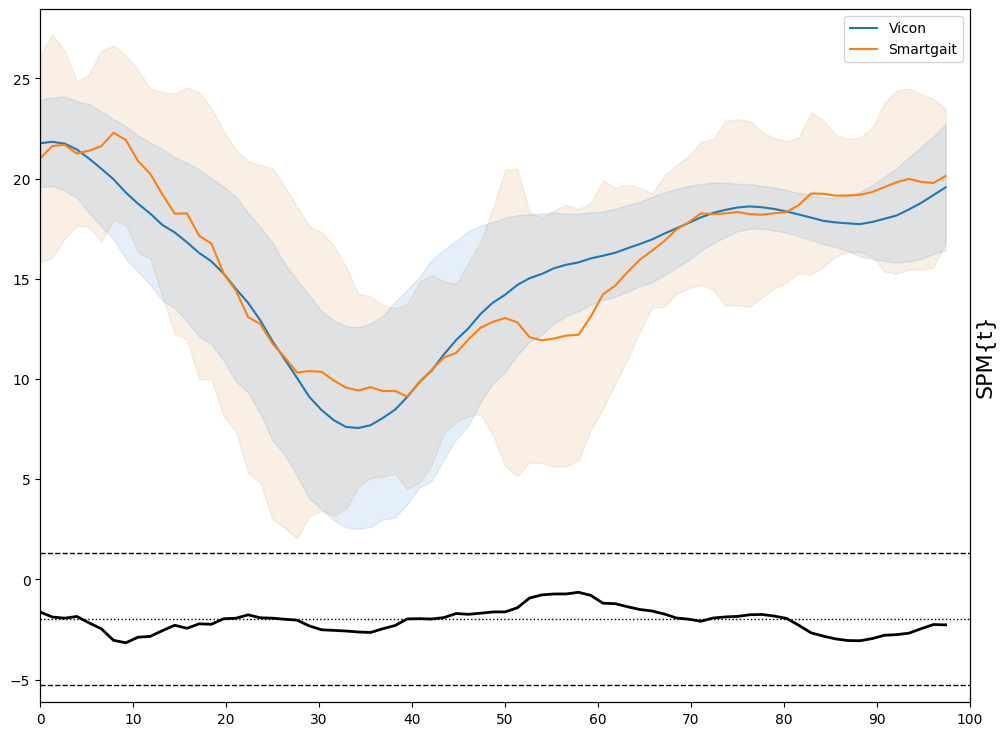

Supplement: Supplementary file 1 [file sensors-24-07819-s001.zip › spm_eval_EU28ÜH31_sagital/EU28ÜH31_angle_(2, 5, 12, 0)2.csv_plot_spm.png]

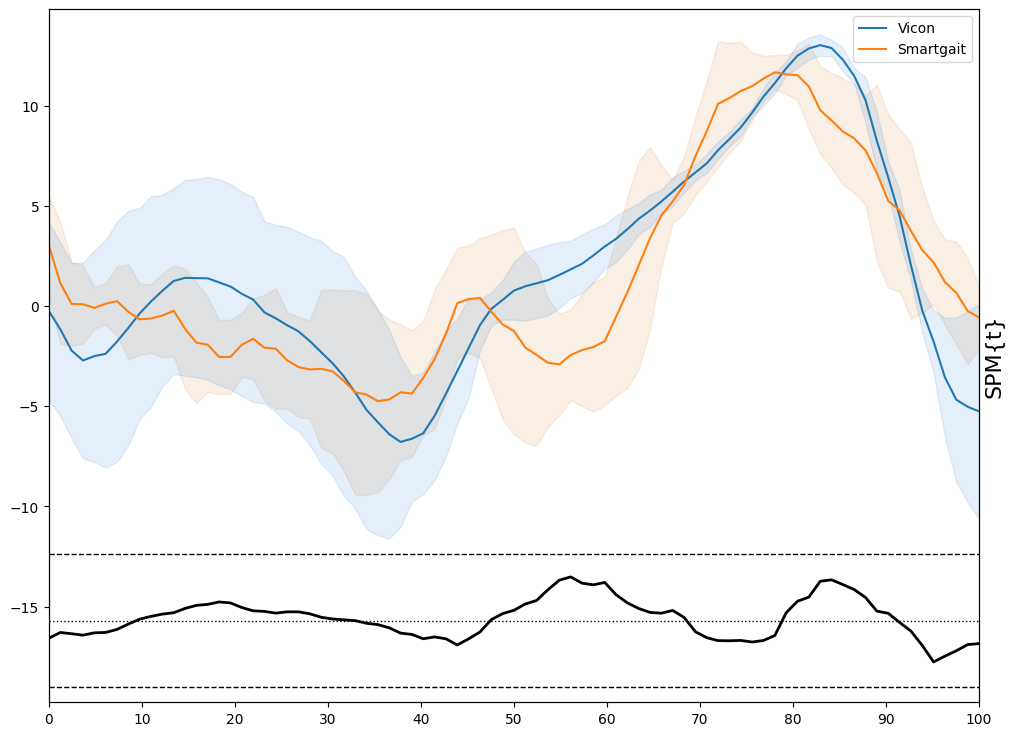

Supplement: Supplementary file 1 [file sensors-24-07819-s001.zip › spm_eval_EU28ÜH31_sagital/EU28ÜH31_angle_(5, 8, 8, 11)4.csv_plot_spm.png]

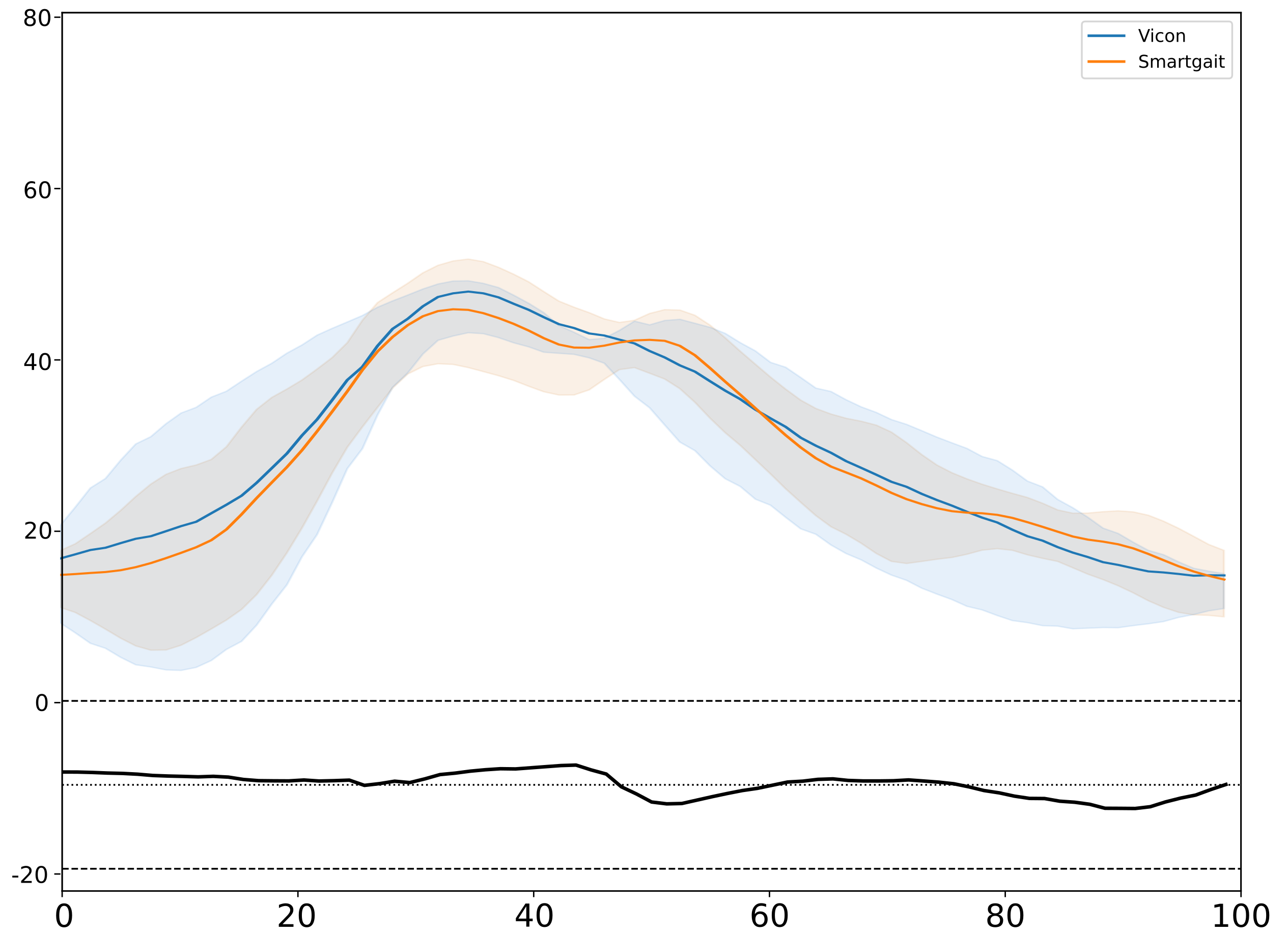

Supplement: Supplementary file 1 [file sensors-24-07819-s001.zip › spm_eval_EU28ÜH31_sagital/EU28ÜH31_angle_(2, 5, 5, 8)1.csv_plot_spm_fixed.png]

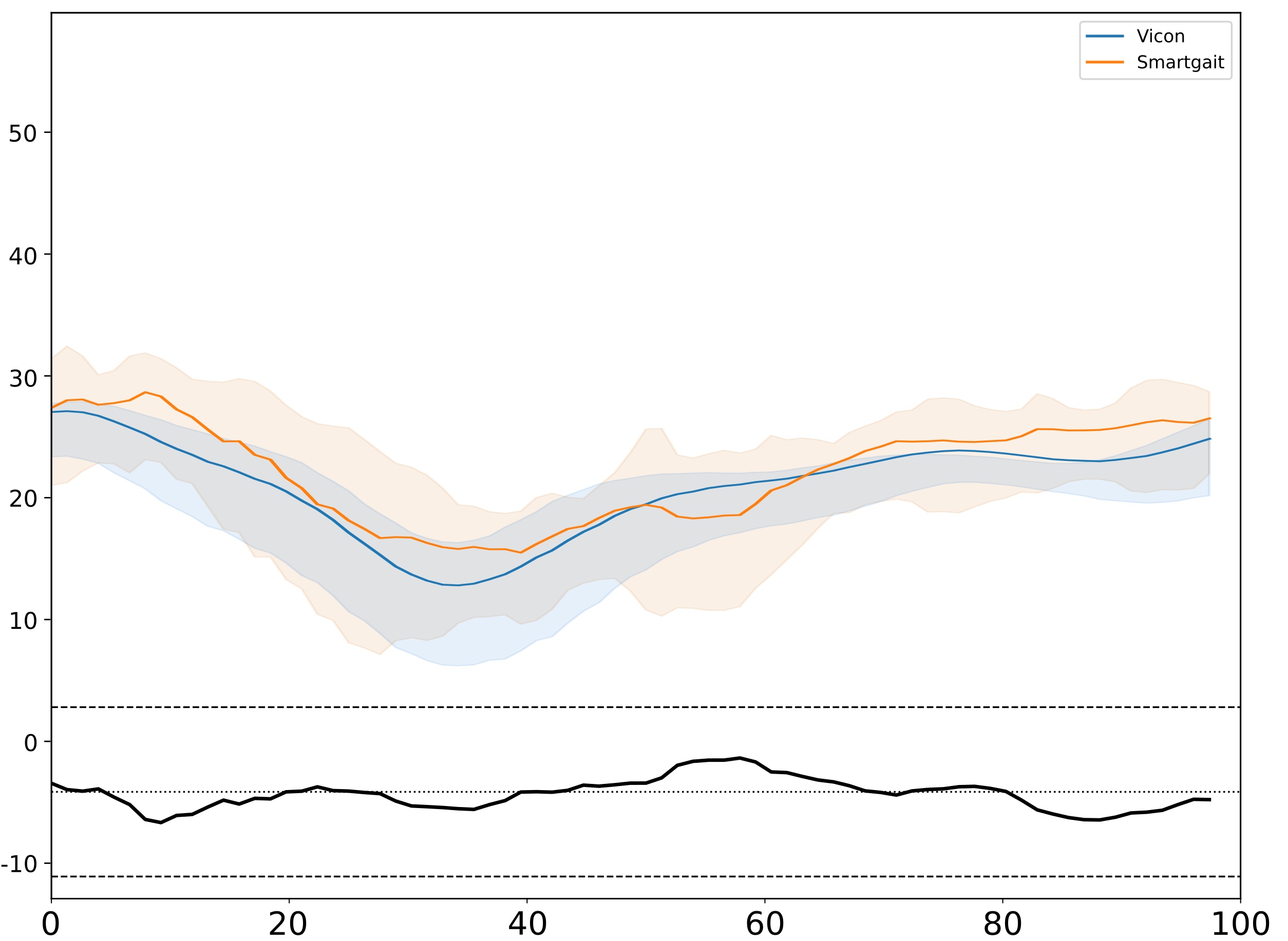

Supplement: Supplementary file 1 [file sensors-24-07819-s001.zip › spm_eval_EU28ÜH31_sagital/EU28ÜH31_angle_(2, 5, 12, 0)2.csv_plot_spm_fixed.png]

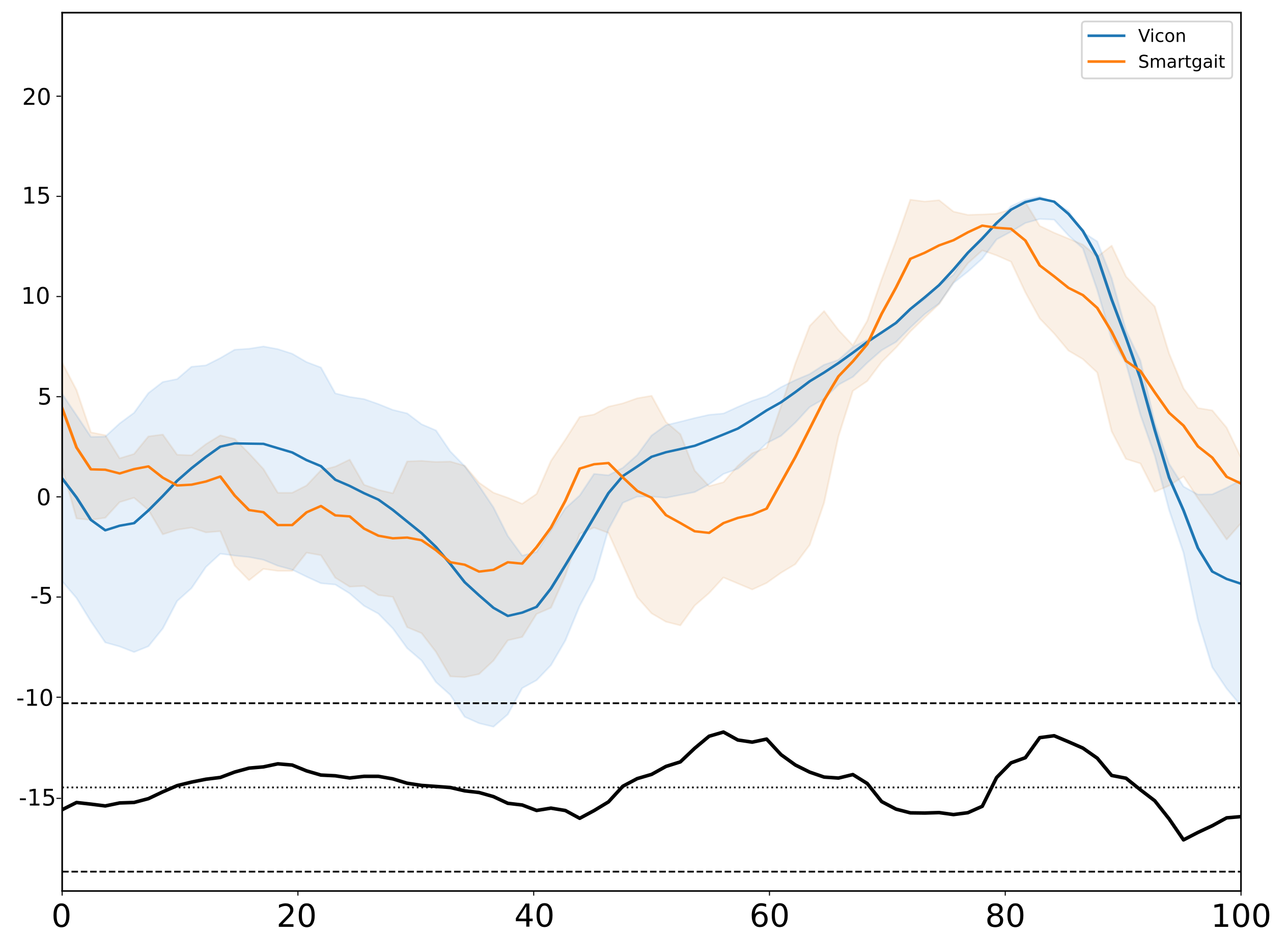

Supplement: Supplementary file 1 [file sensors-24-07819-s001.zip › spm_eval_EU28ÜH31_sagital/EU28ÜH31_angle_(5, 8, 8, 11)4.csv_plot_spm_fixed.png]

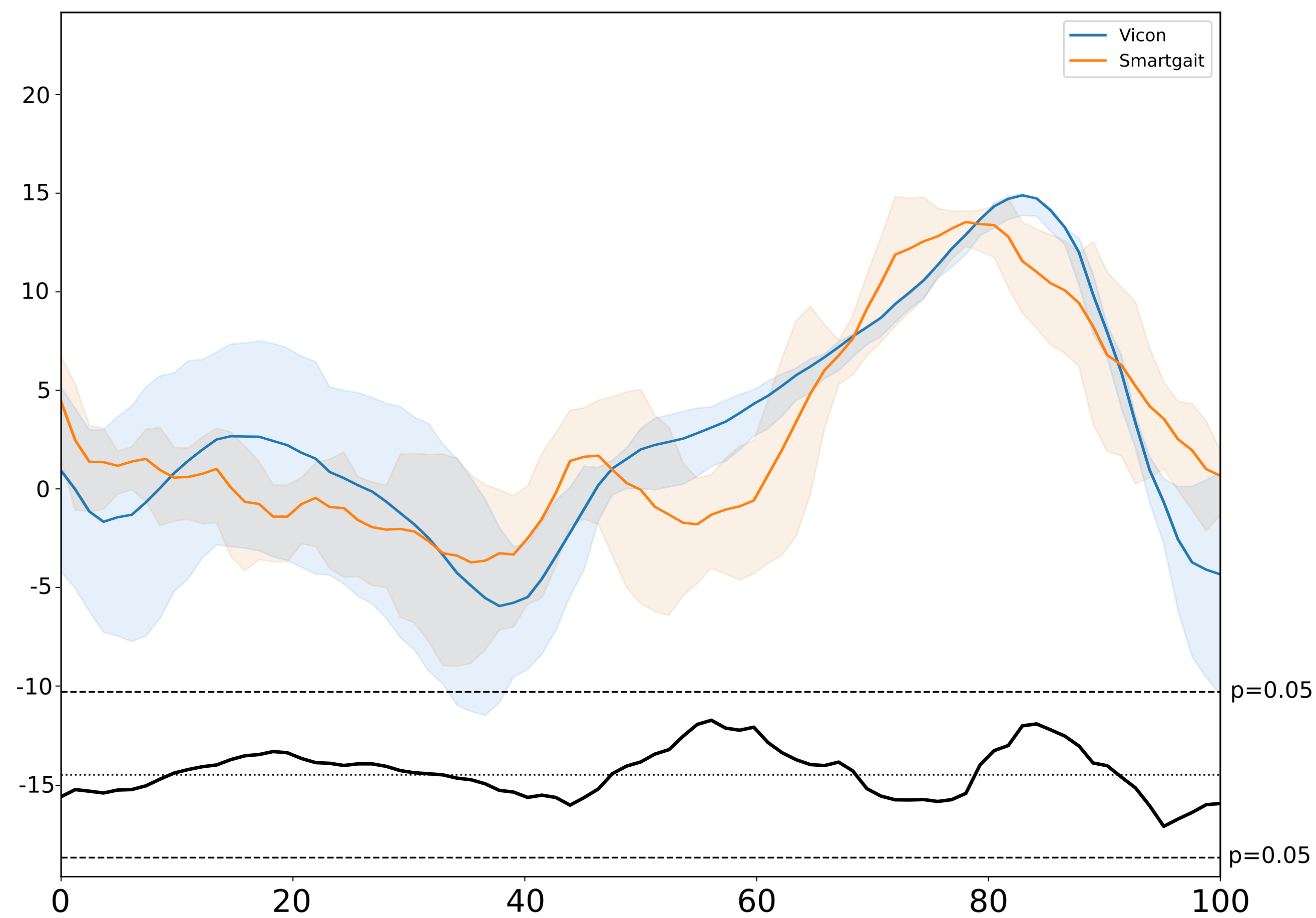

Supplement: Supplementary file 1 [file sensors-24-07819-s001.zip › spm_eval_EU28ÜH31_sagital/EU28ÜH31_angle_(5, 8, 8, 11)4.csv_plot_spm_fixed_.png]

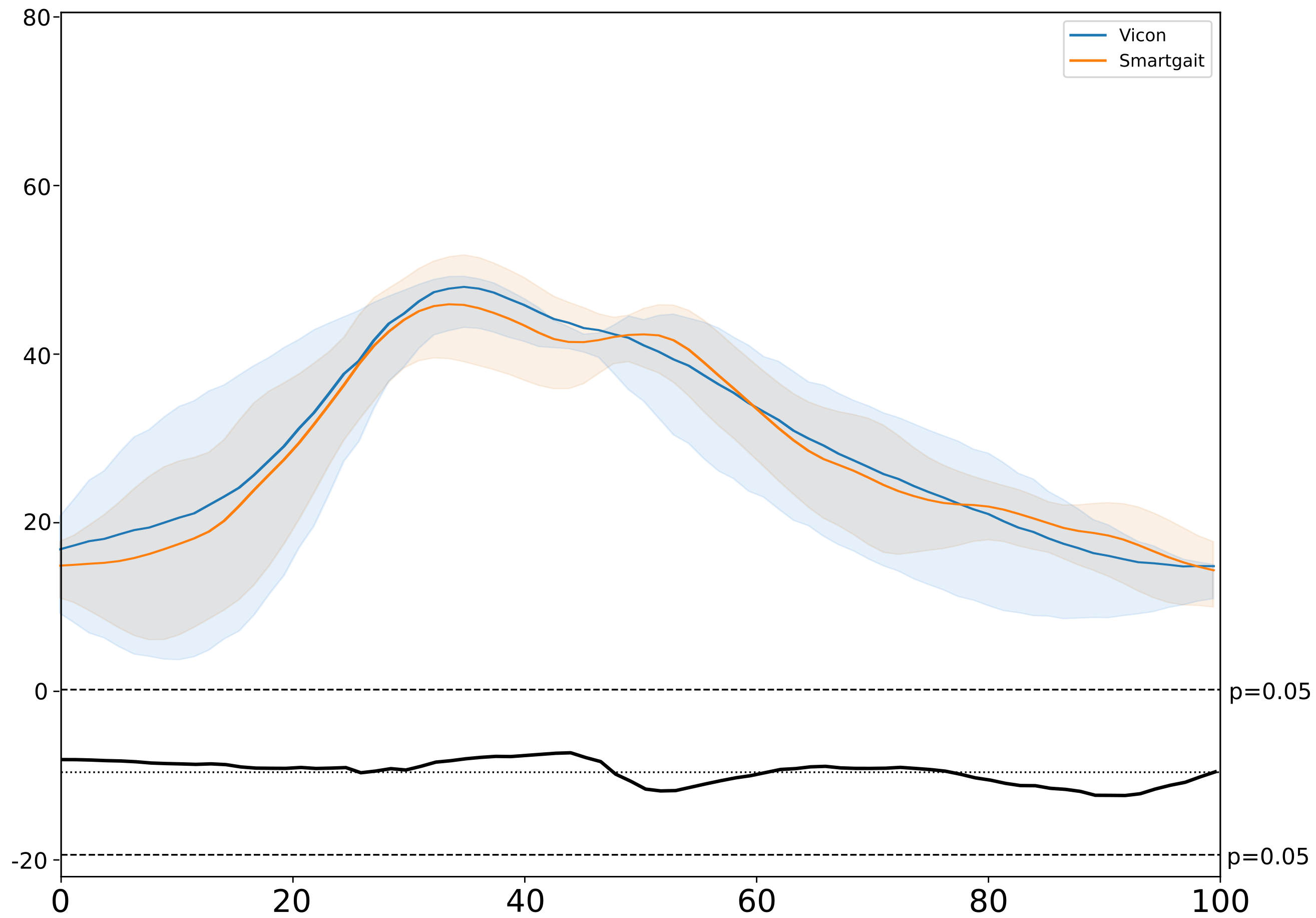

Supplement: Supplementary file 1 [file sensors-24-07819-s001.zip › spm_eval_EU28ÜH31_sagital/EU28ÜH31_angle_(2, 5, 5, 8)1.csv_plot_spm_fixed_.png]

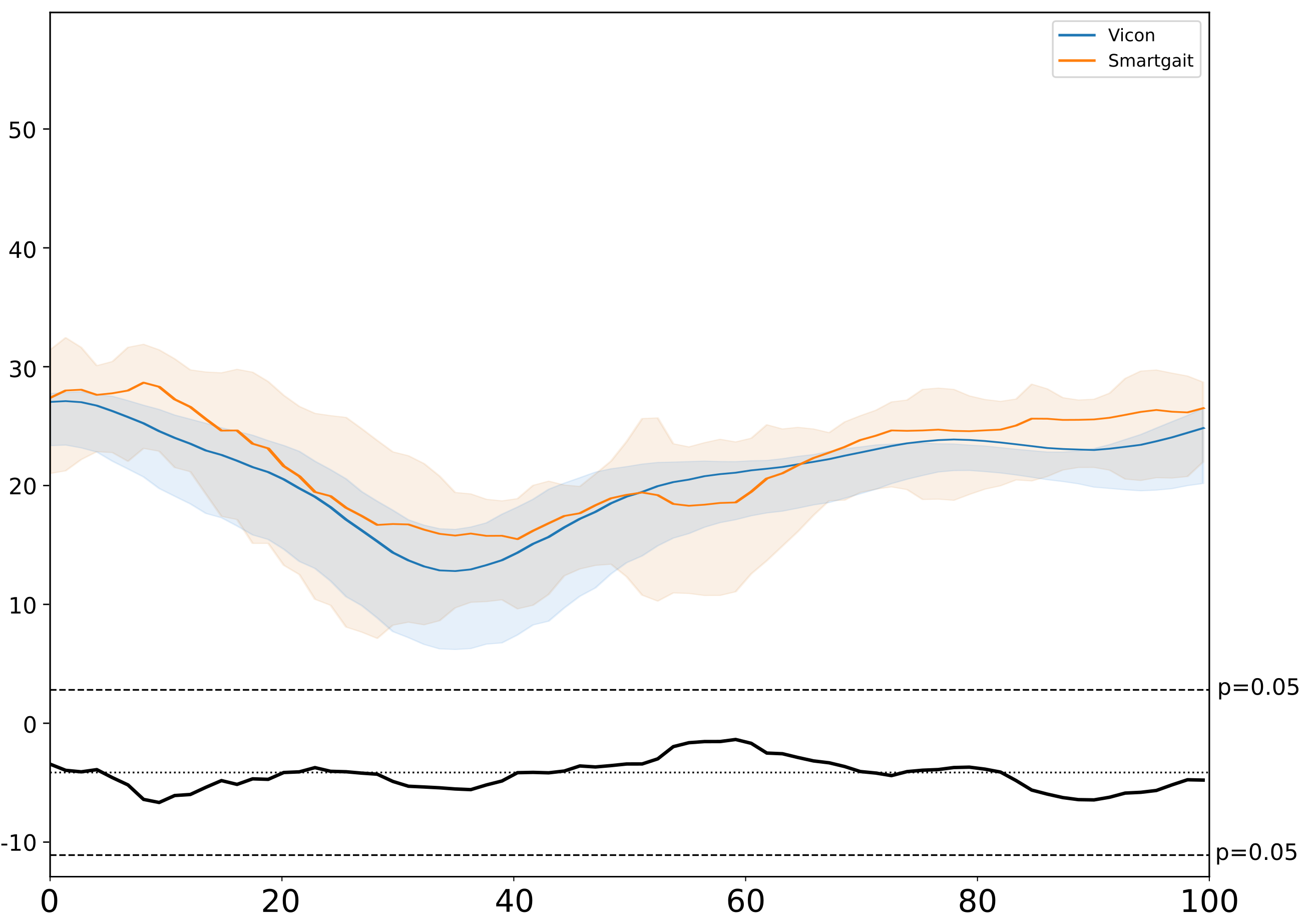

Supplement: Supplementary file 1 [file sensors-24-07819-s001.zip › spm_eval_EU28ÜH31_sagital/EU28ÜH31_angle_(2, 5, 12, 0)2.csv_plot_spm_fixed_.png]

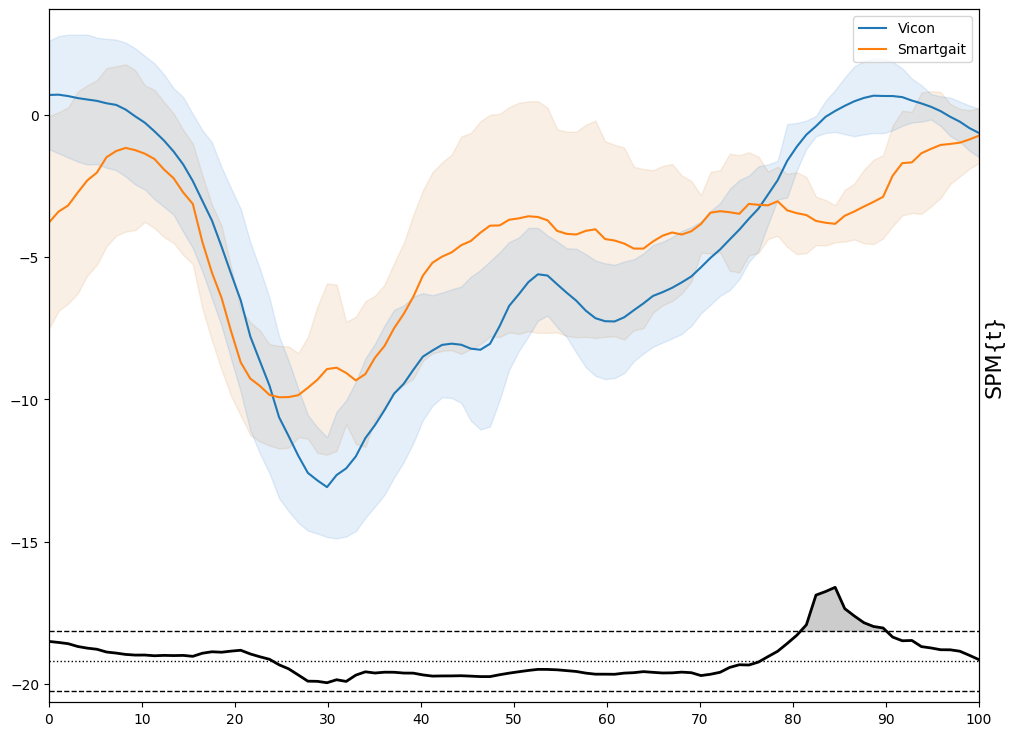

Supplement: Supplementary file 1 [file sensors-24-07819-s001.zip › spm_eval_FI11SI0801_frontal/FI11SI0801_angle_(2, 5, 12, 0)0.csv_plot_spm.png]

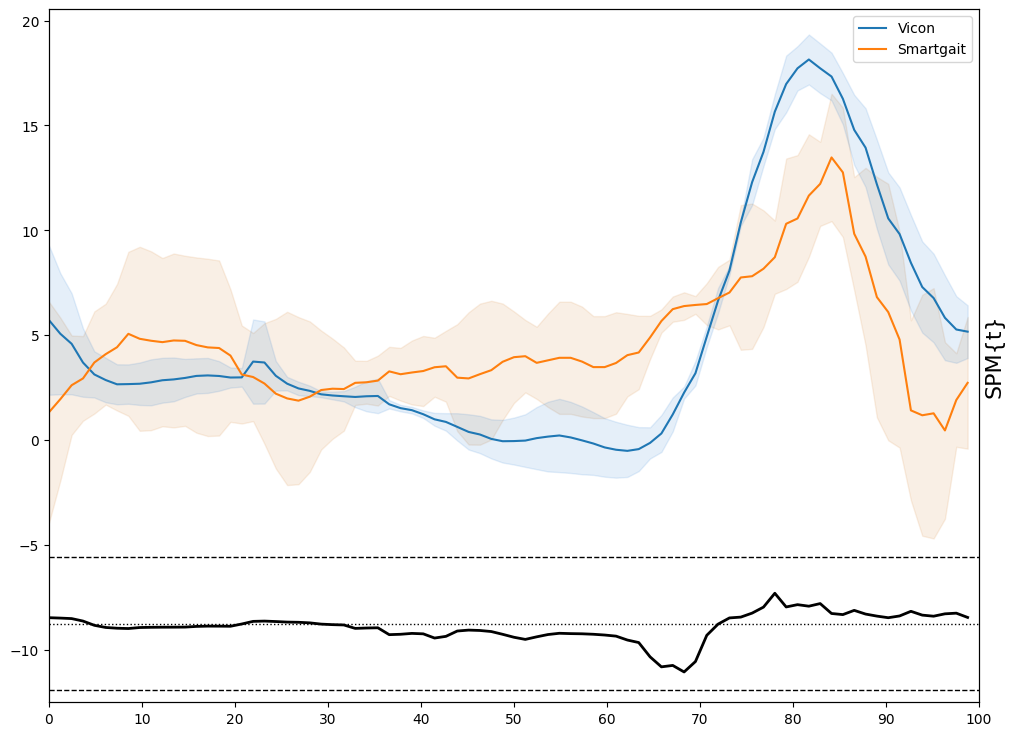

Supplement: Supplementary file 1 [file sensors-24-07819-s001.zip › spm_eval_FI11SI0801_frontal/FI11SI0801_angle_(2, 5, 5, 8)2.csv_plot_spm.png]

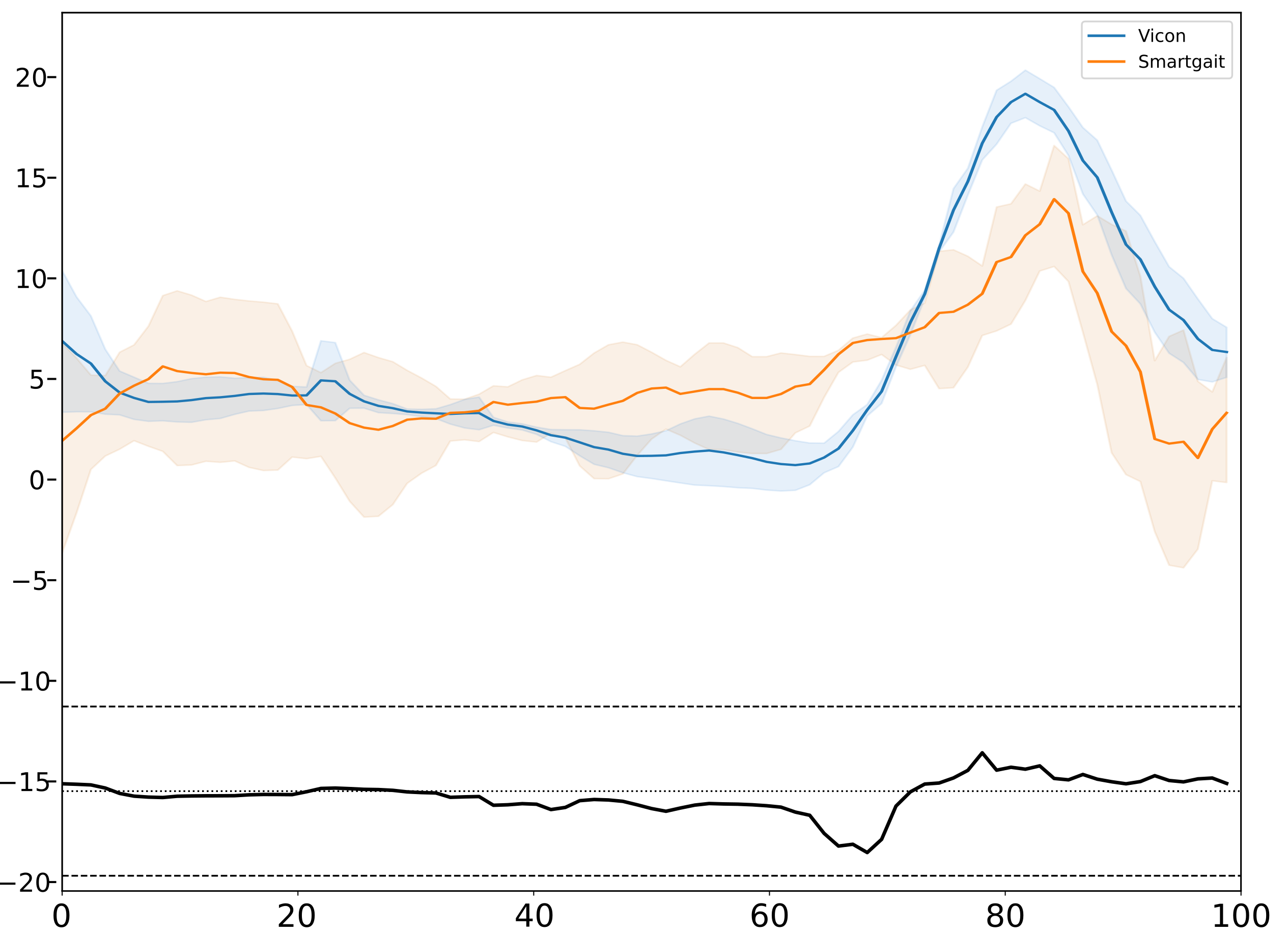

Supplement: Supplementary file 1 [file sensors-24-07819-s001.zip › spm_eval_FI11SI0801_frontal/FI11SI0801_angle_(2, 5, 5, 8)2.csv_plot_spm_fixed.png]

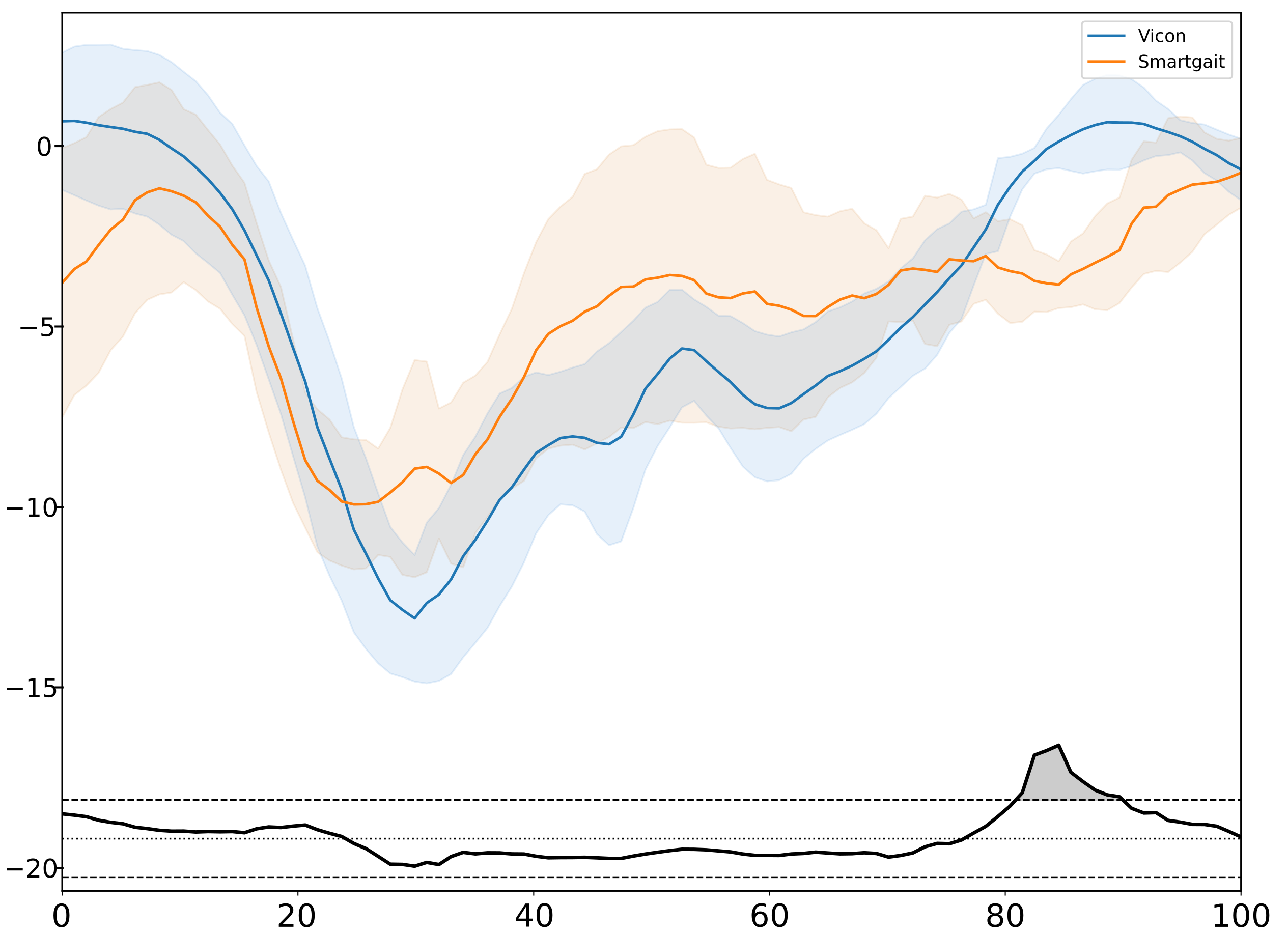

Supplement: Supplementary file 1 [file sensors-24-07819-s001.zip › spm_eval_FI11SI0801_frontal/FI11SI0801_angle_(2, 5, 12, 0)0.csv_plot_spm_fixed.png]

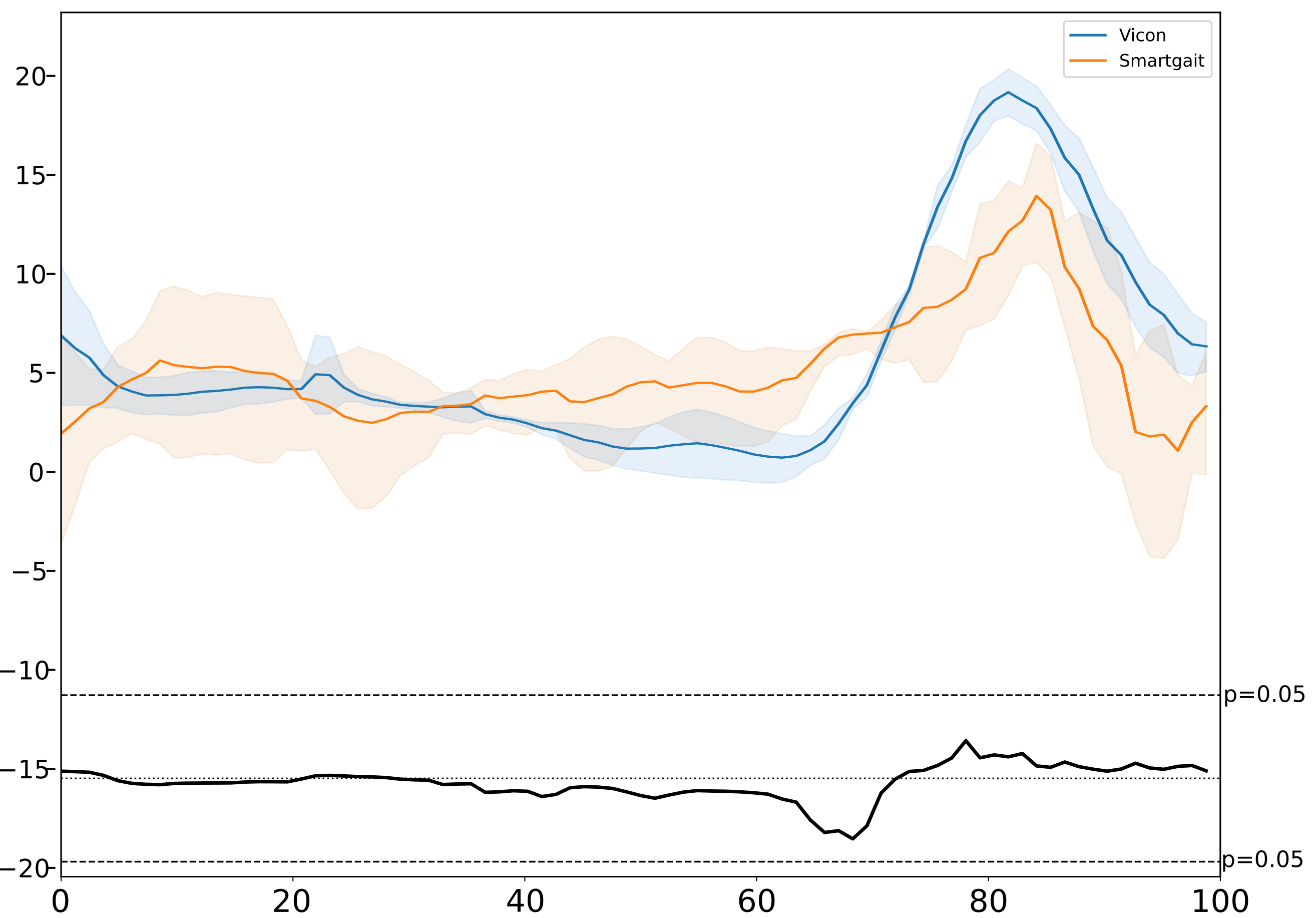

Supplement: Supplementary file 1 [file sensors-24-07819-s001.zip › spm_eval_FI11SI0801_frontal/FI11SI0801_angle_(2, 5, 5, 8)2.csv_plot_spm_fixed_.png]

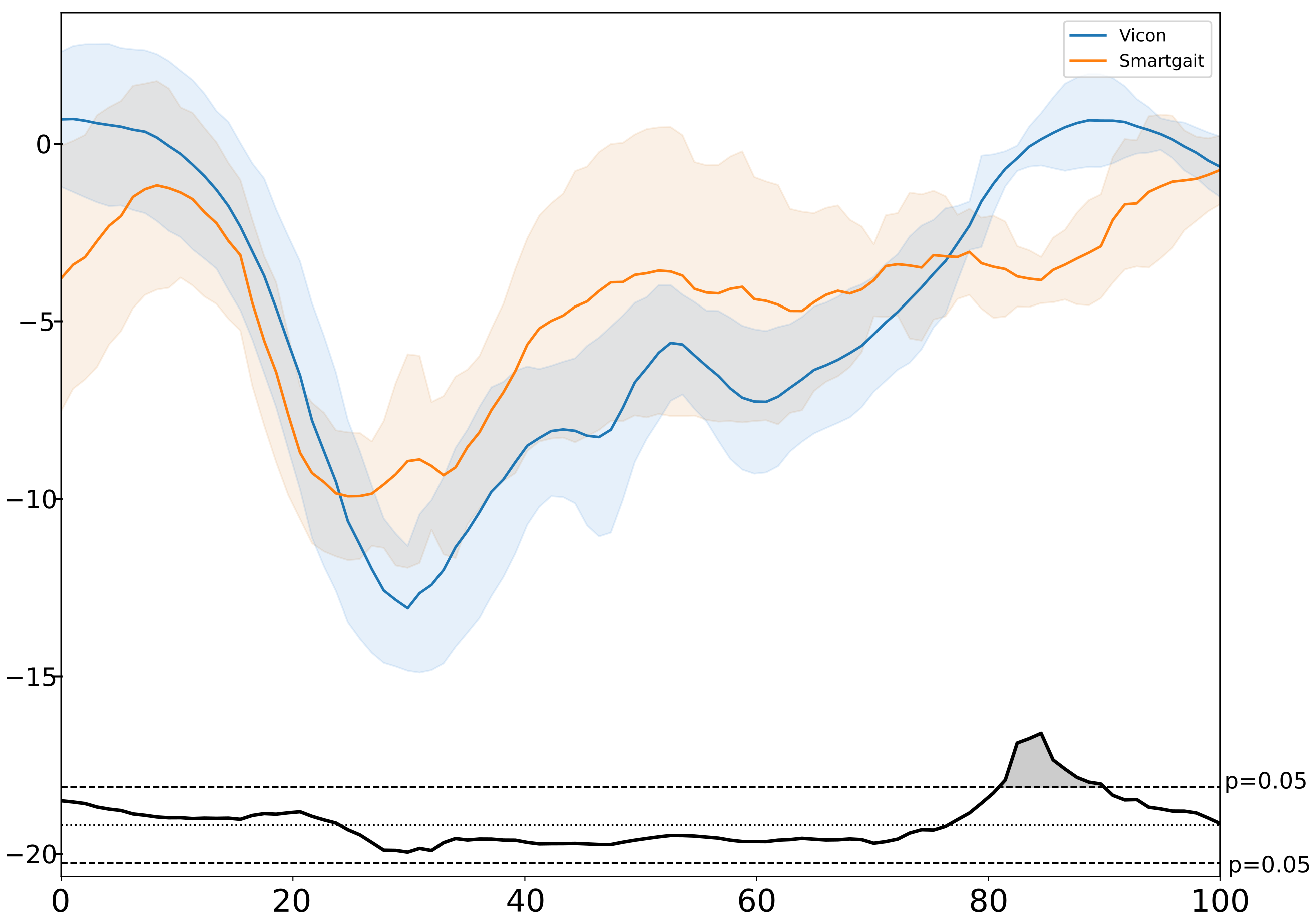

Supplement: Supplementary file 1 [file sensors-24-07819-s001.zip › spm_eval_FI11SI0801_frontal/FI11SI0801_angle_(2, 5, 12, 0)0.csv_plot_spm_fixed_.png]

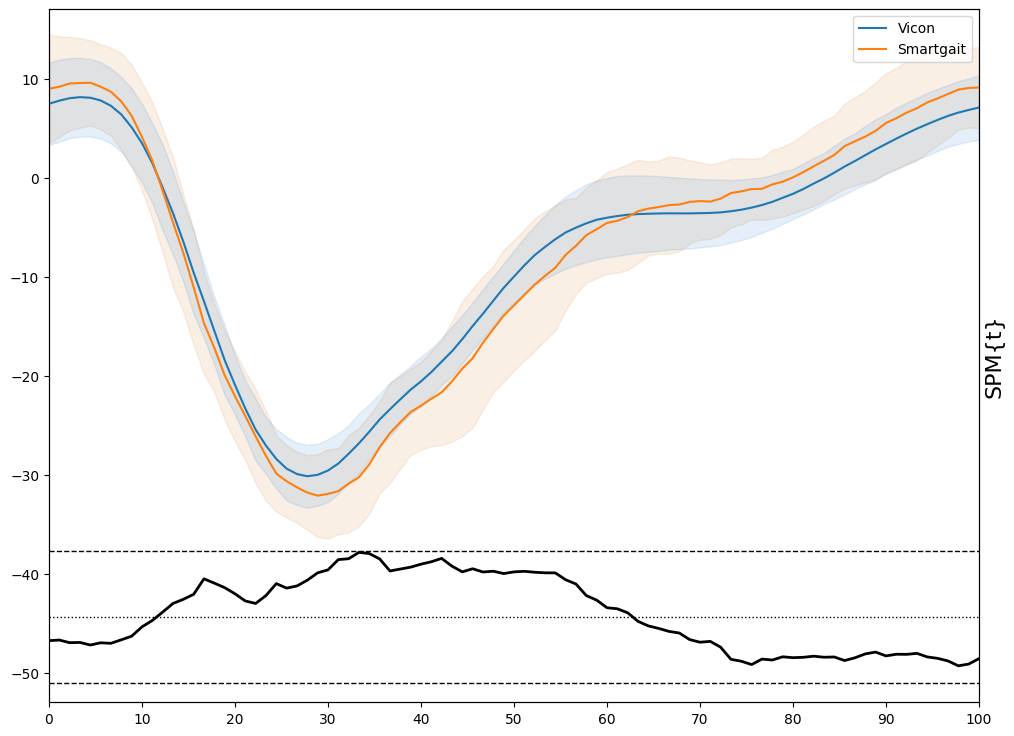

Supplement: Supplementary file 1 [file sensors-24-07819-s001.zip › spm_eval_FI11SI0801_sagital/FI11SI0801_angle_(2, 5, 12, 0)5.csv_plot_spm.png]

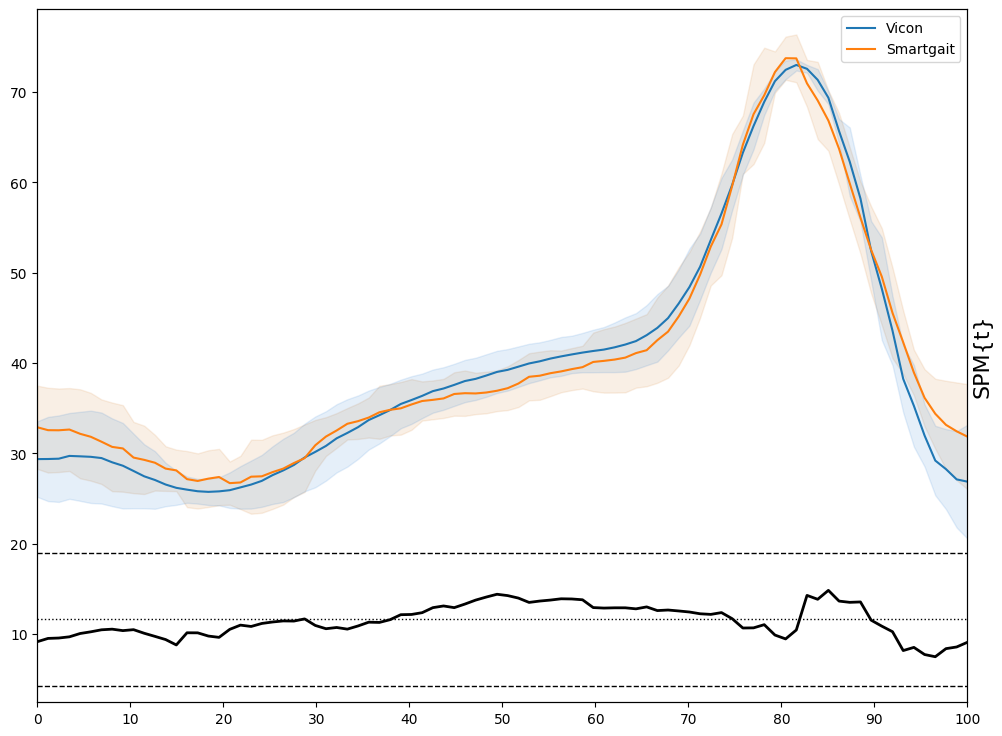

Supplement: Supplementary file 1 [file sensors-24-07819-s001.zip › spm_eval_FI11SI0801_sagital/FI11SI0801_angle_(2, 5, 5, 8)4.csv_plot_spm.png]

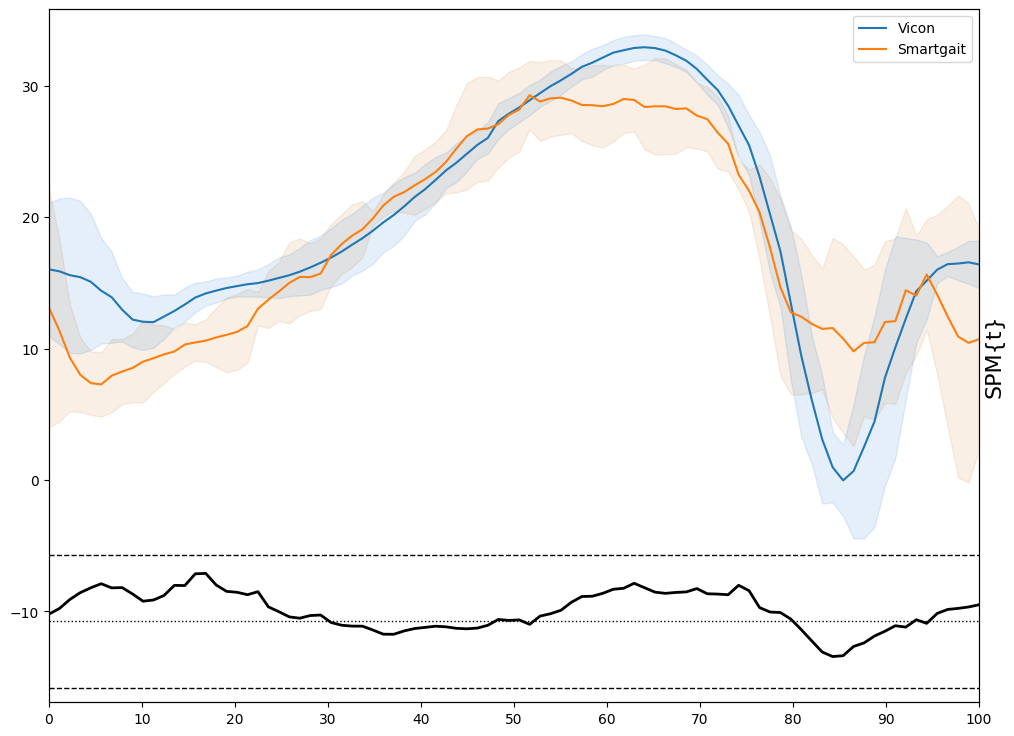

Supplement: Supplementary file 1 [file sensors-24-07819-s001.zip › spm_eval_FI11SI0801_sagital/FI11SI0801_angle_(5, 8, 8, 11)1.csv_plot_spm.png]

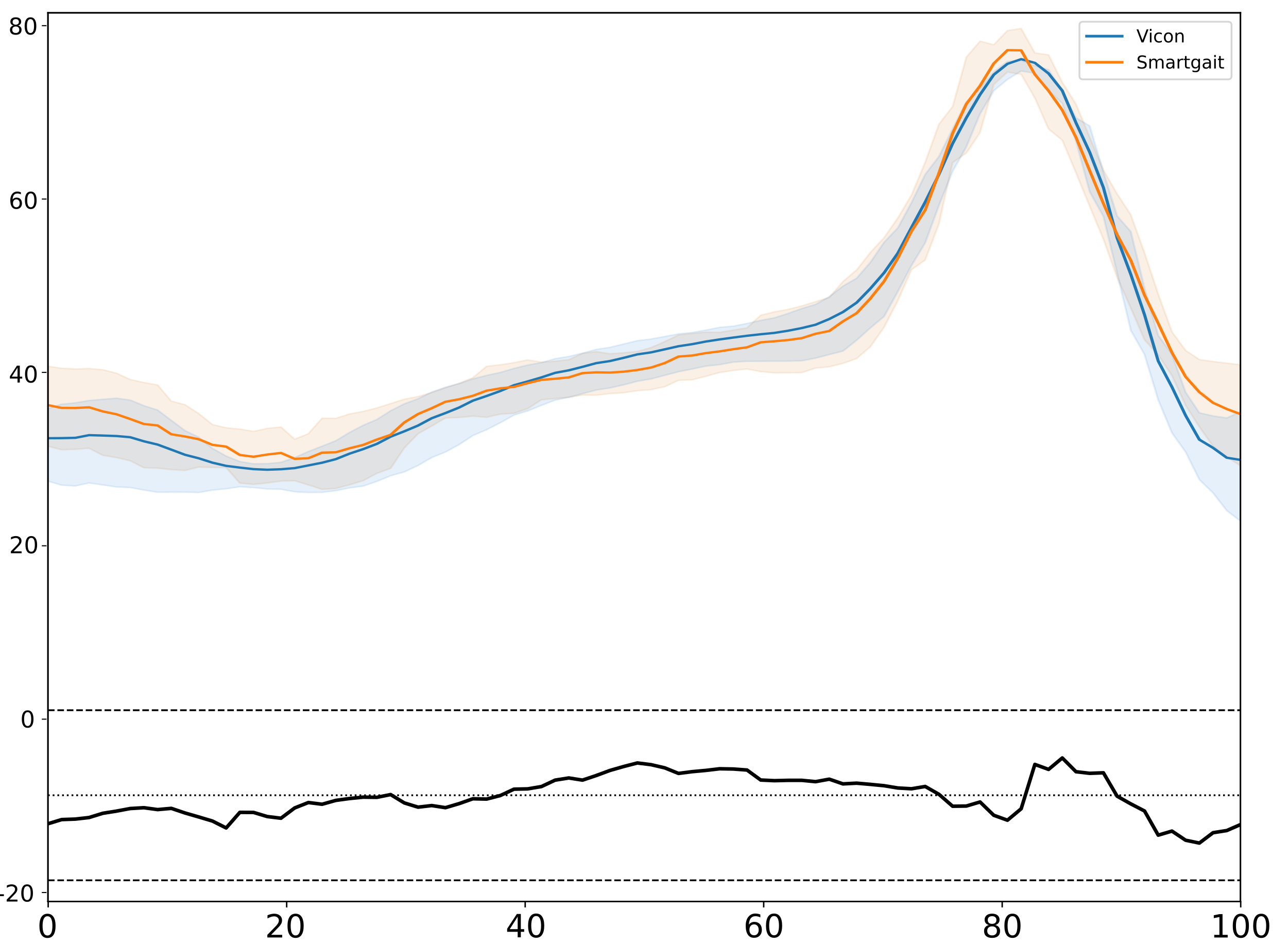

Supplement: Supplementary file 1 [file sensors-24-07819-s001.zip › spm_eval_FI11SI0801_sagital/FI11SI0801_angle_(2, 5, 5, 8)4.csv_plot_spm_fixed.png]

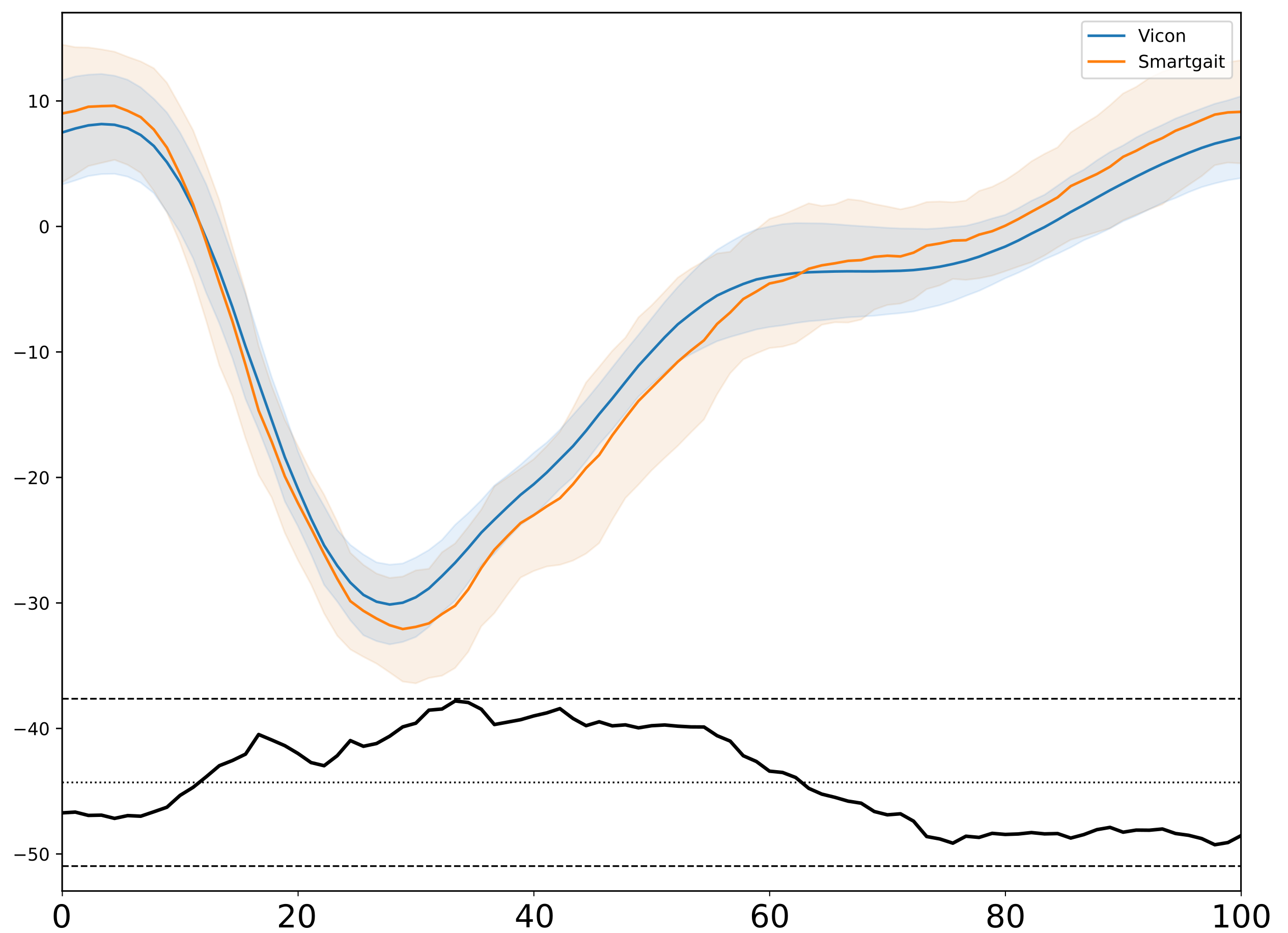

Supplement: Supplementary file 1 [file sensors-24-07819-s001.zip › spm_eval_FI11SI0801_sagital/FI11SI0801_angle_(2, 5, 12, 0)5.csv_plot_spm_fixed.png]

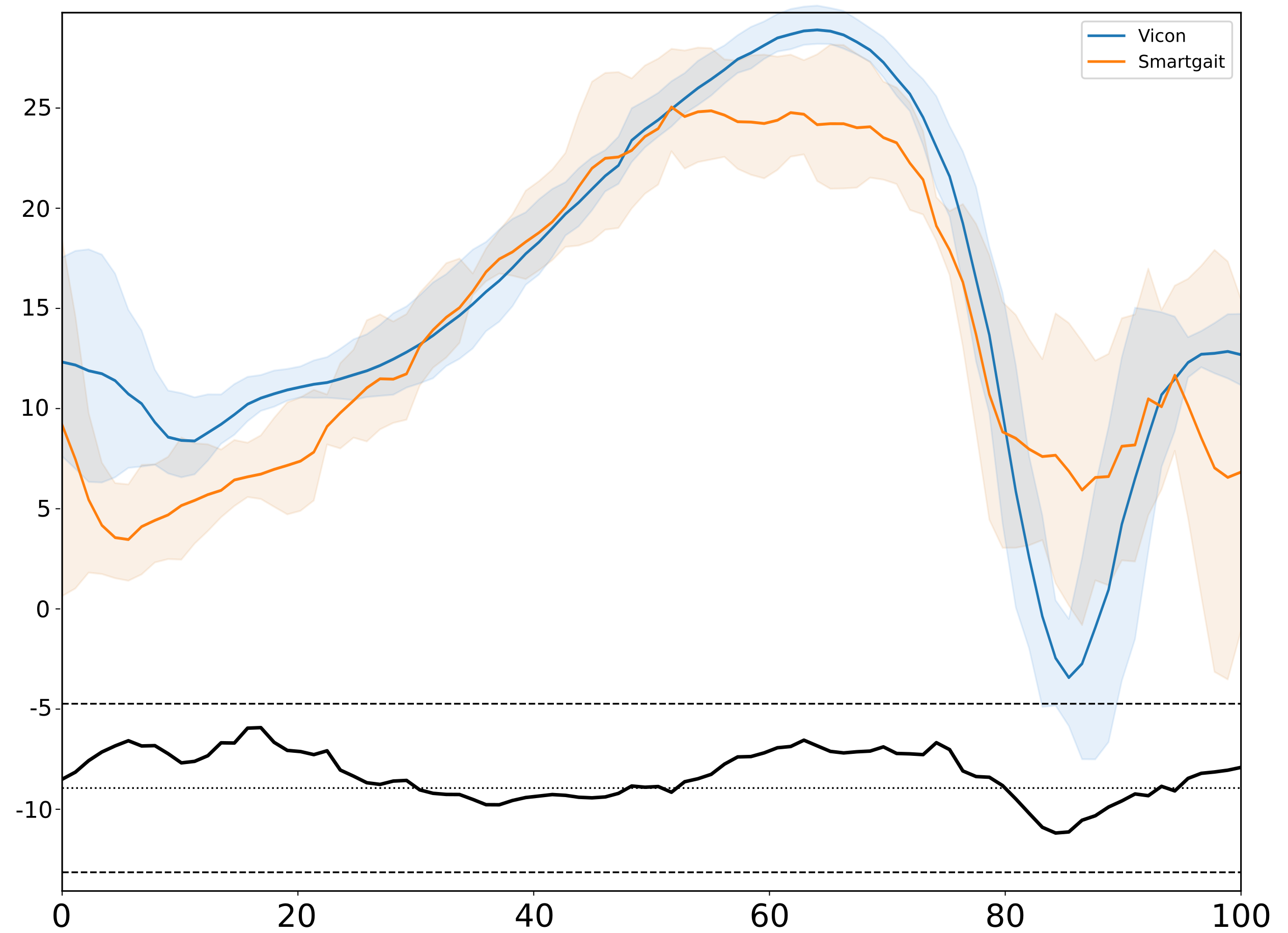

Supplement: Supplementary file 1 [file sensors-24-07819-s001.zip › spm_eval_FI11SI0801_sagital/FI11SI0801_angle_(5, 8, 8, 11)1.csv_plot_spm_fixed.png]

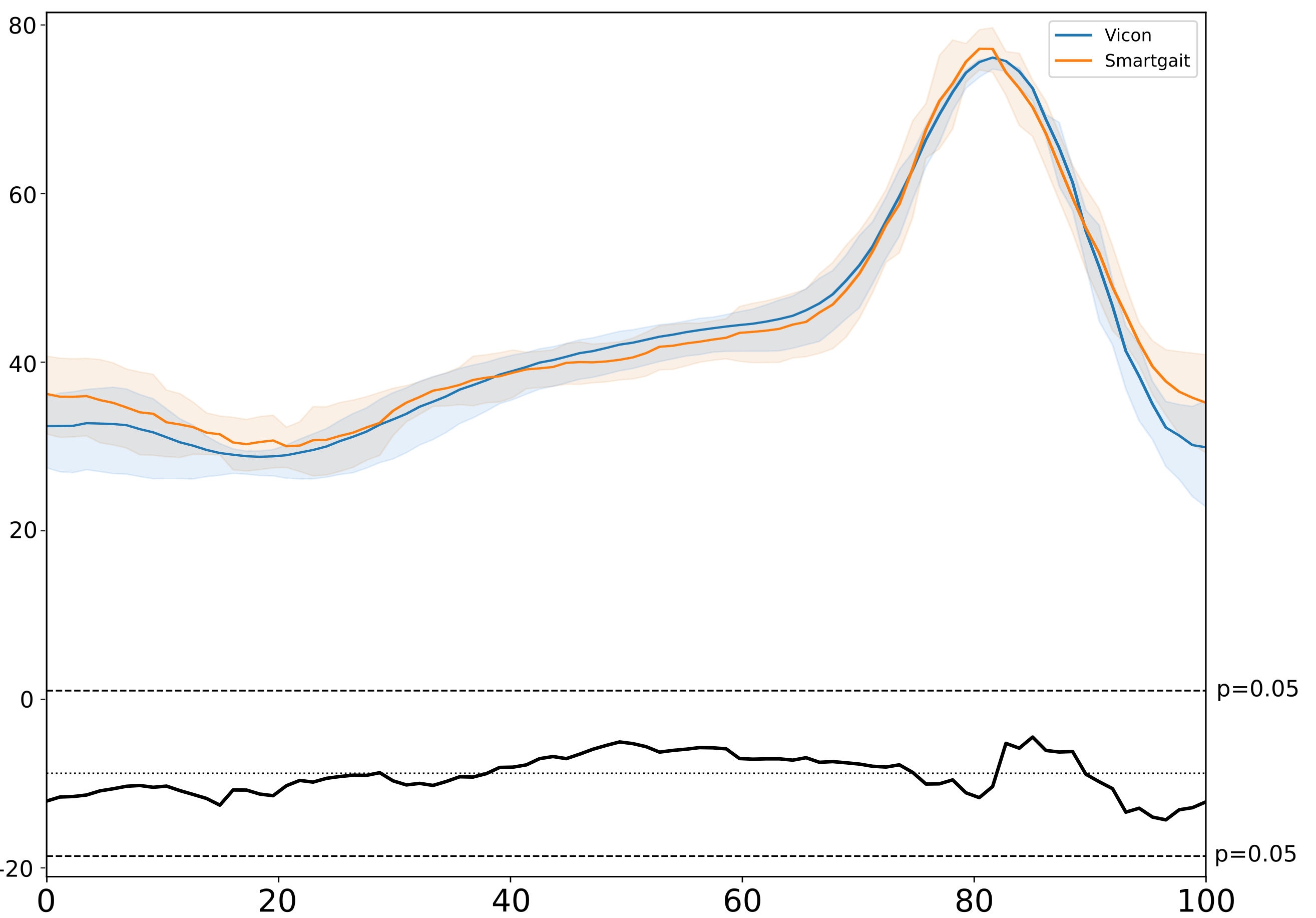

Supplement: Supplementary file 1 [file sensors-24-07819-s001.zip › spm_eval_FI11SI0801_sagital/FI11SI0801_angle_(2, 5, 5, 8)4.csv_plot_spm_fixed_.png]

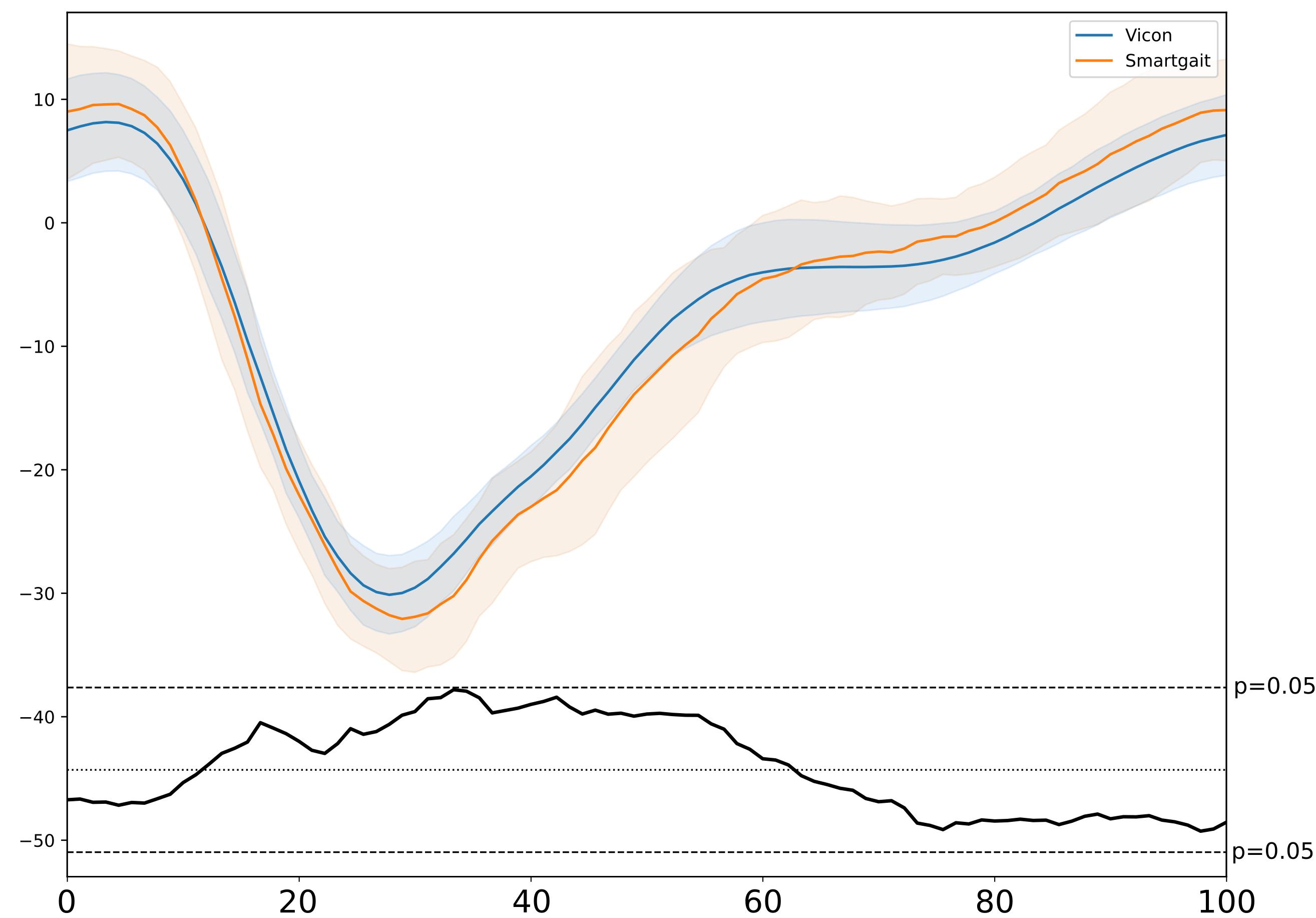

Supplement: Supplementary file 1 [file sensors-24-07819-s001.zip › spm_eval_FI11SI0801_sagital/FI11SI0801_angle_(2, 5, 12, 0)5.csv_plot_spm_fixed_.png]

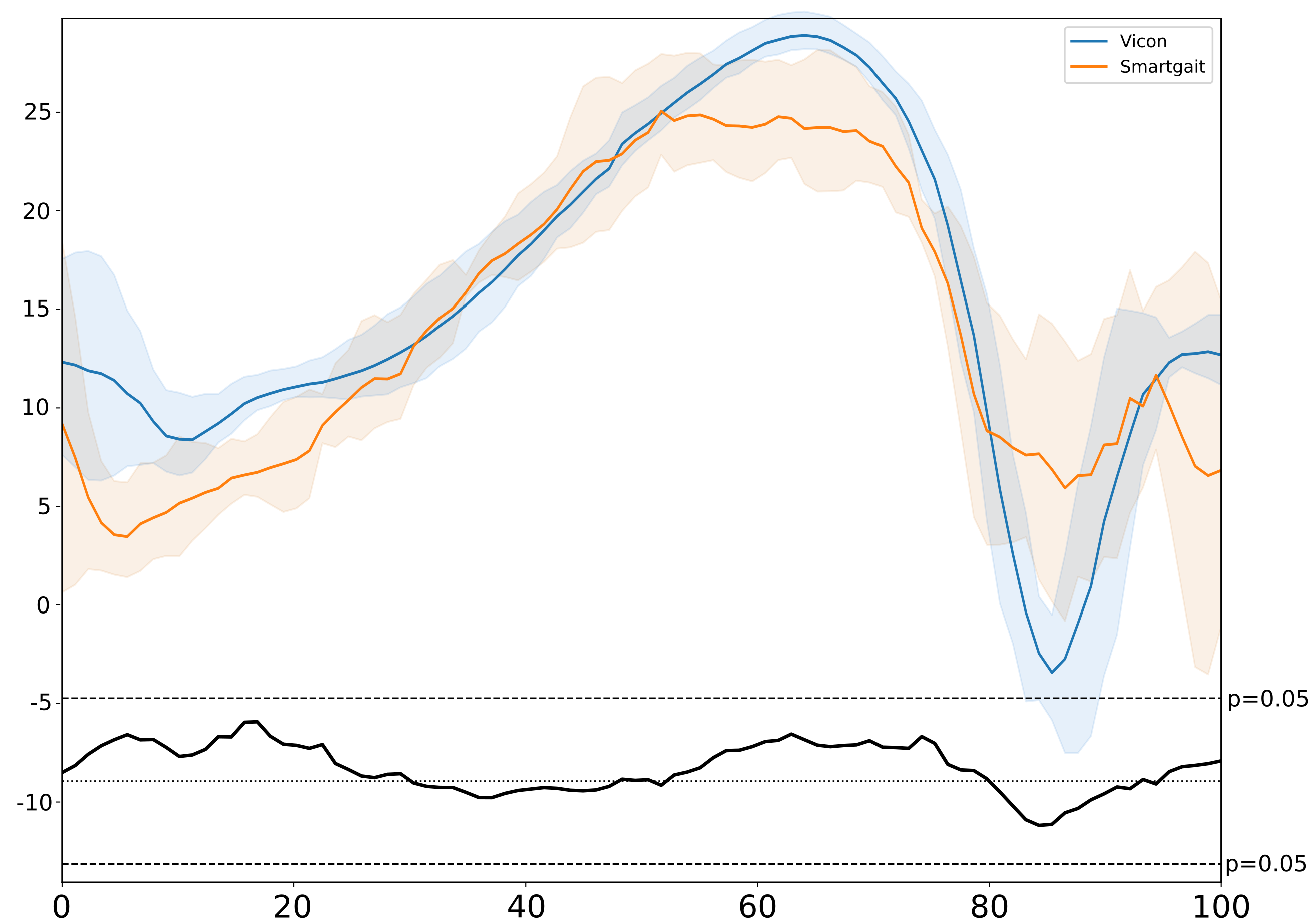

Supplement: Supplementary file 1 [file sensors-24-07819-s001.zip › spm_eval_FI11SI0801_sagital/FI11SI0801_angle_(5, 8, 8, 11)1.csv_plot_spm_fixed_.png]

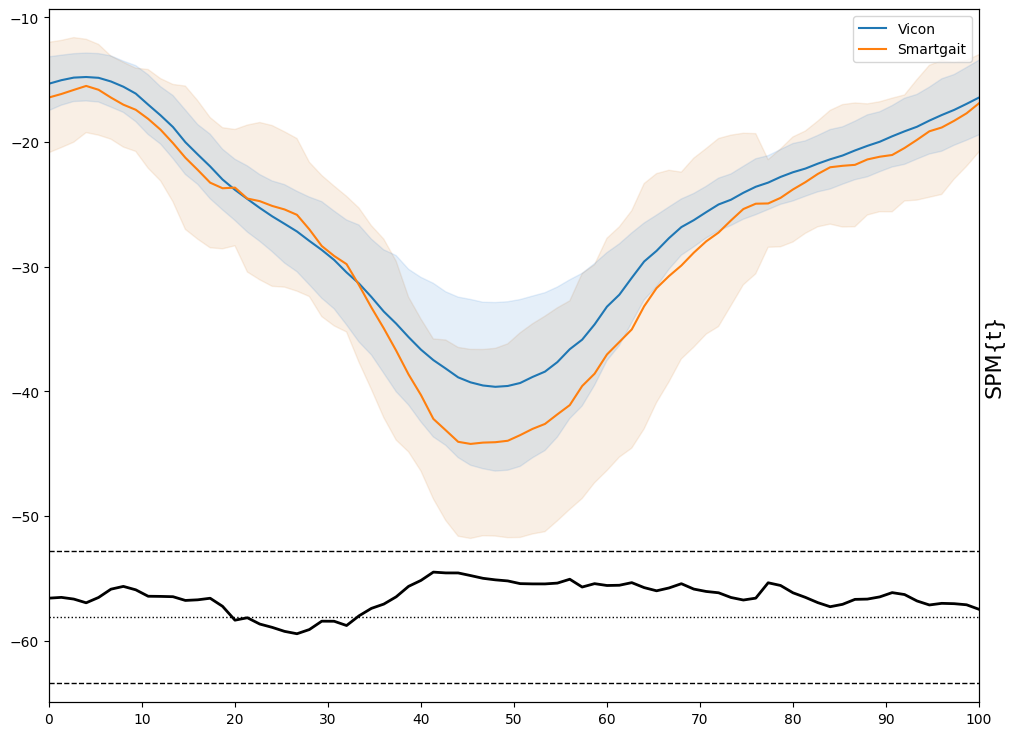

Supplement: Supplementary file 1 [file sensors-24-07819-s001.zip › spm_eval_HE20ÜB06_frontal/HE20ÜB06_angle_(2, 5, 12, 0)3.csv_plot_spm.png]

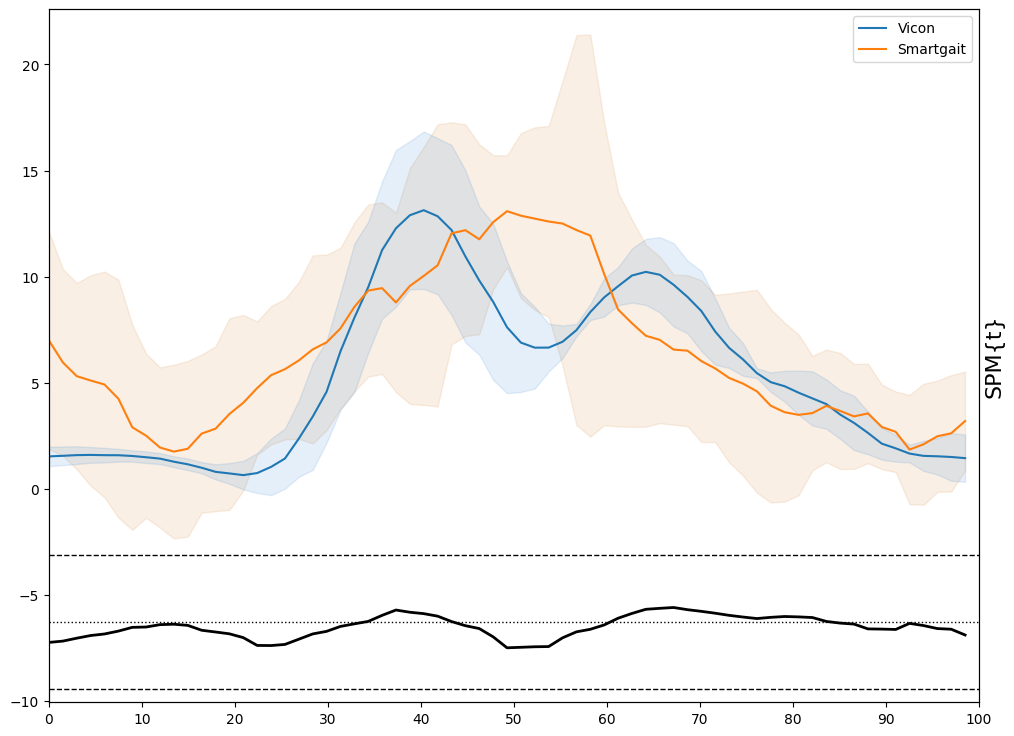

Supplement: Supplementary file 1 [file sensors-24-07819-s001.zip › spm_eval_HE20ÜB06_frontal/HE20ÜB06_angle_(2, 5, 5, 8)3.csv_plot_spm.png]

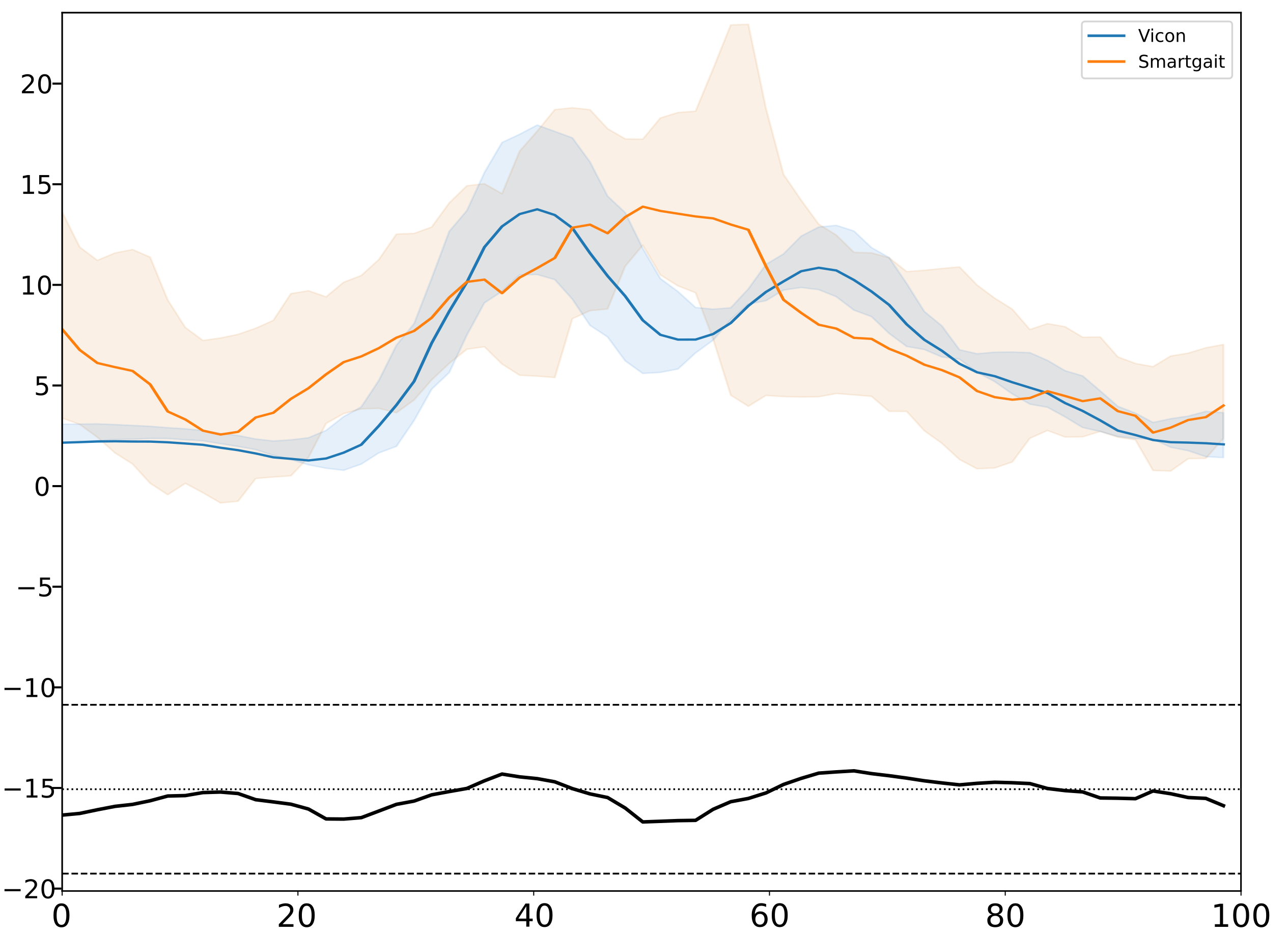

Supplement: Supplementary file 1 [file sensors-24-07819-s001.zip › spm_eval_HE20ÜB06_frontal/HE20ÜB06_angle_(2, 5, 5, 8)3.csv_plot_spm_fixed.png]

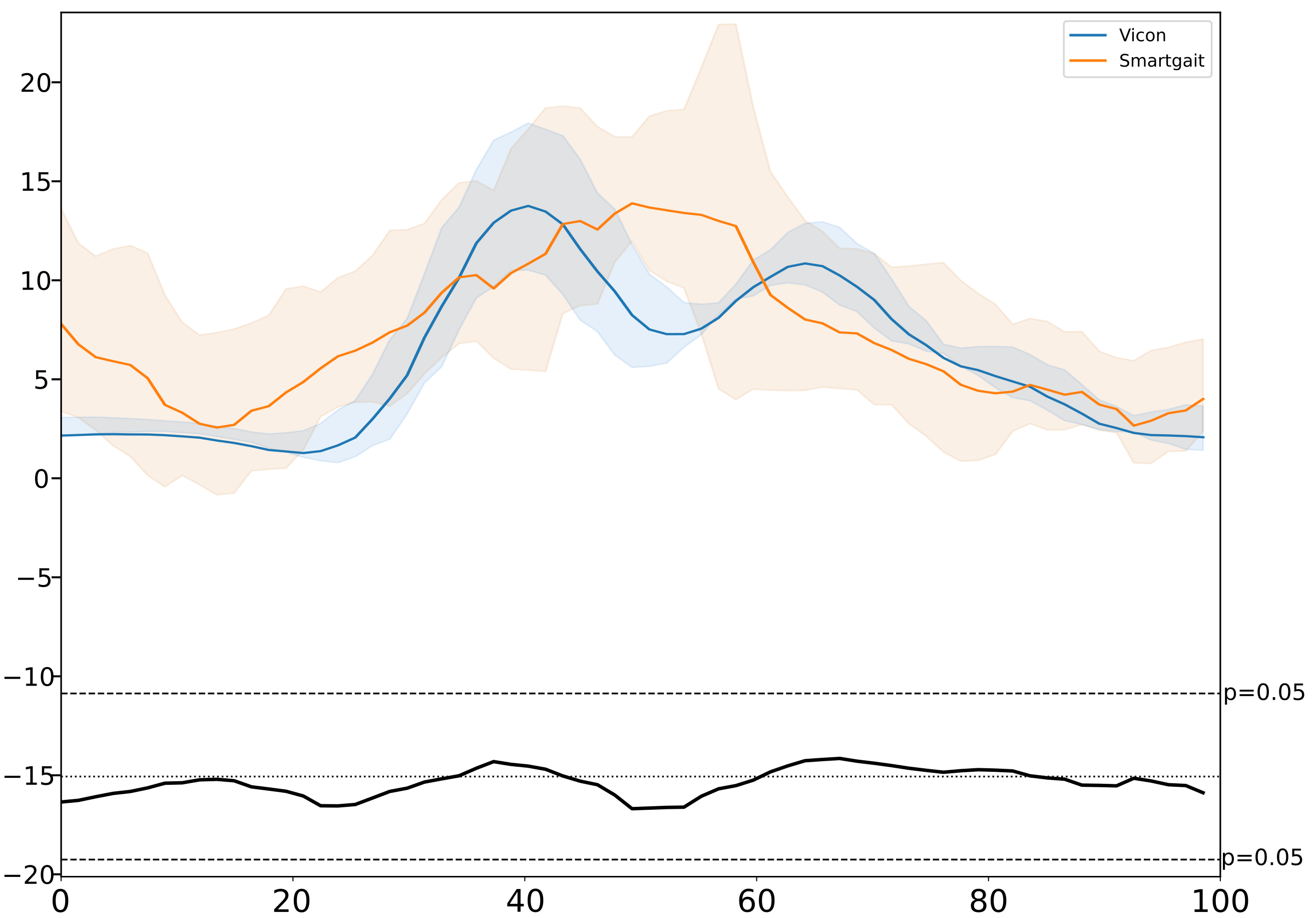

Supplement: Supplementary file 1 [file sensors-24-07819-s001.zip › spm_eval_HE20ÜB06_frontal/HE20ÜB06_angle_(2, 5, 5, 8)3.csv_plot_spm_fixed_.png]

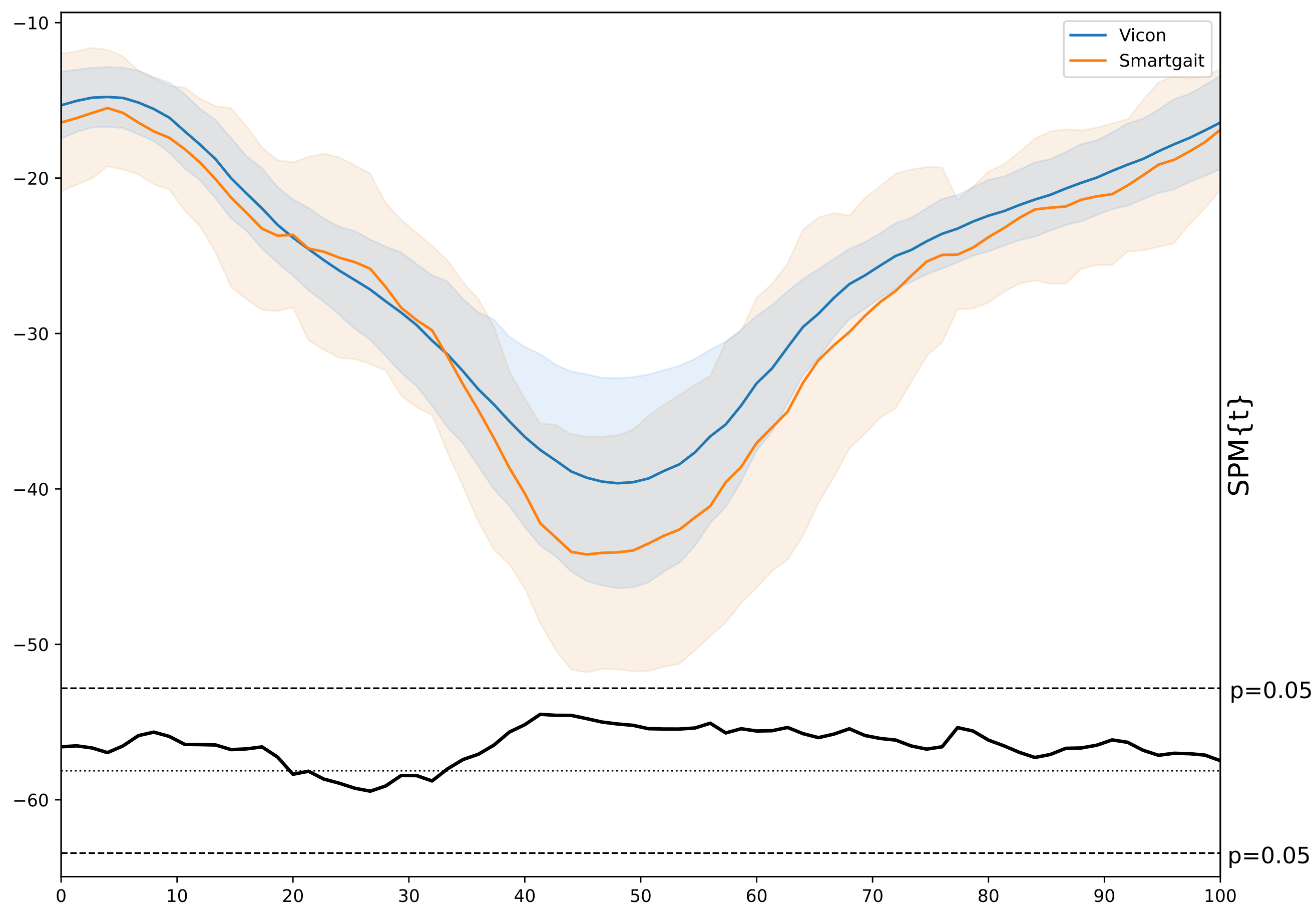

Supplement: Supplementary file 1 [file sensors-24-07819-s001.zip › spm_eval_HE20ÜB06_frontal/HE20ÜB06_angle_(2, 5, 12, 0)3.csv_plot_spm_fixed_.png]

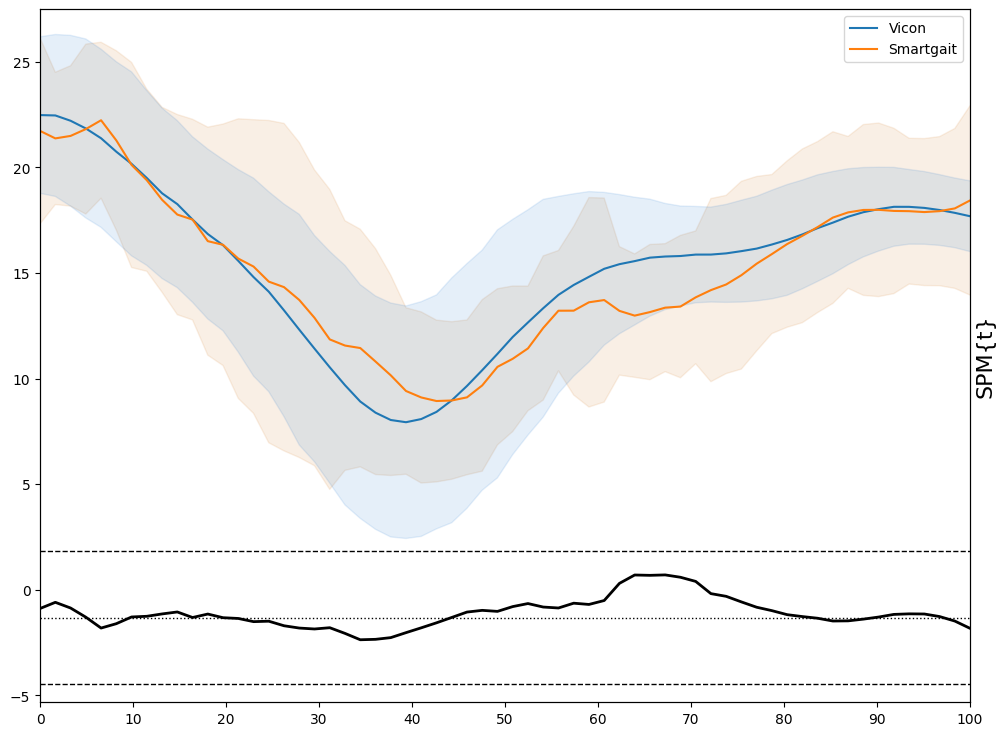

Supplement: Supplementary file 1 [file sensors-24-07819-s001.zip › spm_eval_HE20ÜB06_sagital/HE20ÜB06_angle_(2, 5, 12, 0)2.csv_plot_spm.png]

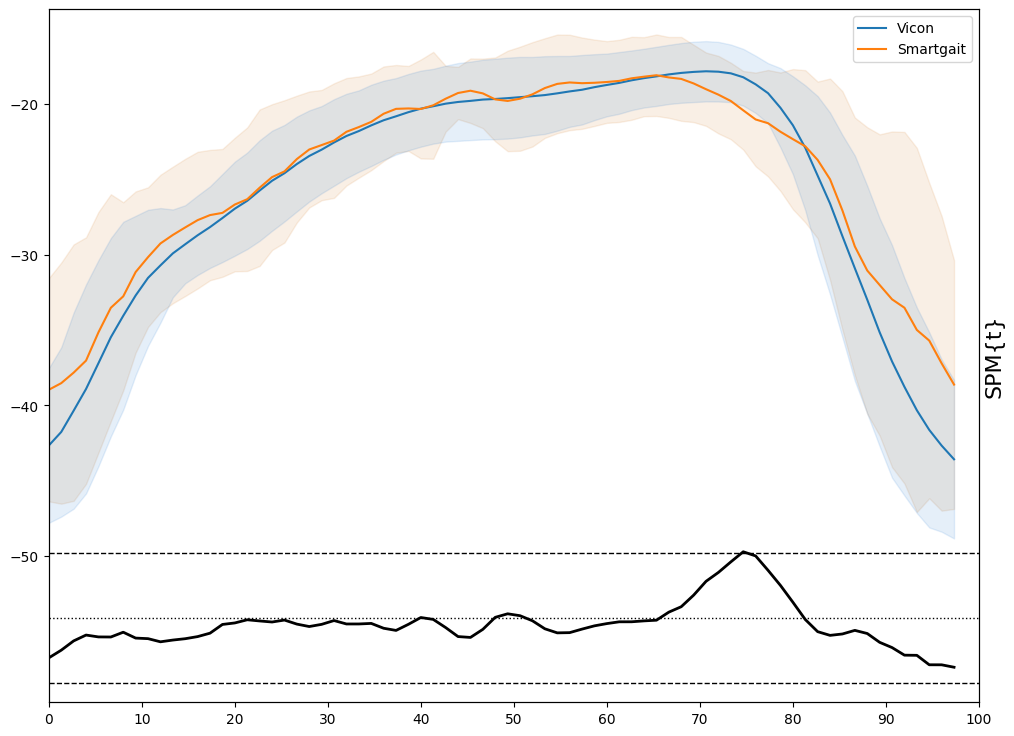

Supplement: Supplementary file 1 [file sensors-24-07819-s001.zip › spm_eval_HE20ÜB06_sagital/HE20ÜB06_angle_(0, 4, 4, 7)2.csv_plot_spm_.png]

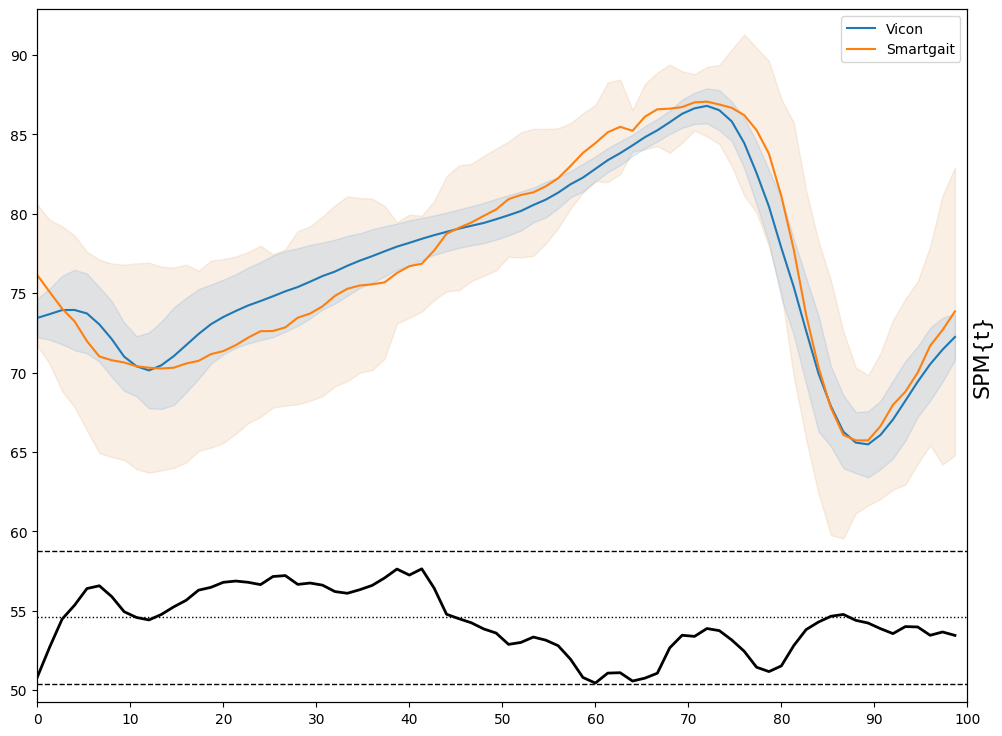

Supplement: Supplementary file 1 [file sensors-24-07819-s001.zip › spm_eval_HE20ÜB06_sagital/HE20ÜB06_angle_(4, 7, 7, 10)3.csv_plot_spm.png]

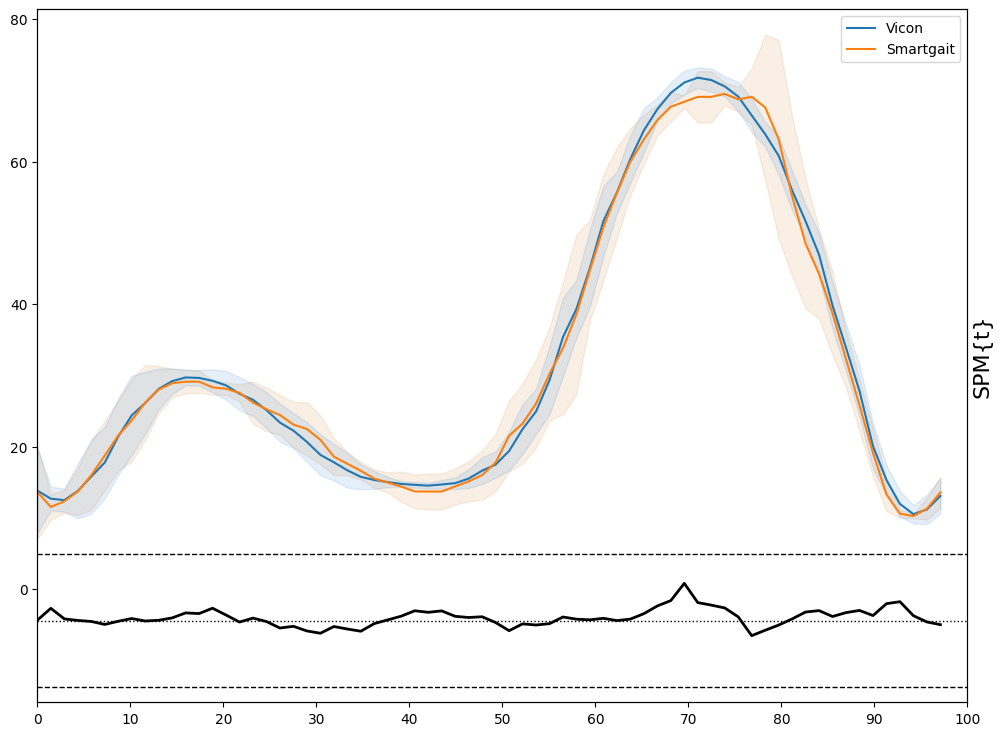

Supplement: Supplementary file 1 [file sensors-24-07819-s001.zip › spm_eval_HE20ÜB06_sagital/HE20ÜB06_angle_(2, 5, 5, 8)1.csv_plot_spm.png]

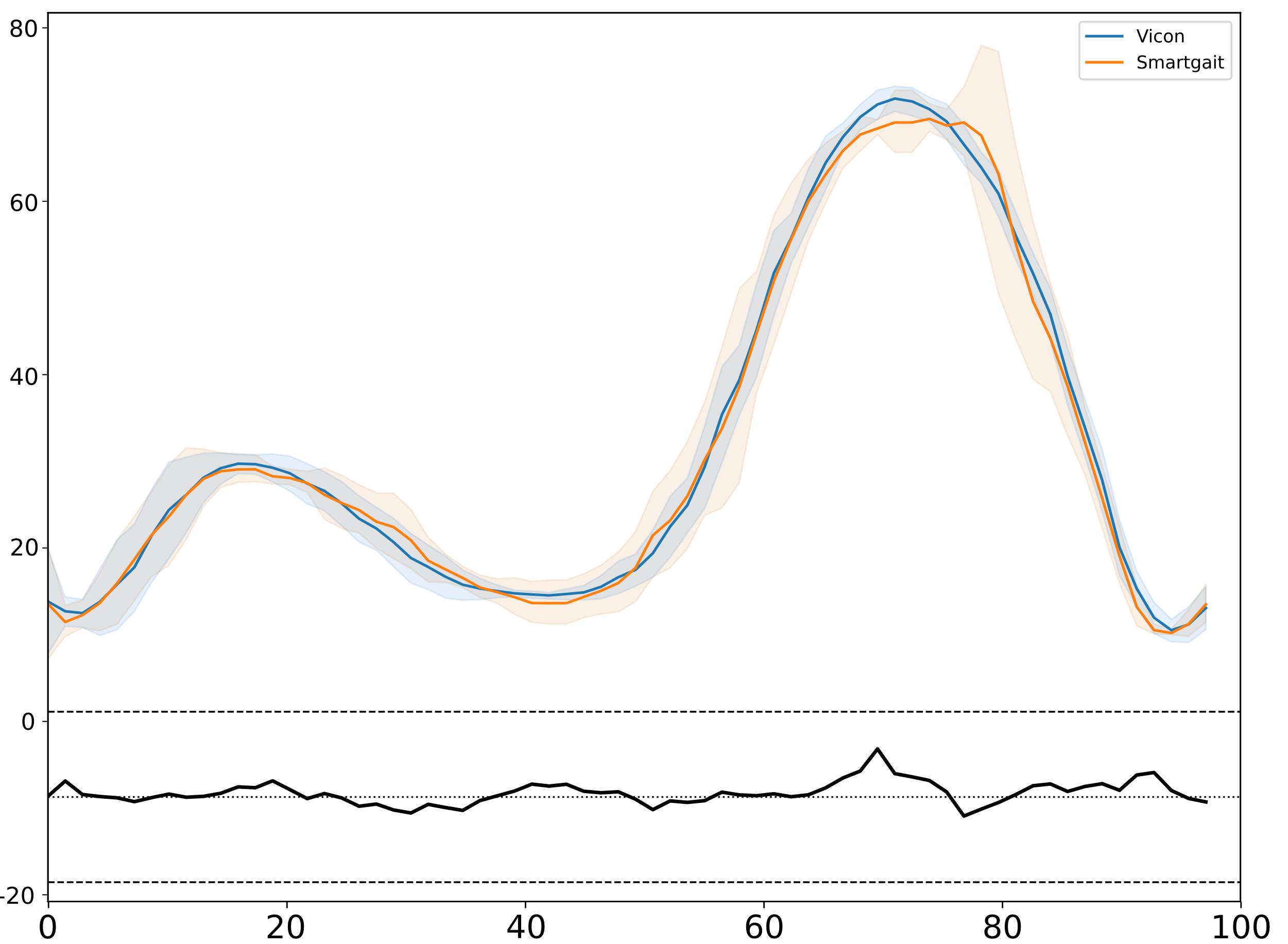

Supplement: Supplementary file 1 [file sensors-24-07819-s001.zip › spm_eval_HE20ÜB06_sagital/HE20ÜB06_angle_(2, 5, 5, 8)1.csv_plot_spm_fixed.png]

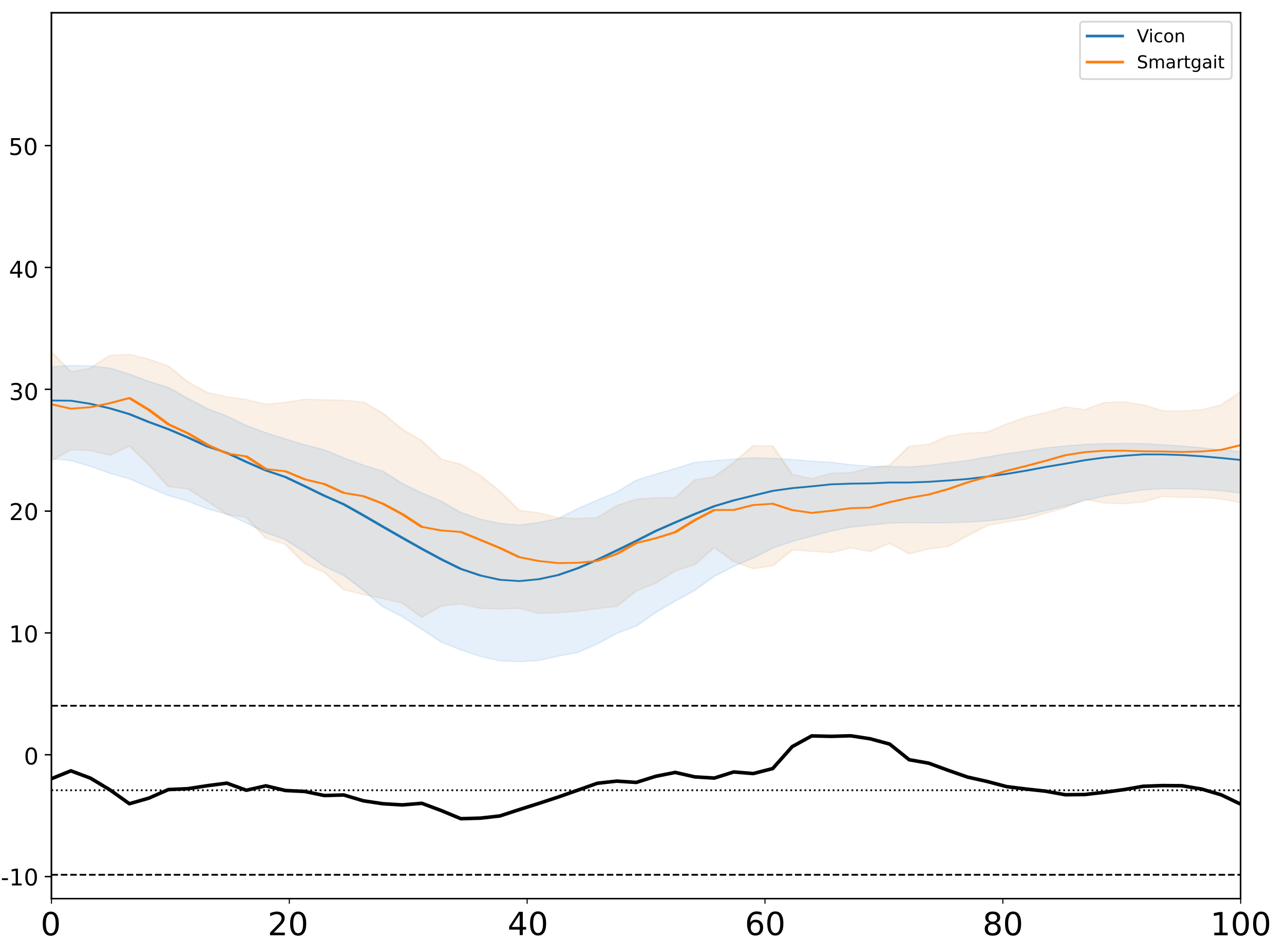

Supplement: Supplementary file 1 [file sensors-24-07819-s001.zip › spm_eval_HE20ÜB06_sagital/HE20ÜB06_angle_(2, 5, 12, 0)2.csv_plot_spm_fixed.png]

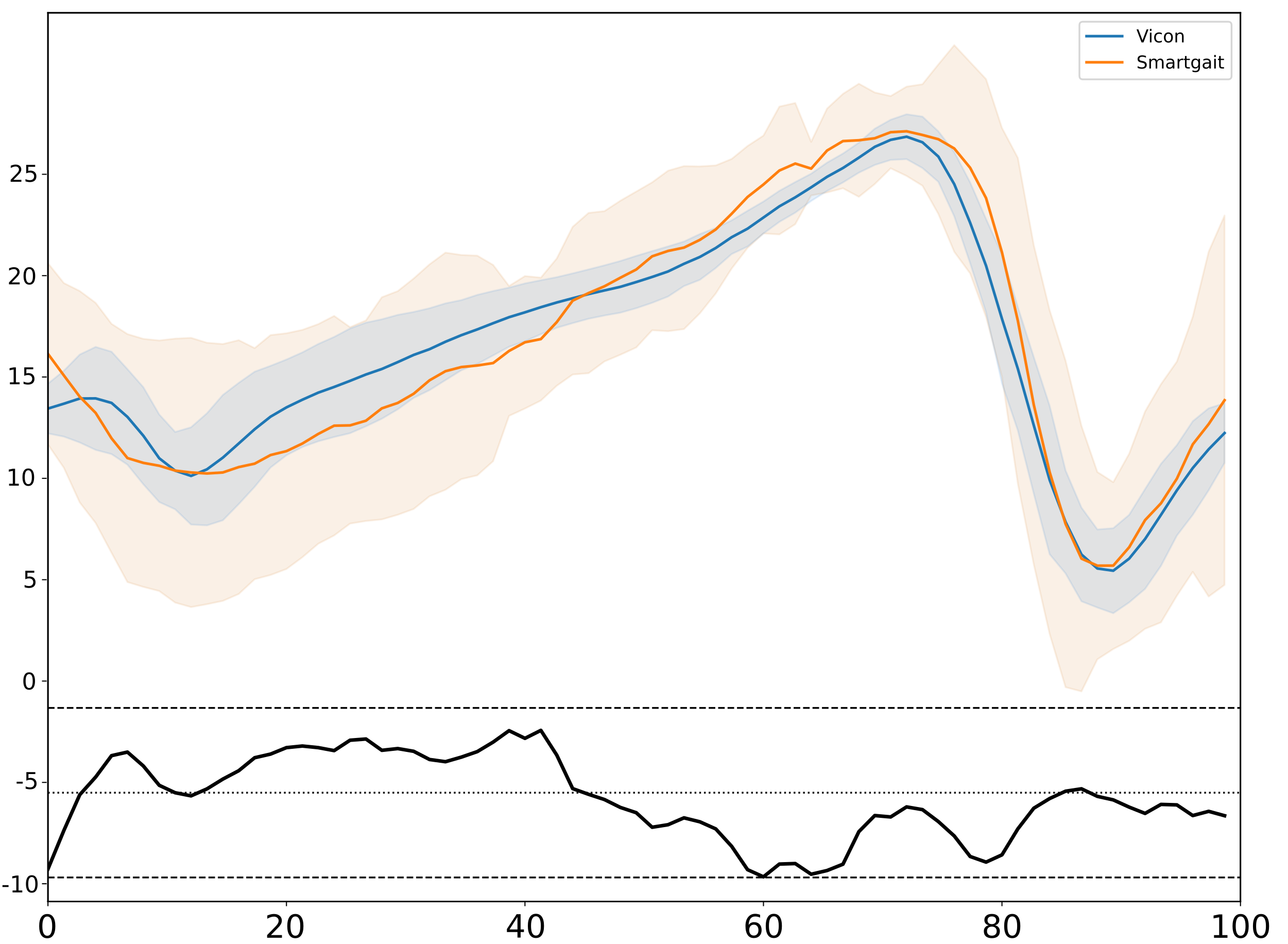

Supplement: Supplementary file 1 [file sensors-24-07819-s001.zip › spm_eval_HE20ÜB06_sagital/HE20ÜB06_angle_(5, 8, 8, 11)3.csv_plot_spm_fixed.png]

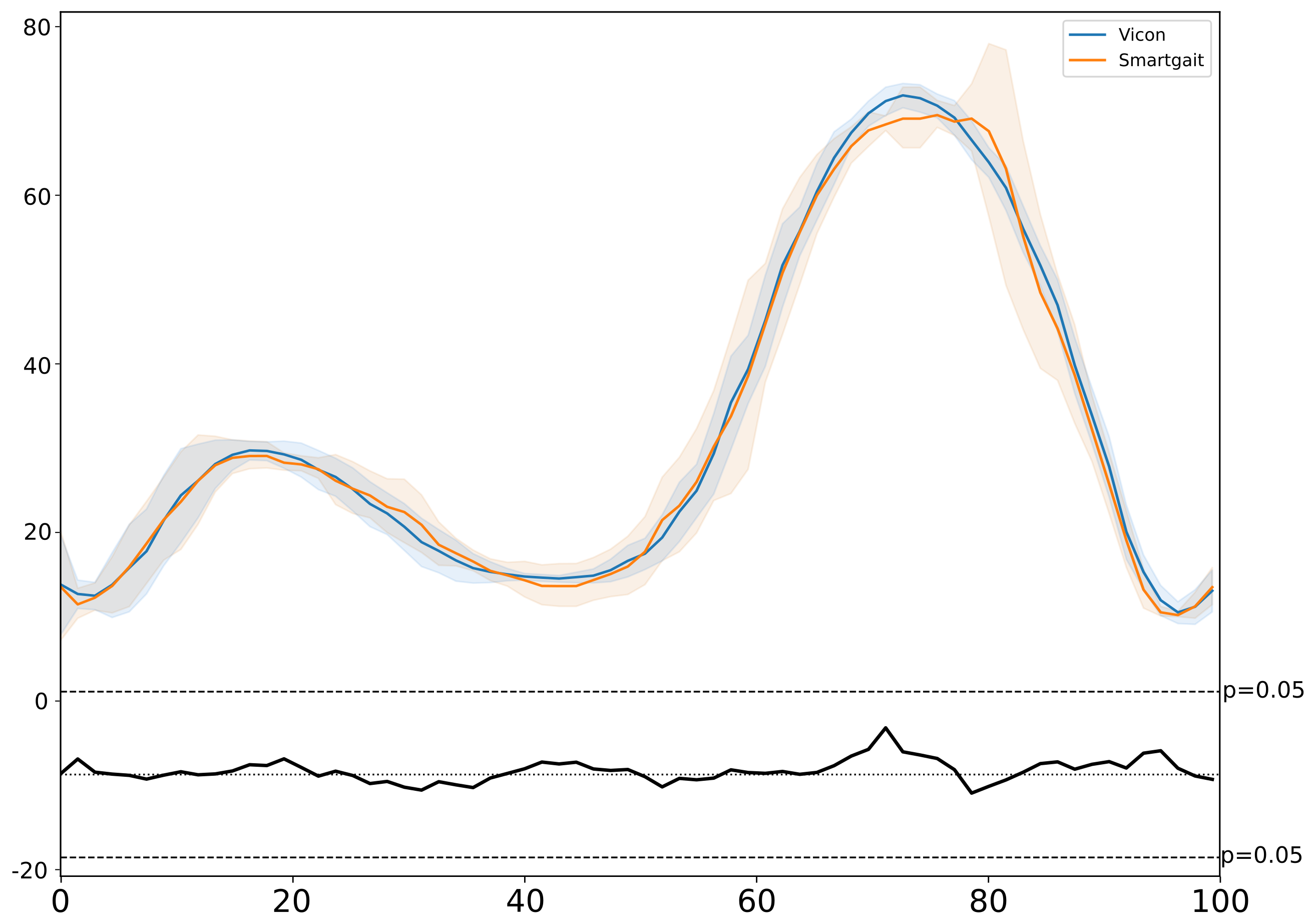

Supplement: Supplementary file 1 [file sensors-24-07819-s001.zip › spm_eval_HE20ÜB06_sagital/HE20ÜB06_angle_(2, 5, 5, 8)1.csv_plot_spm_fixed_.png]

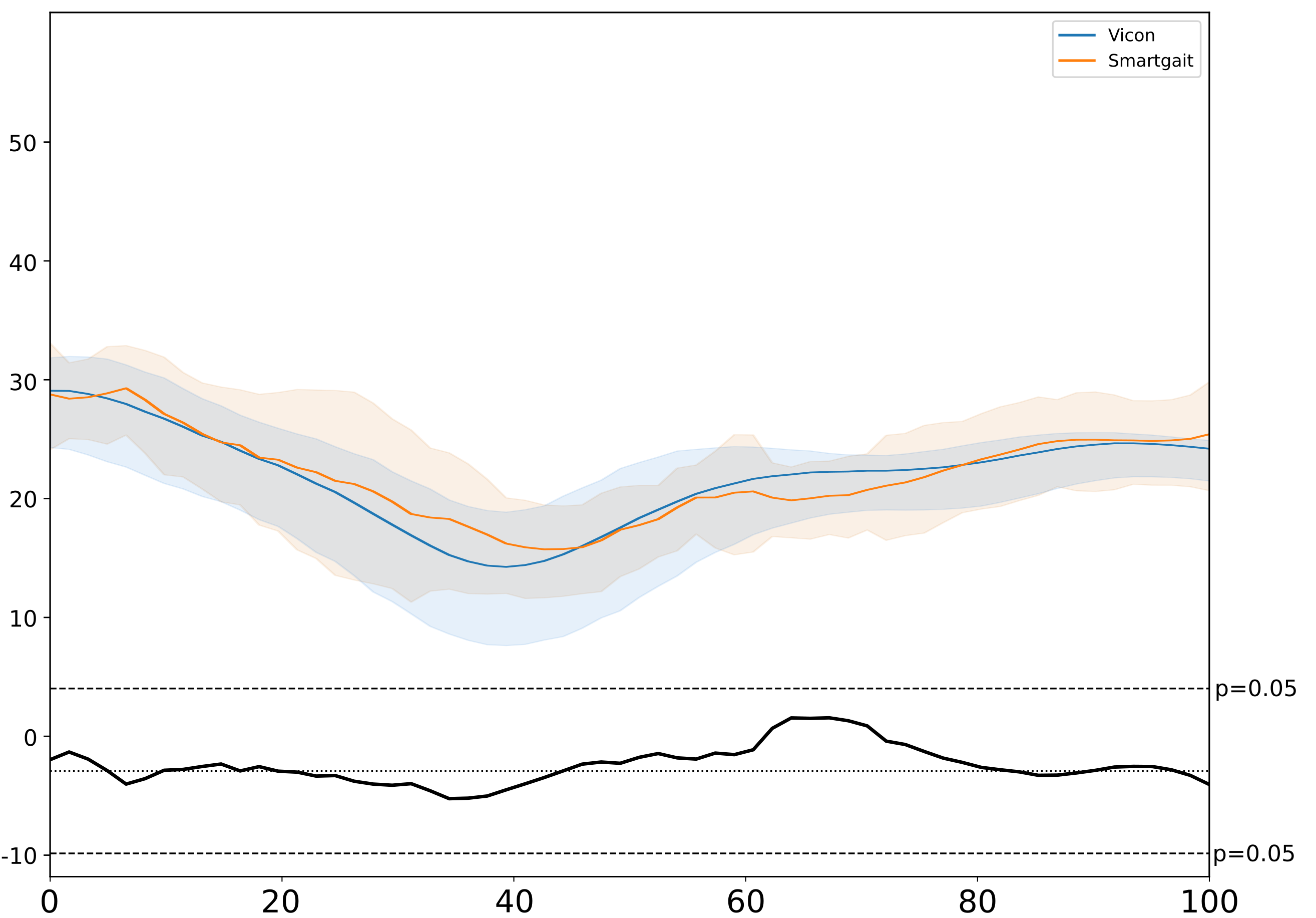

Supplement: Supplementary file 1 [file sensors-24-07819-s001.zip › spm_eval_HE20ÜB06_sagital/HE20ÜB06_angle_(2, 5, 12, 0)2.csv_plot_spm_fixed_.png]

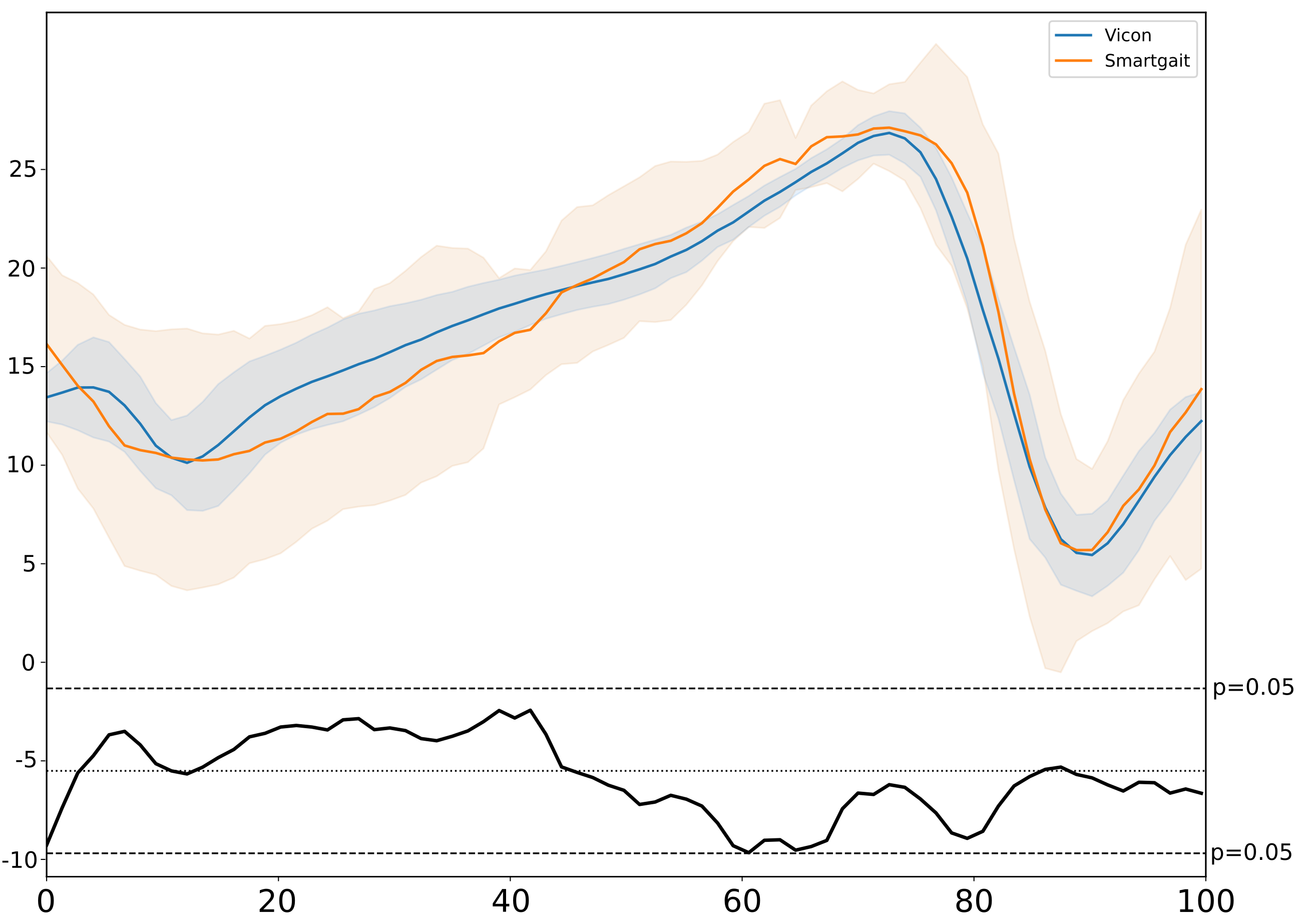

Supplement: Supplementary file 1 [file sensors-24-07819-s001.zip › spm_eval_HE20ÜB06_sagital/HE20ÜB06_angle_(4, 7, 7, 10)3.csv_plot_spm_fixed_.png]

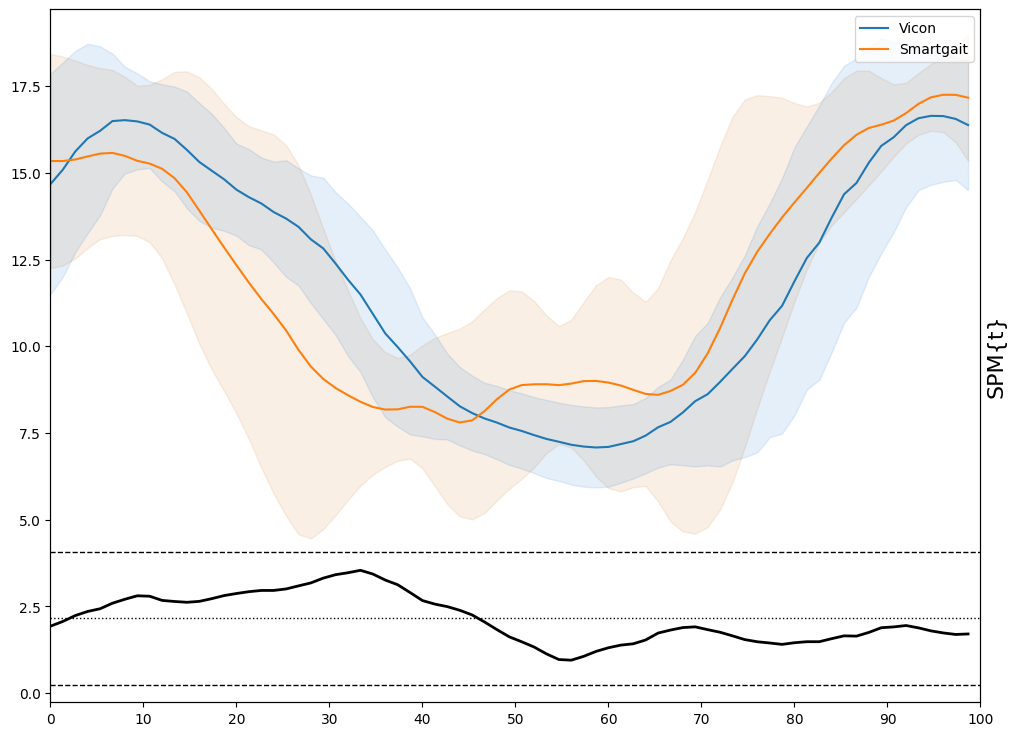

Supplement: Supplementary file 1 [file sensors-24-07819-s001.zip › spm_eval_LA13RE28_frontal/LA13RE28_angle_(2, 5, 12, 0)1.csv_plot_spm.png]

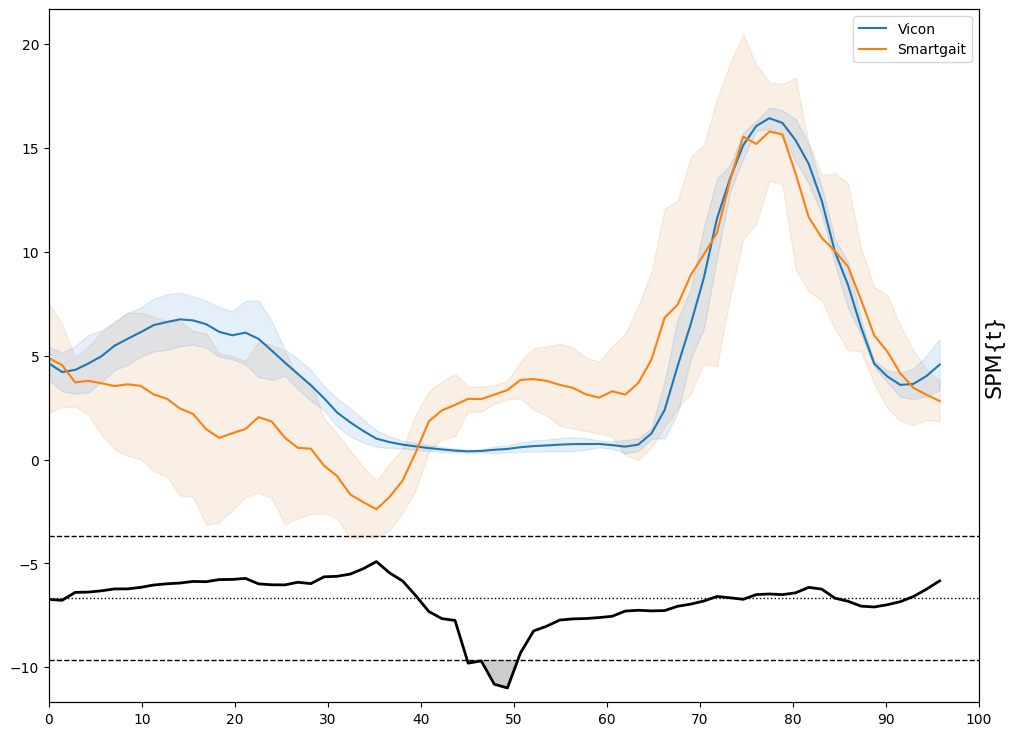

Supplement: Supplementary file 1 [file sensors-24-07819-s001.zip › spm_eval_LA13RE28_frontal/LA13RE28_angle_(2, 5, 5, 8)1.csv_plot_spm.png]

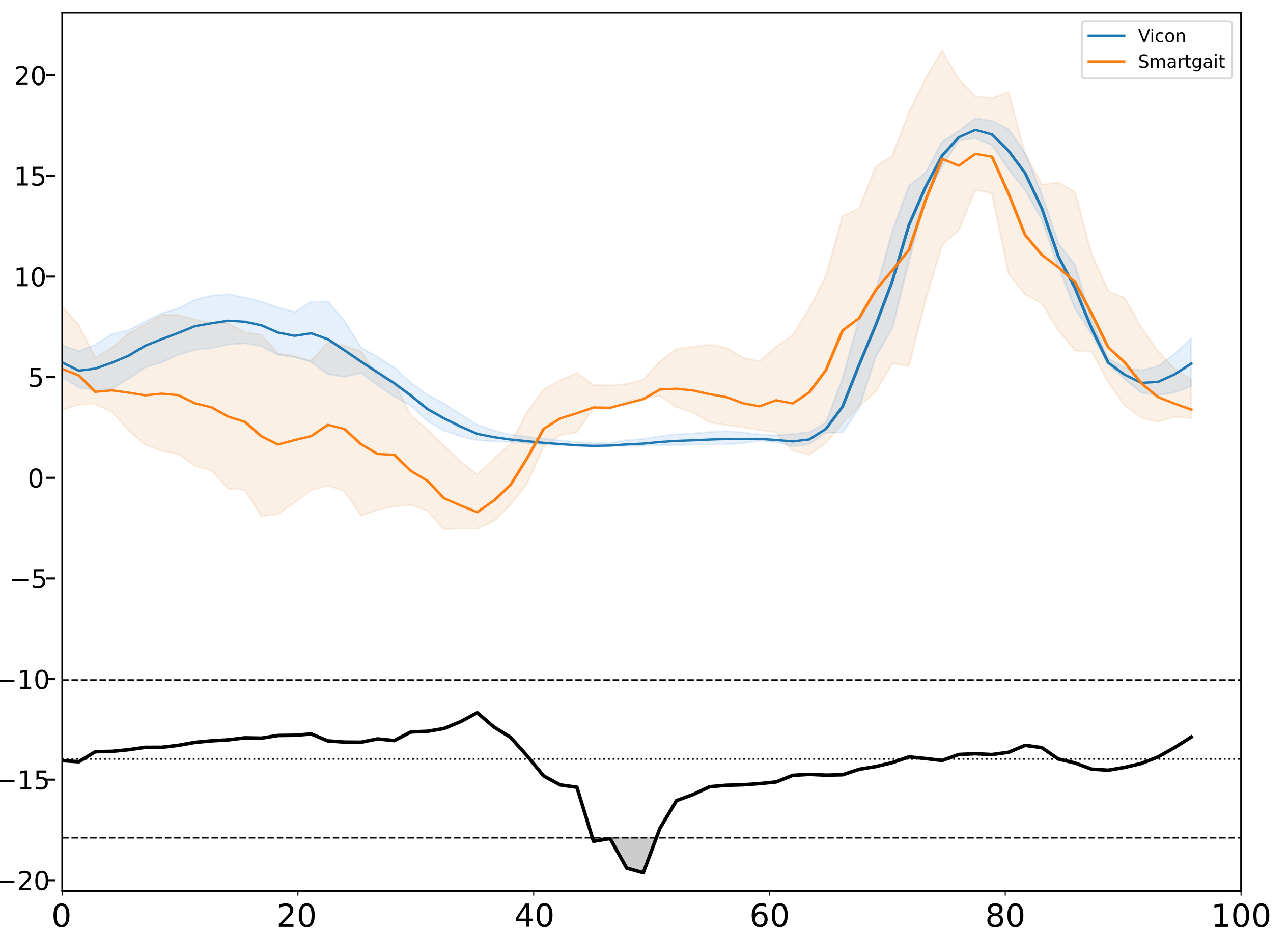

Supplement: Supplementary file 1 [file sensors-24-07819-s001.zip › spm_eval_LA13RE28_frontal/LA13RE28_angle_(2, 5, 5, 8)1.csv_plot_spm_fixed.png]

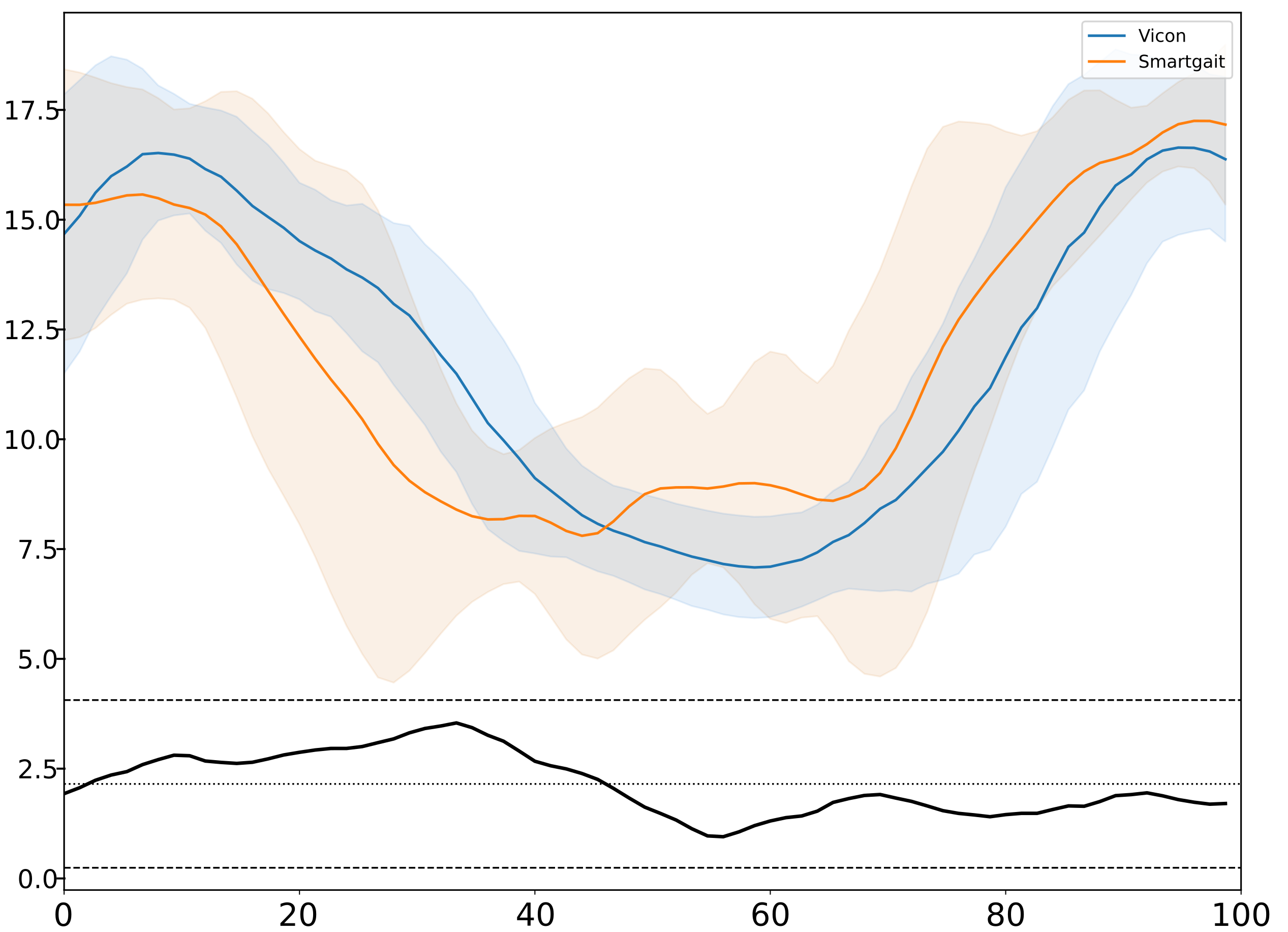

Supplement: Supplementary file 1 [file sensors-24-07819-s001.zip › spm_eval_LA13RE28_frontal/LA13RE28_angle_(2, 5, 12, 0)1.csv_plot_spm_fixed.png]

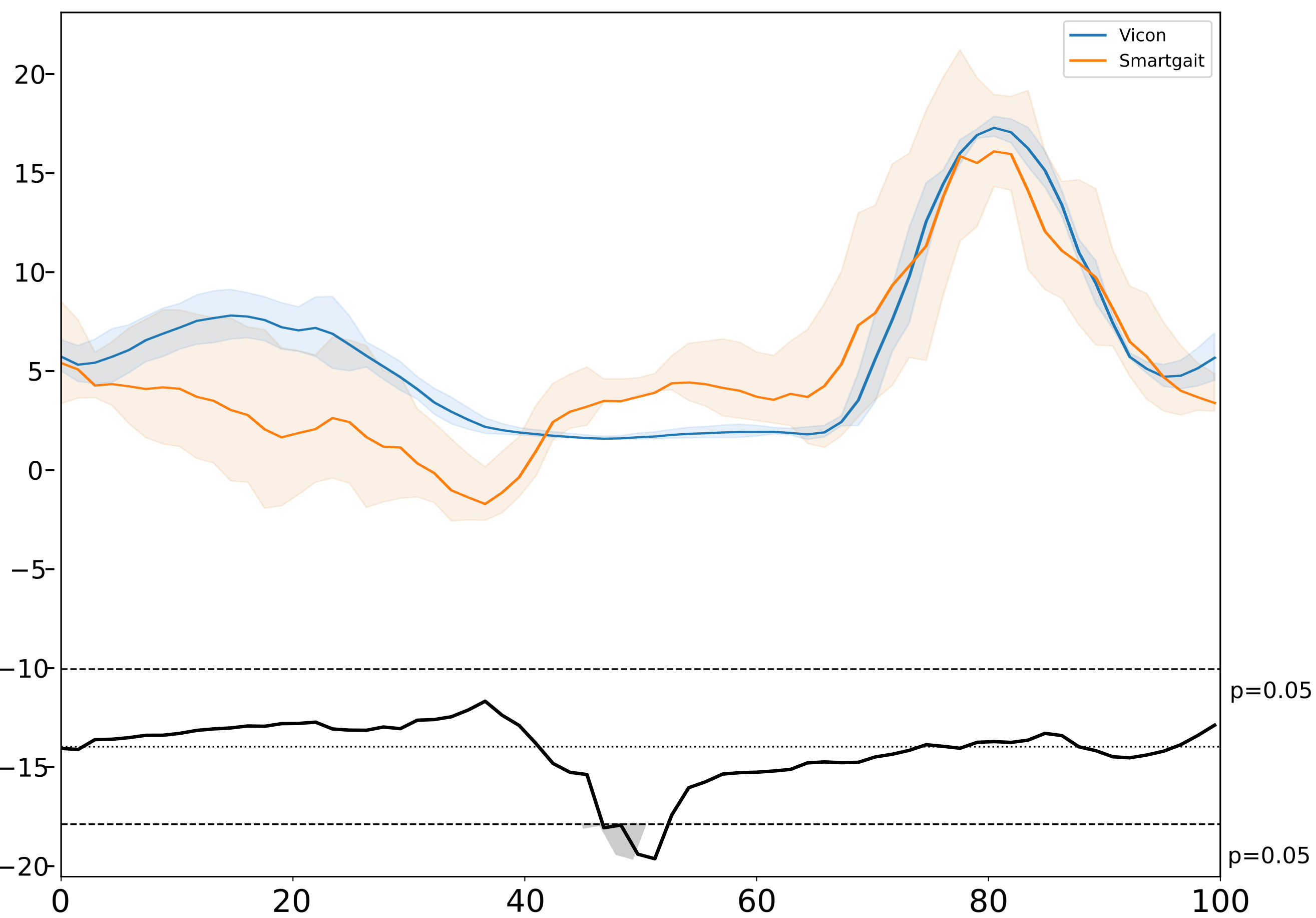

Supplement: Supplementary file 1 [file sensors-24-07819-s001.zip › spm_eval_LA13RE28_frontal/LA13RE28_angle_(2, 5, 5, 8)1.csv_plot_spm_fixed_.png]

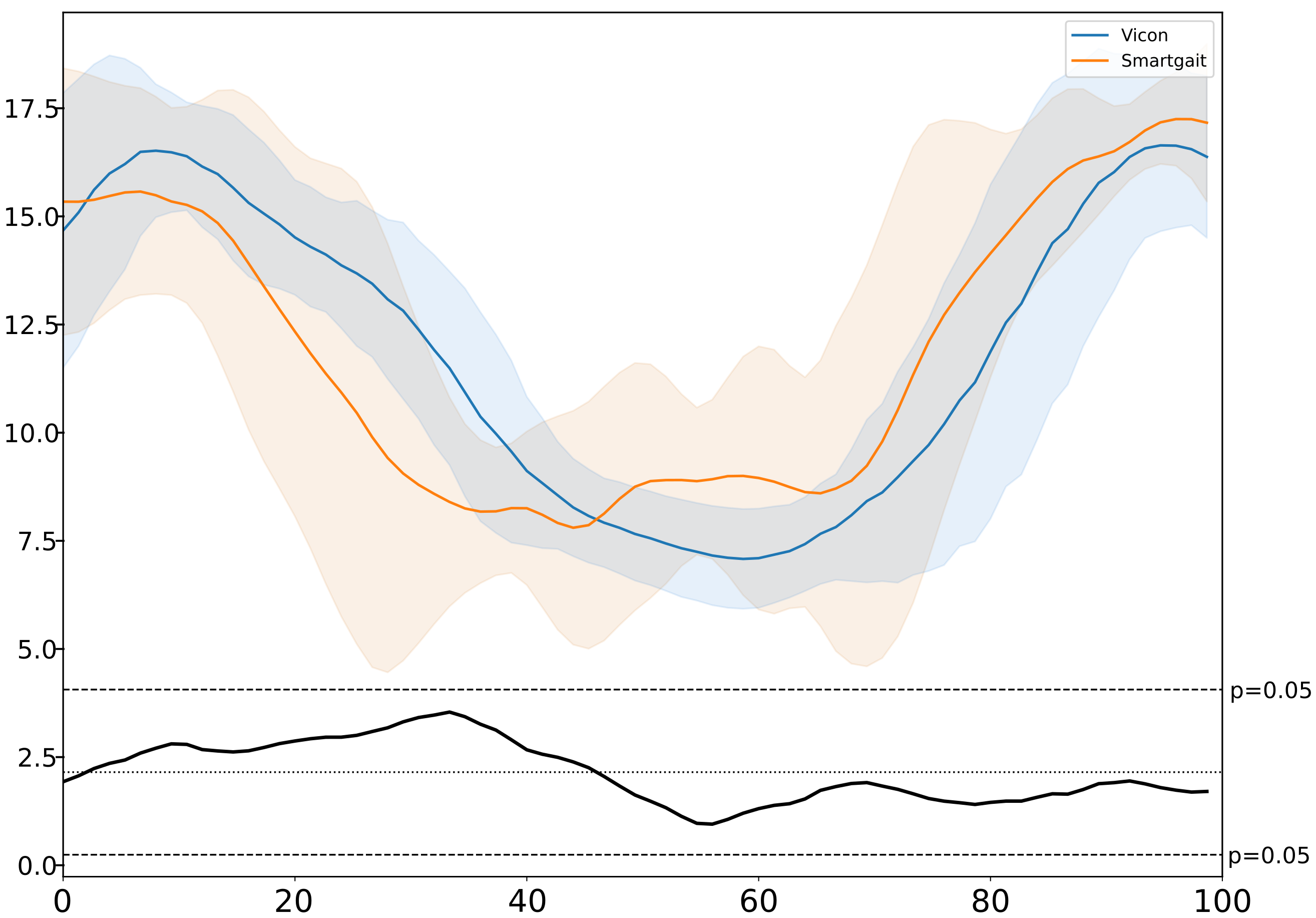

Supplement: Supplementary file 1 [file sensors-24-07819-s001.zip › spm_eval_LA13RE28_frontal/LA13RE28_angle_(2, 5, 12, 0)1.csv_plot_spm_fixed_.png]

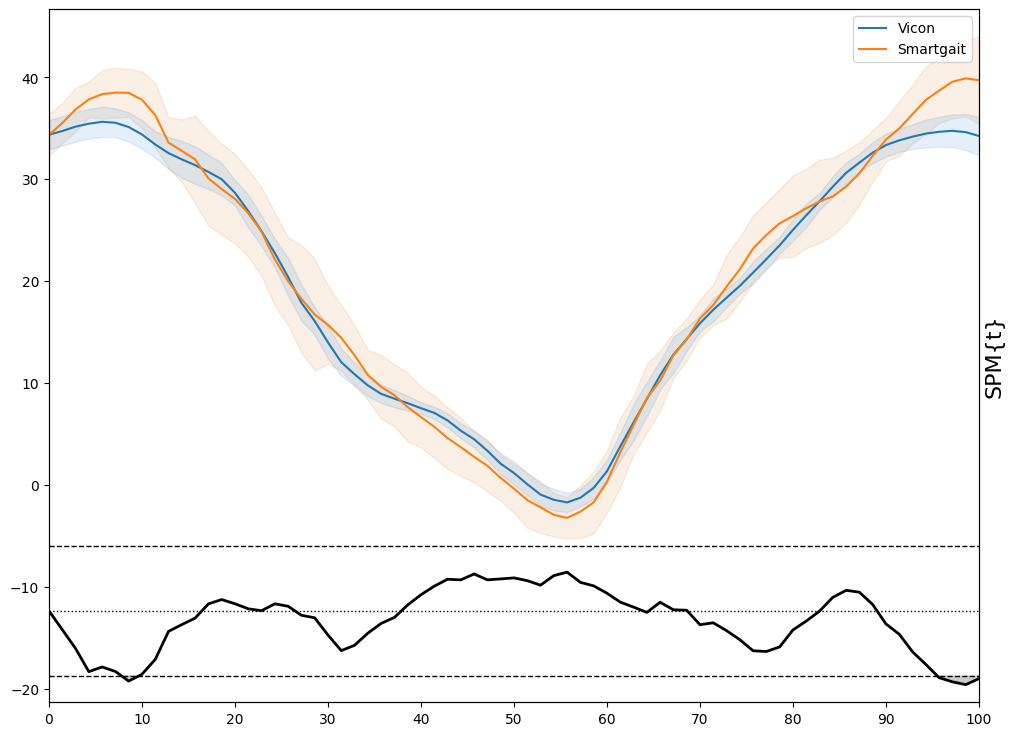

Supplement: Supplementary file 1 [file sensors-24-07819-s001.zip › spm_eval_LA13RE28_sagital/LA13RE28_angle_(2, 5, 12, 0)1.csv_plot_spm.png]

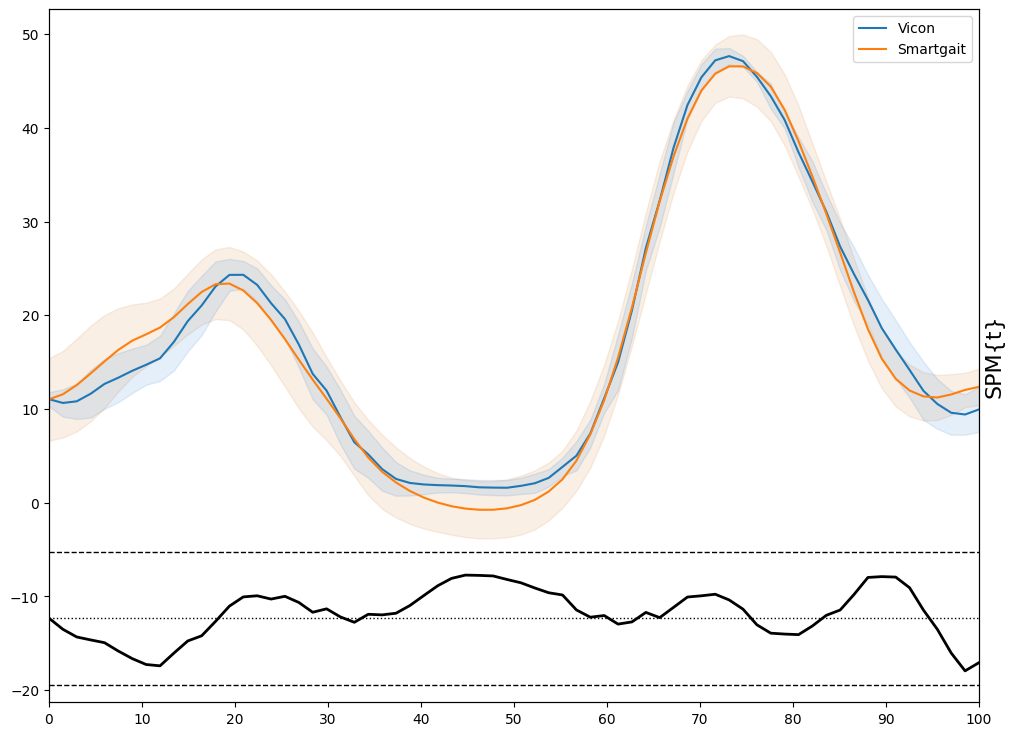

Supplement: Supplementary file 1 [file sensors-24-07819-s001.zip › spm_eval_LA13RE28_sagital/LA13RE28_angle_(2, 5, 5, 8)1.csv_plot_spm.png]

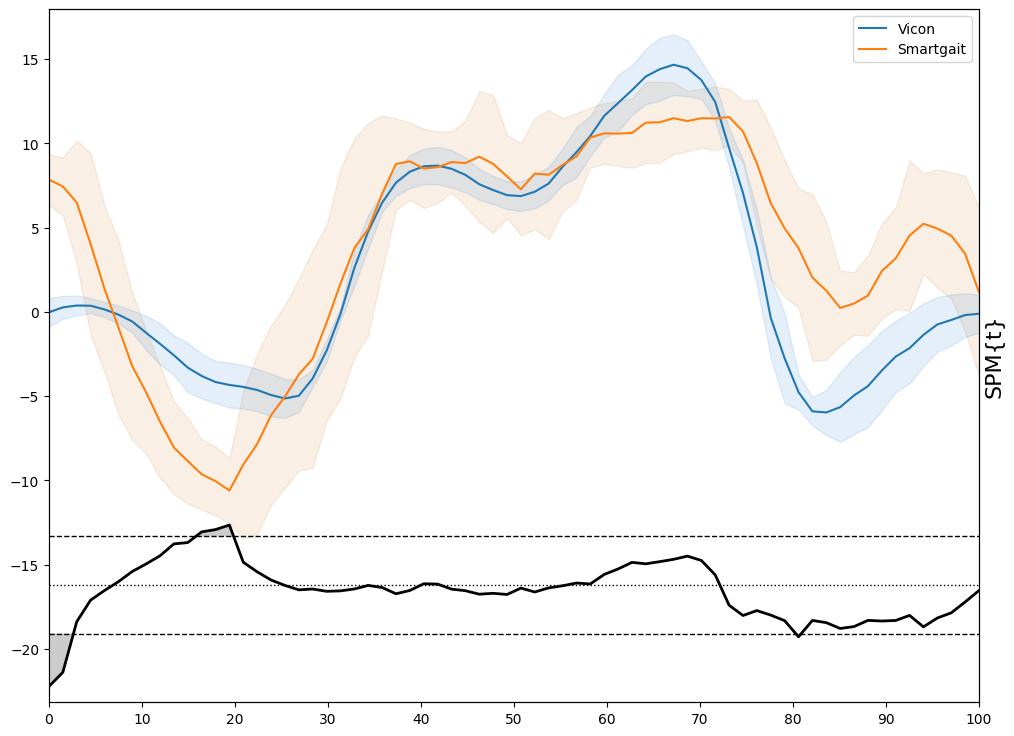

Supplement: Supplementary file 1 [file sensors-24-07819-s001.zip › spm_eval_LA13RE28_sagital/LA13RE28_angle_(5, 8, 8, 11)1.csv_plot_spm.png]

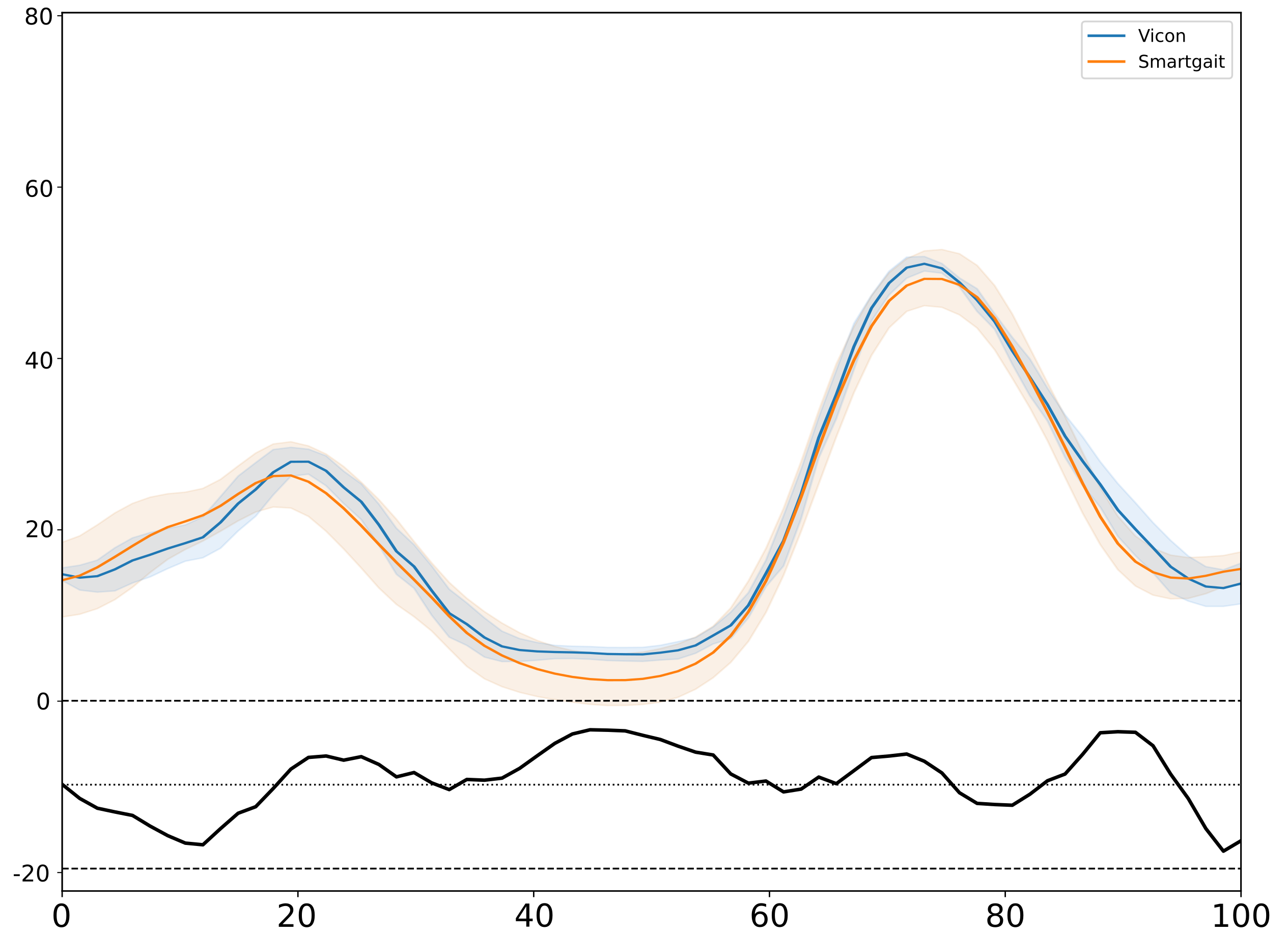

Supplement: Supplementary file 1 [file sensors-24-07819-s001.zip › spm_eval_LA13RE28_sagital/LA13RE28_angle_(2, 5, 5, 8)1.csv_plot_spm_fixed.png]

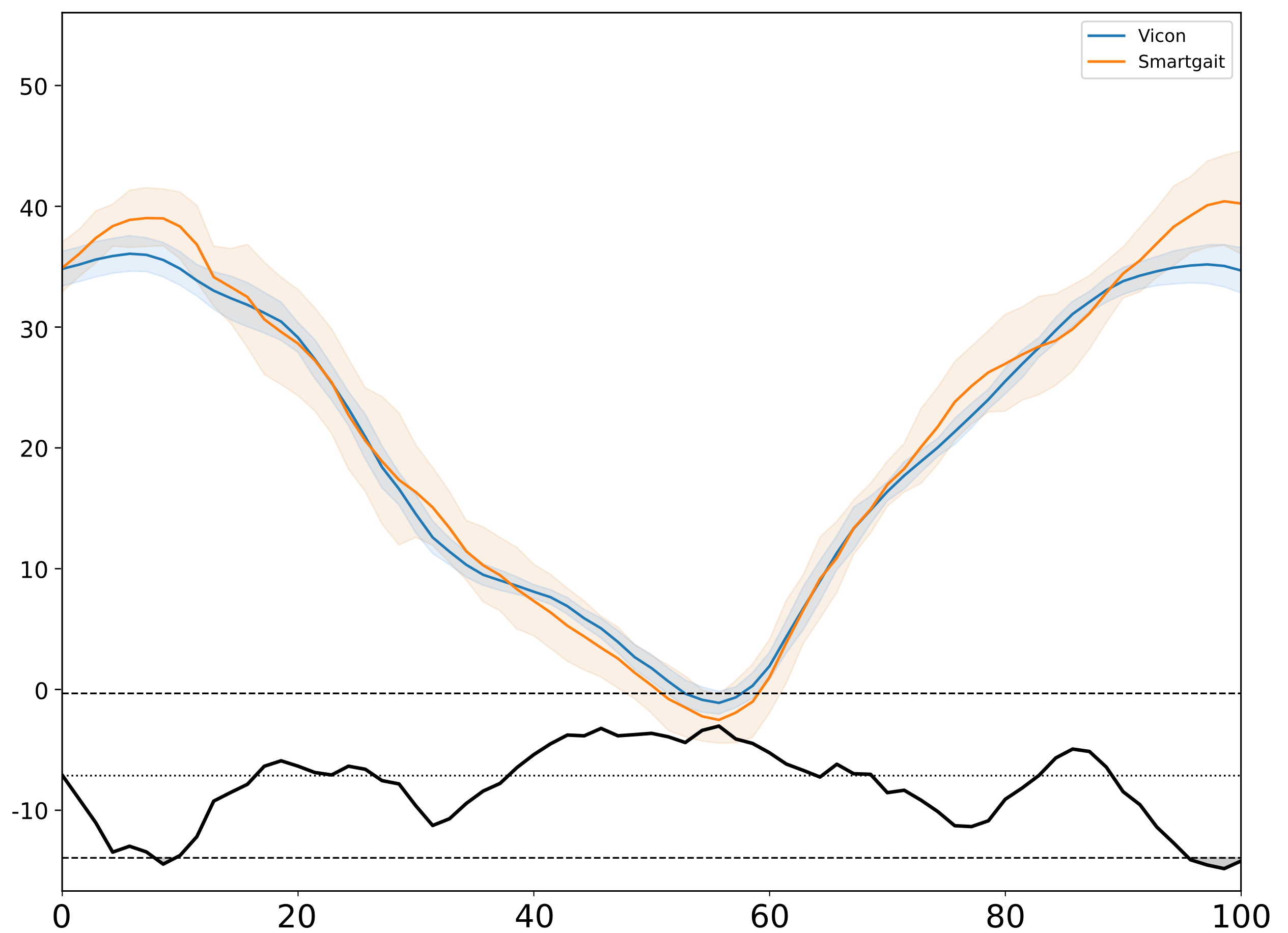

Supplement: Supplementary file 1 [file sensors-24-07819-s001.zip › spm_eval_LA13RE28_sagital/LA13RE28_angle_(2, 5, 12, 0)1.csv_plot_spm_fixed.png]

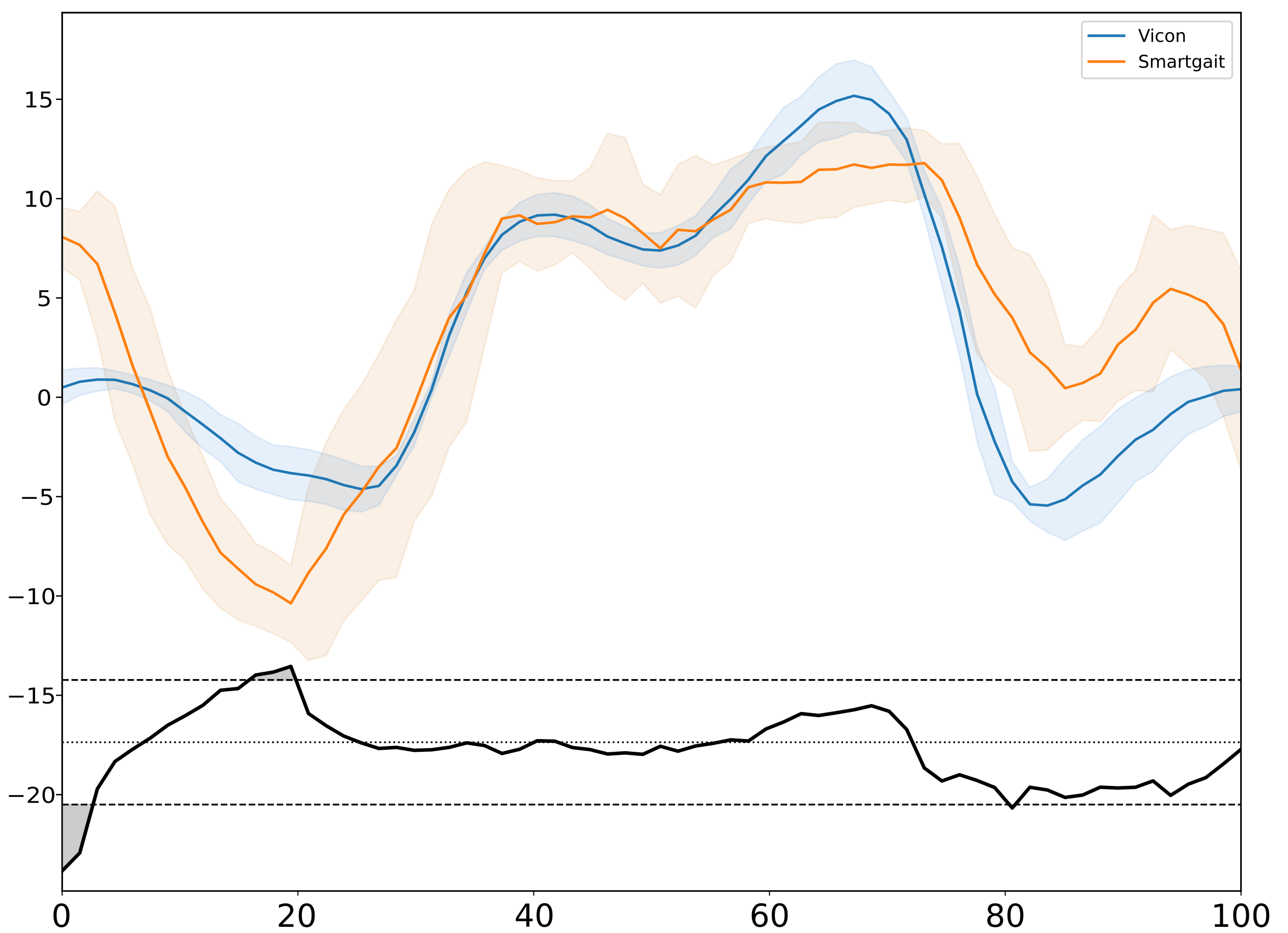

Supplement: Supplementary file 1 [file sensors-24-07819-s001.zip › spm_eval_LA13RE28_sagital/LA13RE28_angle_(5, 8, 8, 11)1.csv_plot_spm_fixed.png]

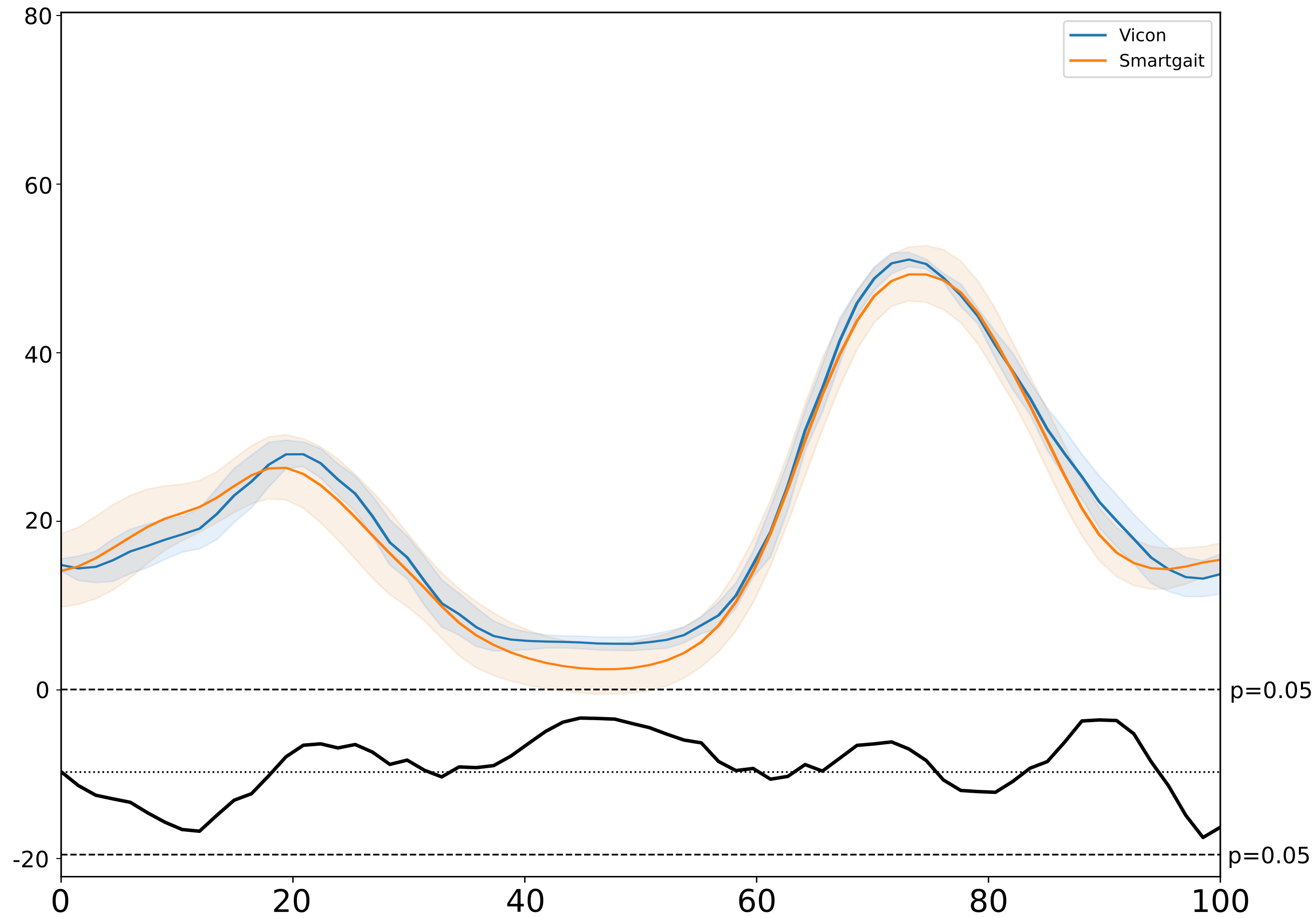

Supplement: Supplementary file 1 [file sensors-24-07819-s001.zip › spm_eval_LA13RE28_sagital/LA13RE28_angle_(2, 5, 5, 8)1.csv_plot_spm_fixed_.png]

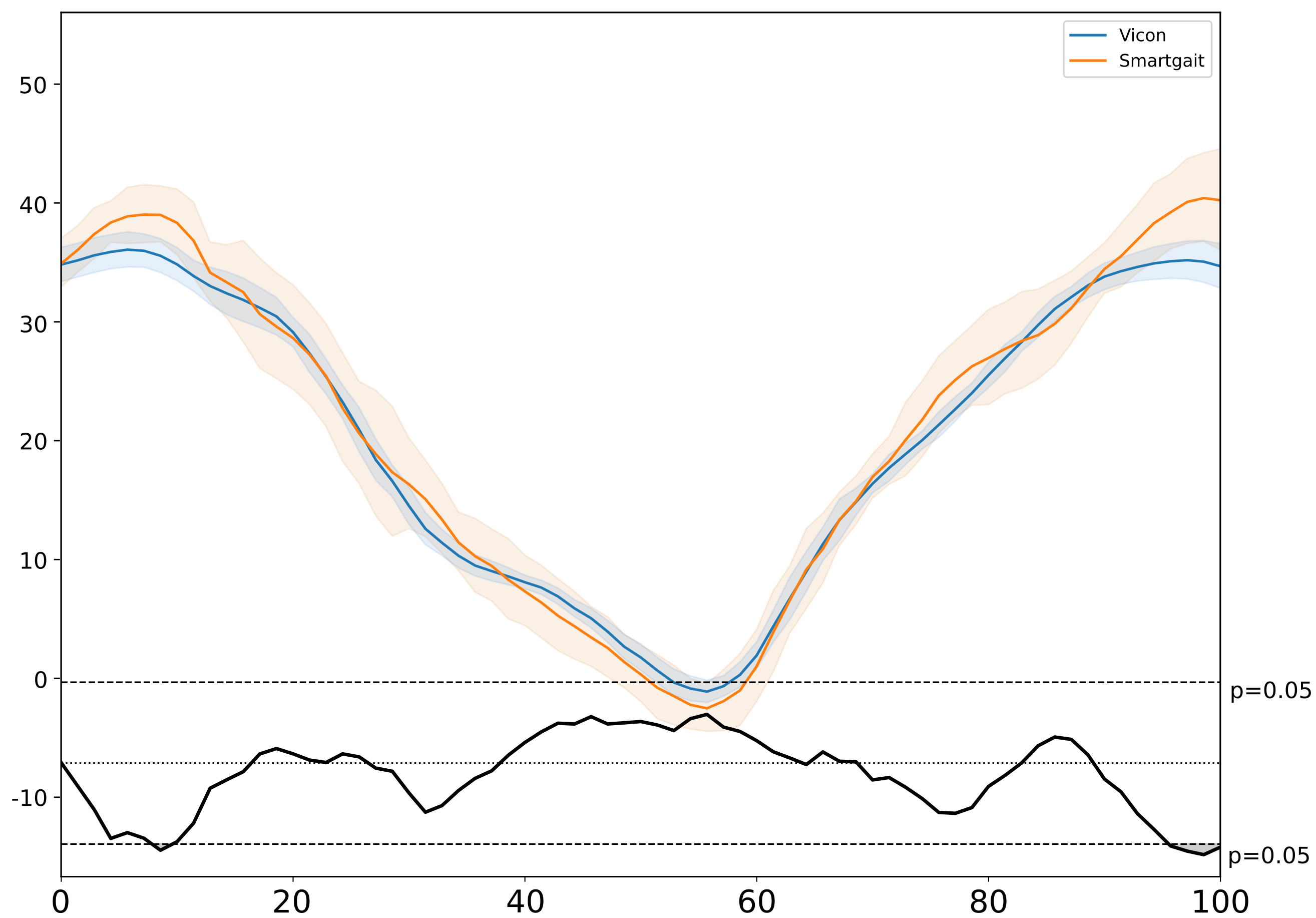

Supplement: Supplementary file 1 [file sensors-24-07819-s001.zip › spm_eval_LA13RE28_sagital/LA13RE28_angle_(2, 5, 12, 0)1.csv_plot_spm_fixed_.png]

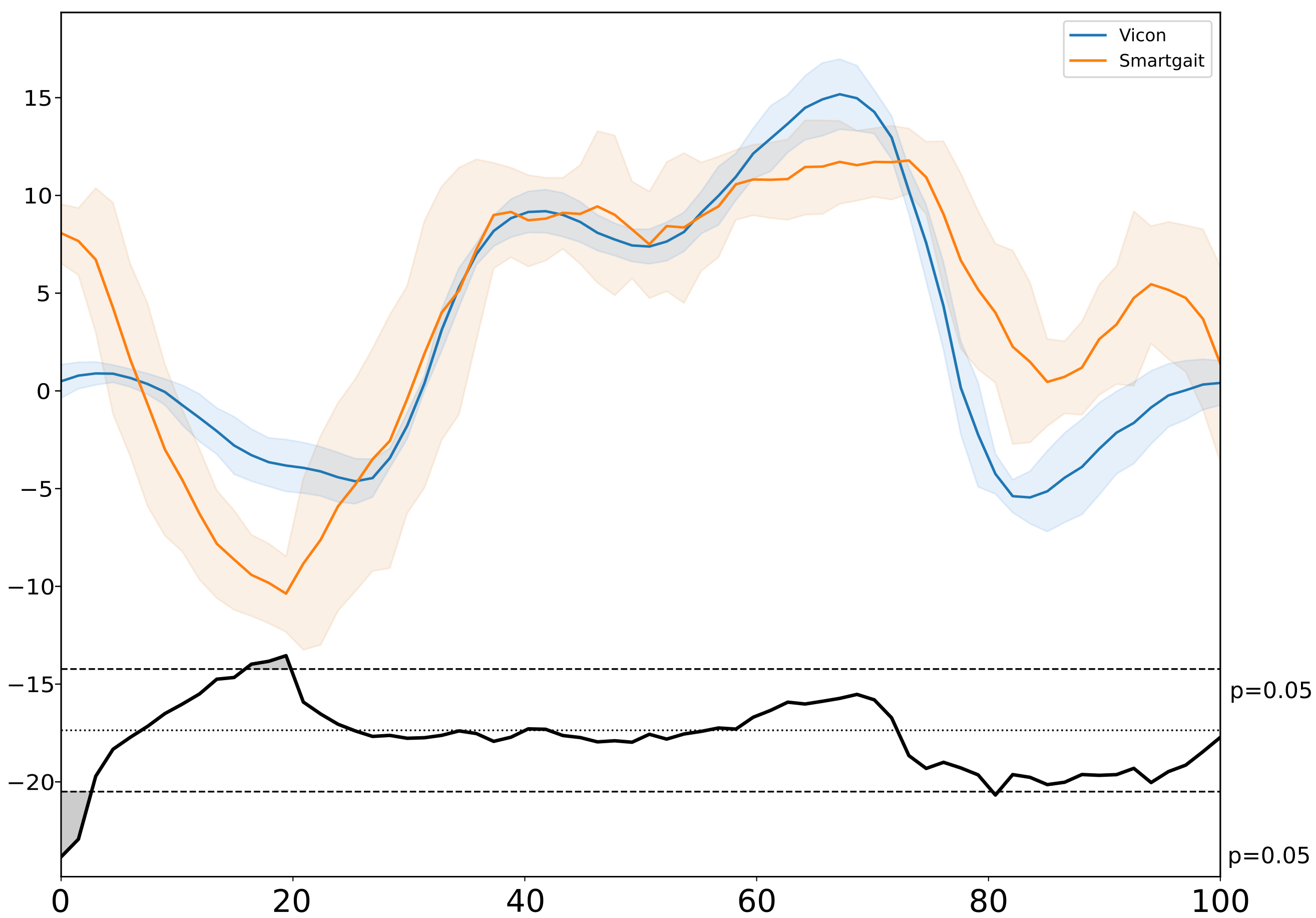

Supplement: Supplementary file 1 [file sensors-24-07819-s001.zip › spm_eval_LA13RE28_sagital/LA13RE28_angle_(5, 8, 8, 11)1.csv_plot_spm_fixed_.png]

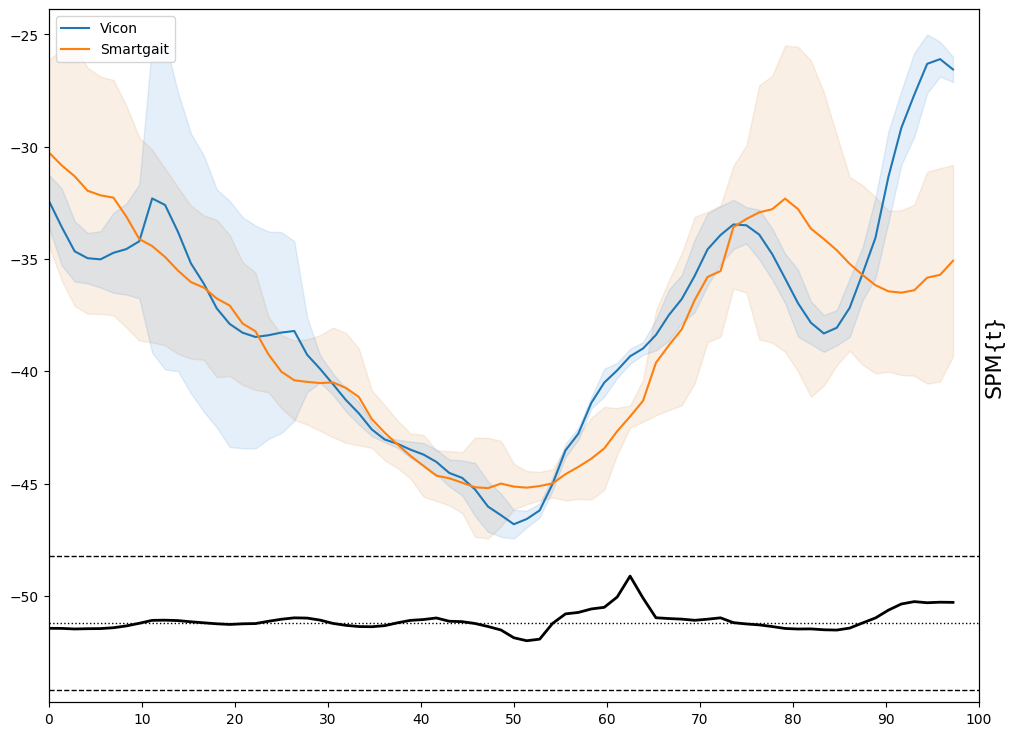

Supplement: Supplementary file 1 [file sensors-24-07819-s001.zip › spm_eval_LU09iL01_frontal/LU09iL01_angle_(2, 5, 12, 0)1.csv_plot_spm.png]

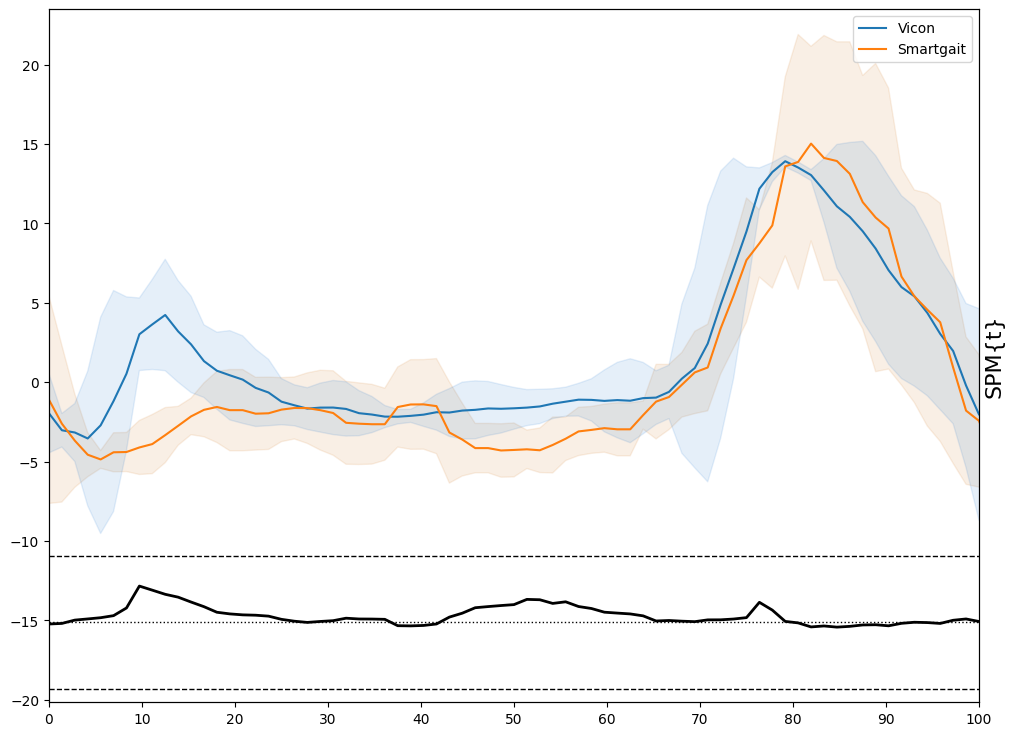

Supplement: Supplementary file 1 [file sensors-24-07819-s001.zip › spm_eval_LU09iL01_frontal/LU09iL01_angle_(2, 5, 5, 8)1.csv_plot_spm.png]

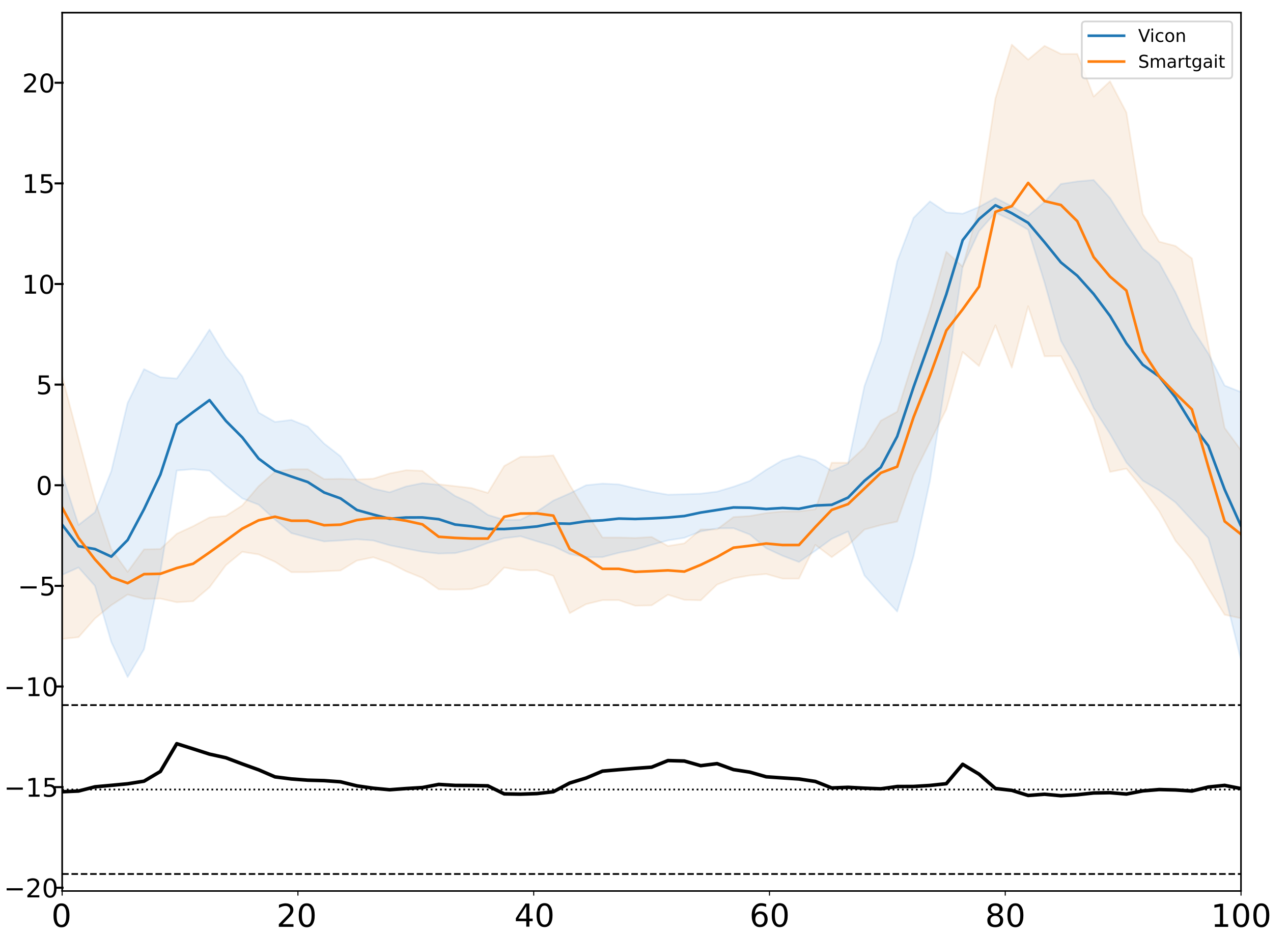

Supplement: Supplementary file 1 [file sensors-24-07819-s001.zip › spm_eval_LU09iL01_frontal/LU09iL01_angle_(2, 5, 5, 8)1.csv_plot_spm_fixed.png]

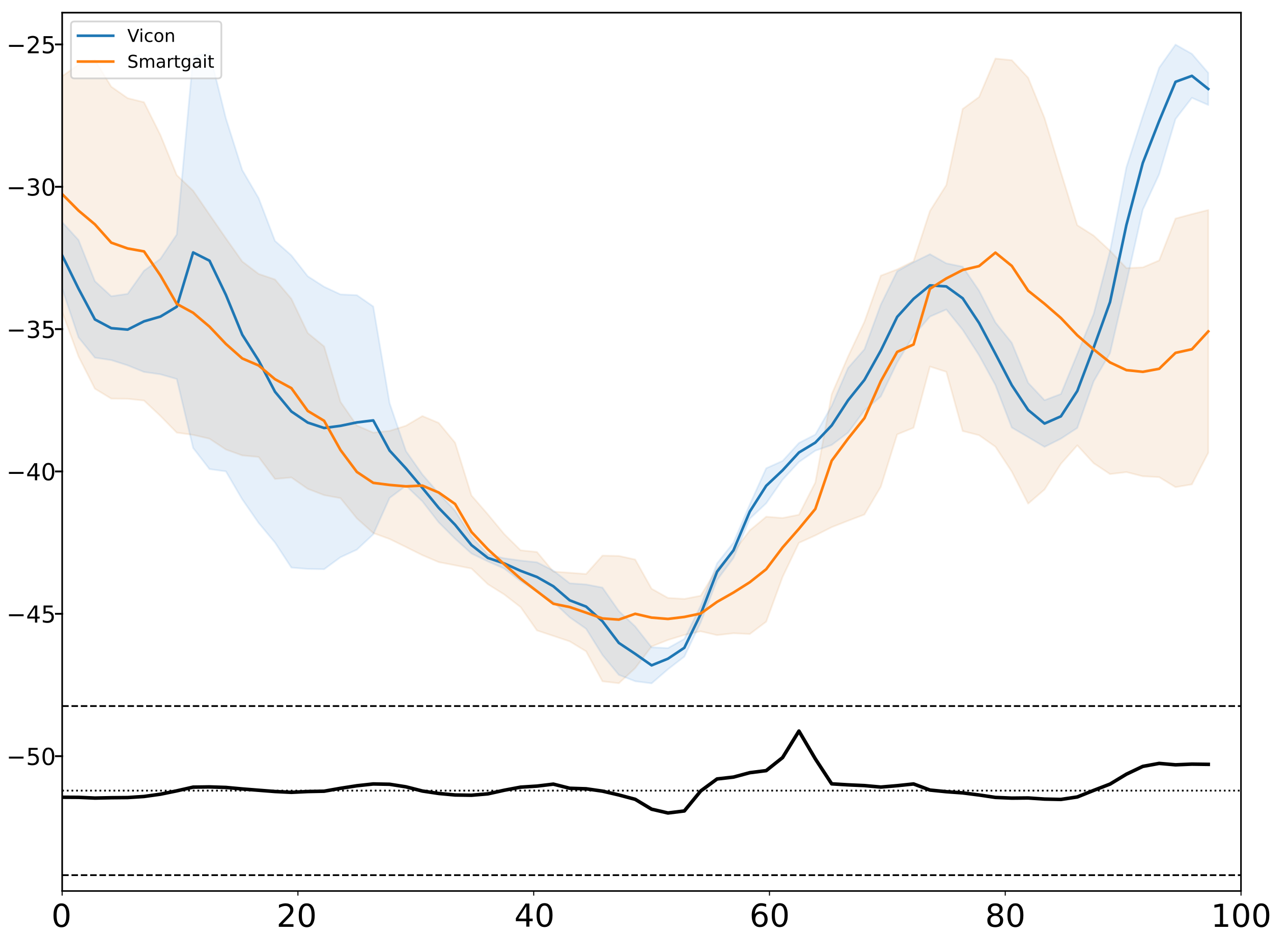

Supplement: Supplementary file 1 [file sensors-24-07819-s001.zip › spm_eval_LU09iL01_frontal/LU09iL01_angle_(2, 5, 12, 0)1.csv_plot_spm_fixed.png]

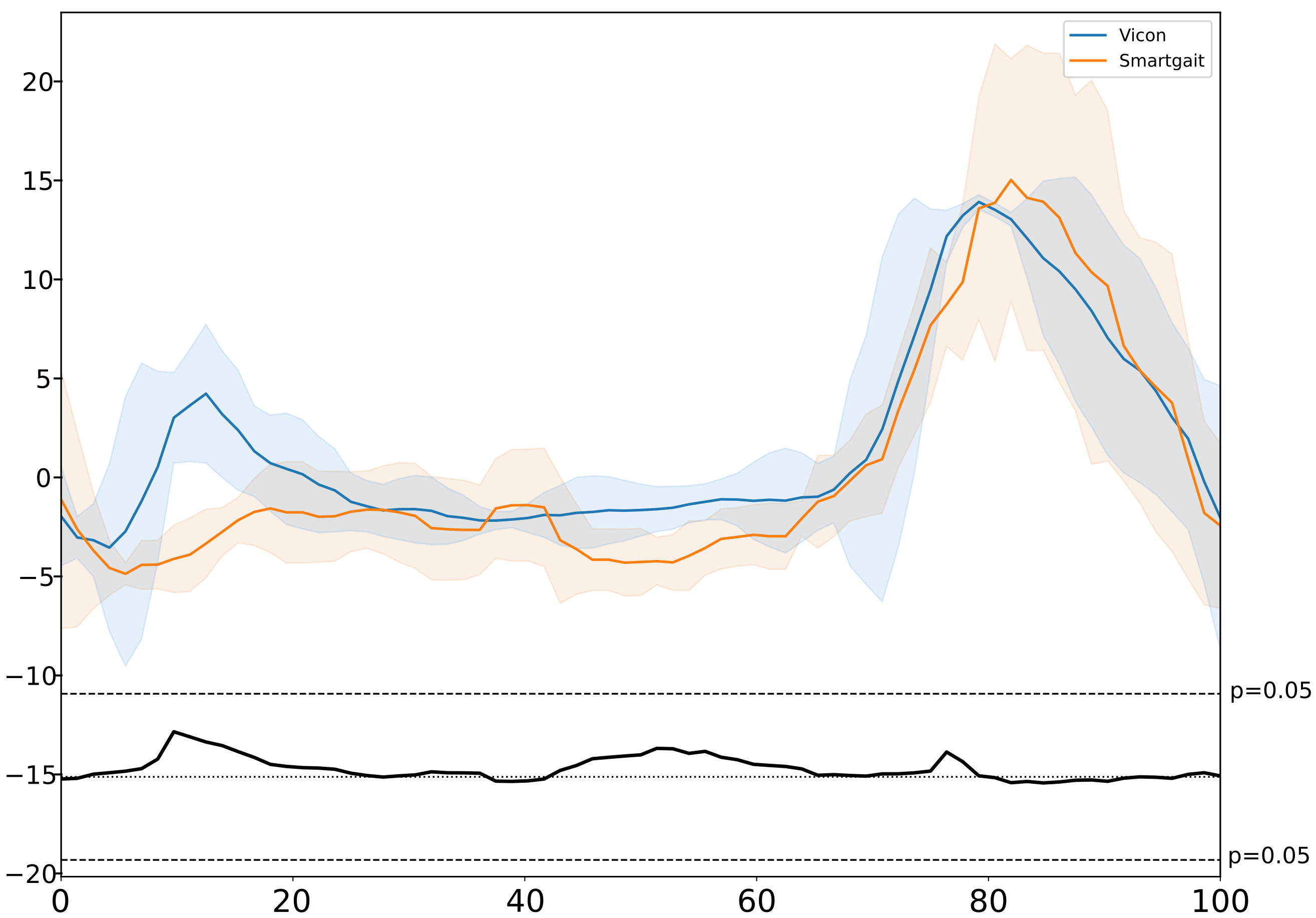

Supplement: Supplementary file 1 [file sensors-24-07819-s001.zip › spm_eval_LU09iL01_frontal/LU09iL01_angle_(2, 5, 5, 8)1.csv_plot_spm_fixed_.png]

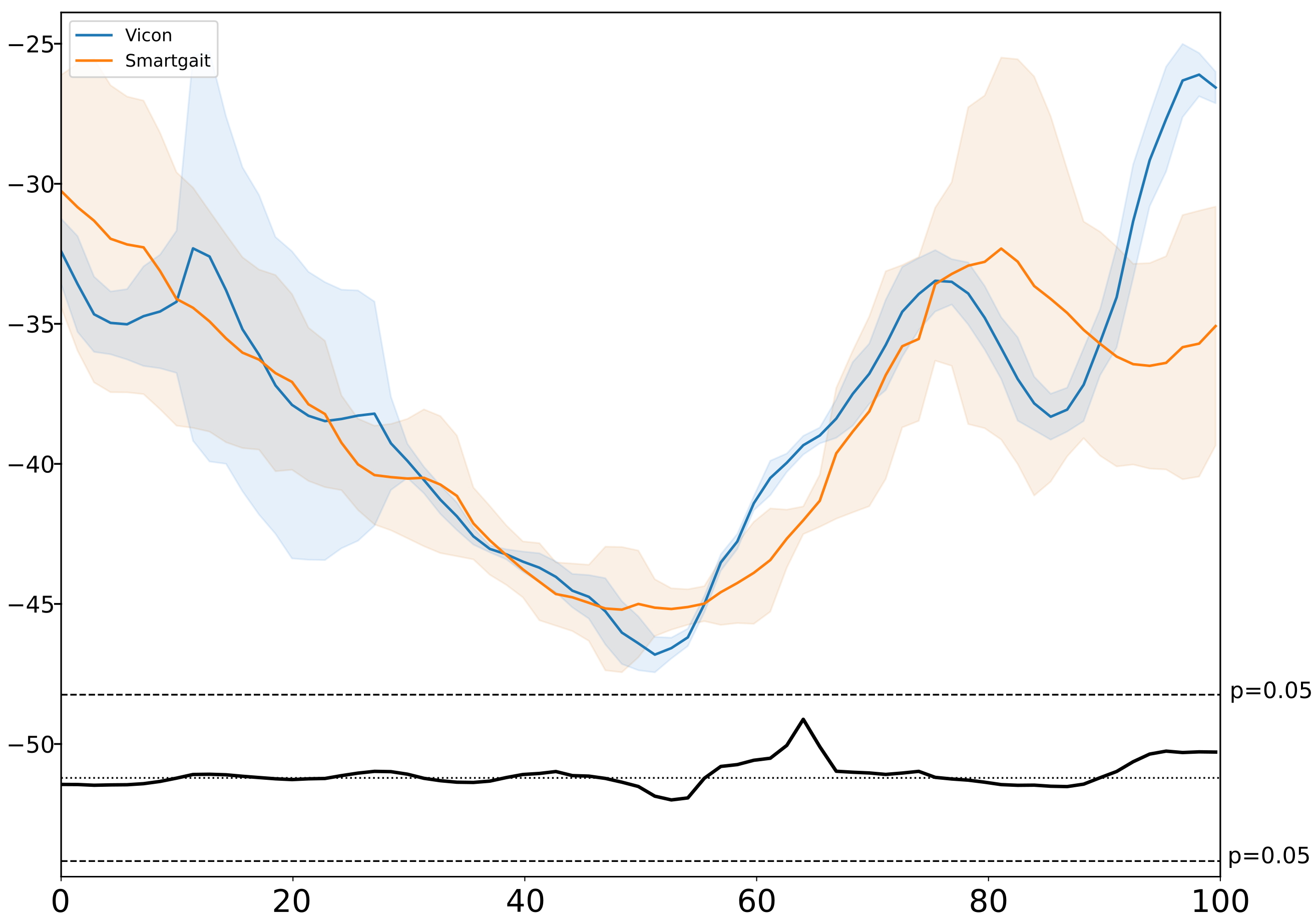

Supplement: Supplementary file 1 [file sensors-24-07819-s001.zip › spm_eval_LU09iL01_frontal/LU09iL01_angle_(2, 5, 12, 0)1.csv_plot_spm_fixed_.png]

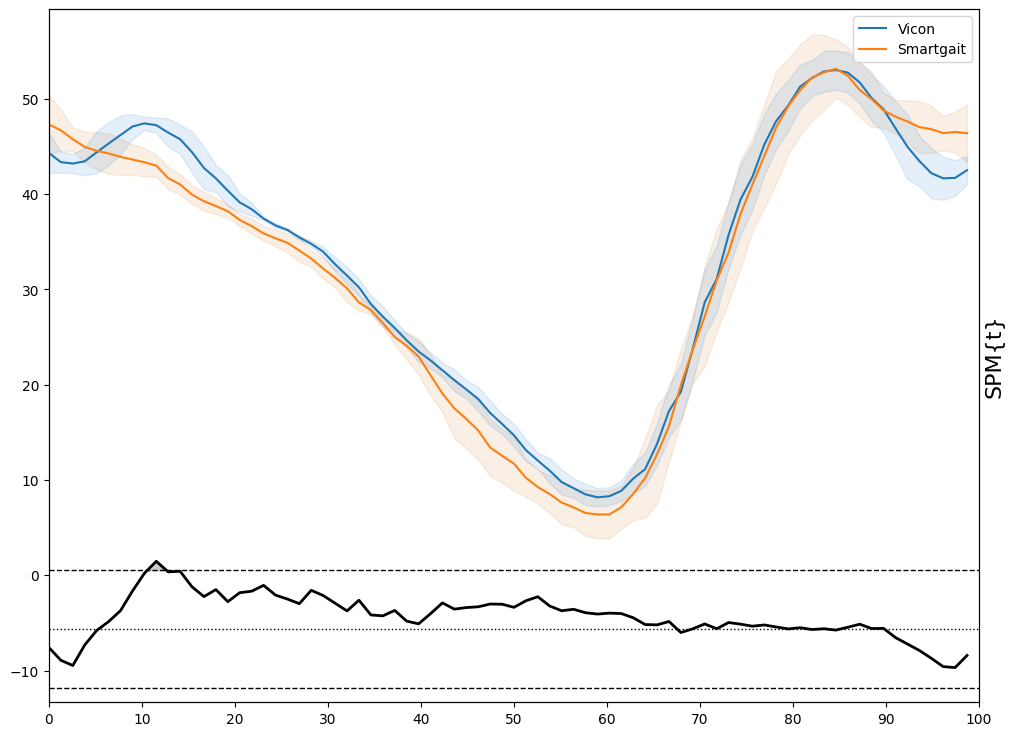

Supplement: Supplementary file 1 [file sensors-24-07819-s001.zip › spm_eval_LU09iL01_sagital/LU09iL01_angle_(2, 5, 12, 0)1.csv_plot_spm.png]

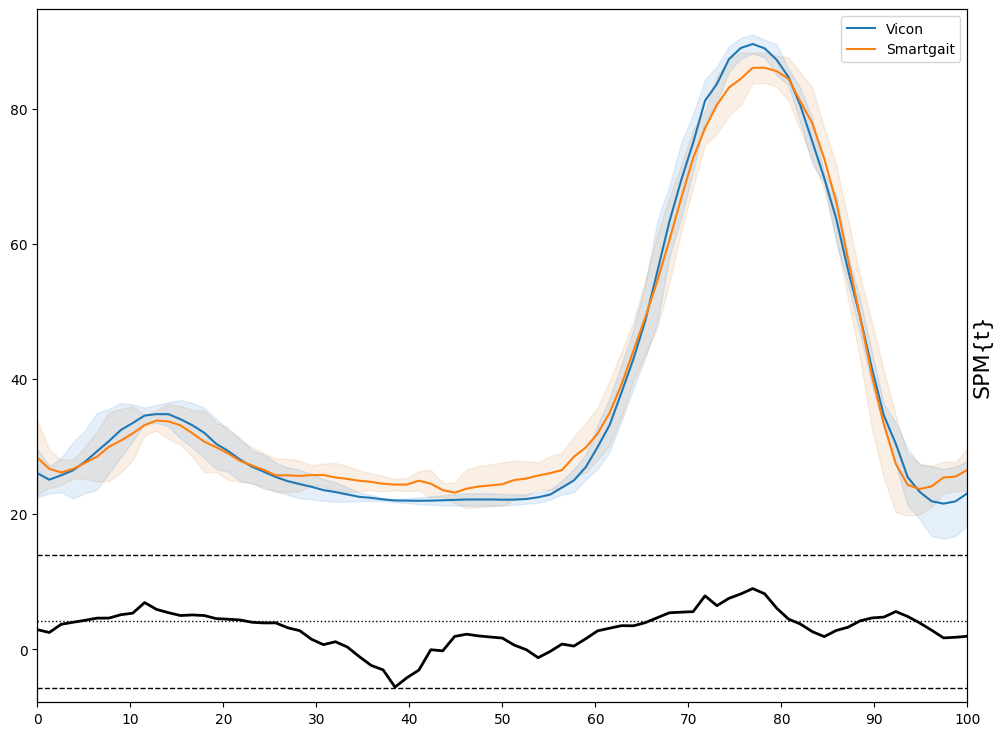

Supplement: Supplementary file 1 [file sensors-24-07819-s001.zip › spm_eval_LU09iL01_sagital/LU09iL01_angle_(2, 5, 5, 8)1.csv_plot_spm.png]

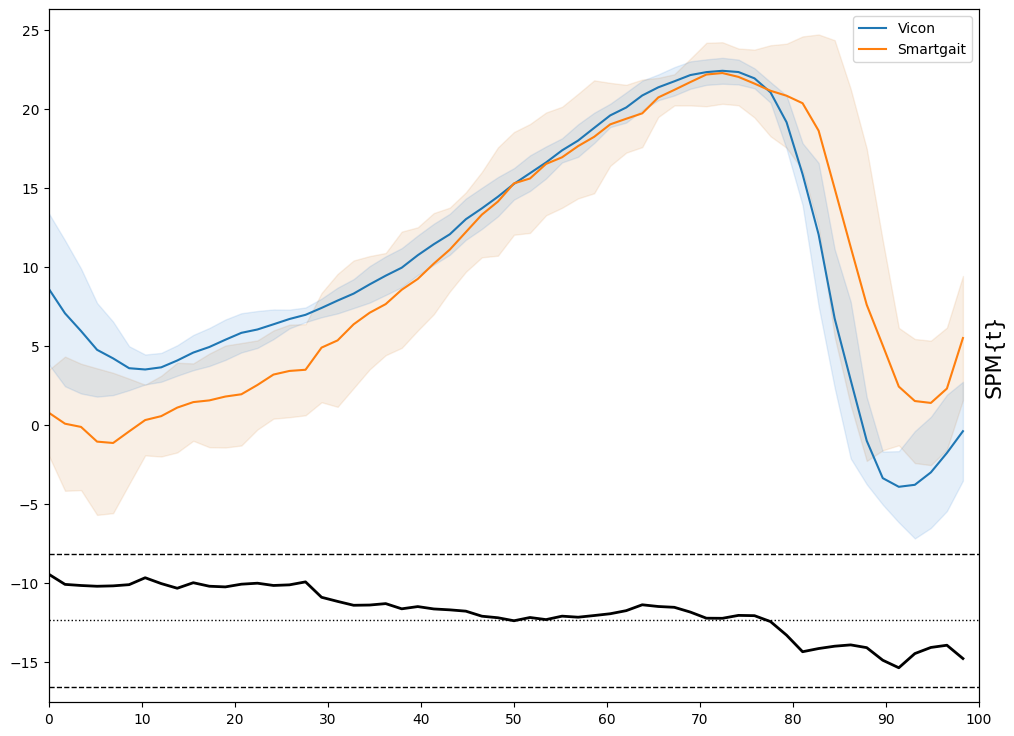

Supplement: Supplementary file 1 [file sensors-24-07819-s001.zip › spm_eval_LU09iL01_sagital/LU09iL01_angle_(5, 8, 8, 11)1.csv_plot_spm.png]

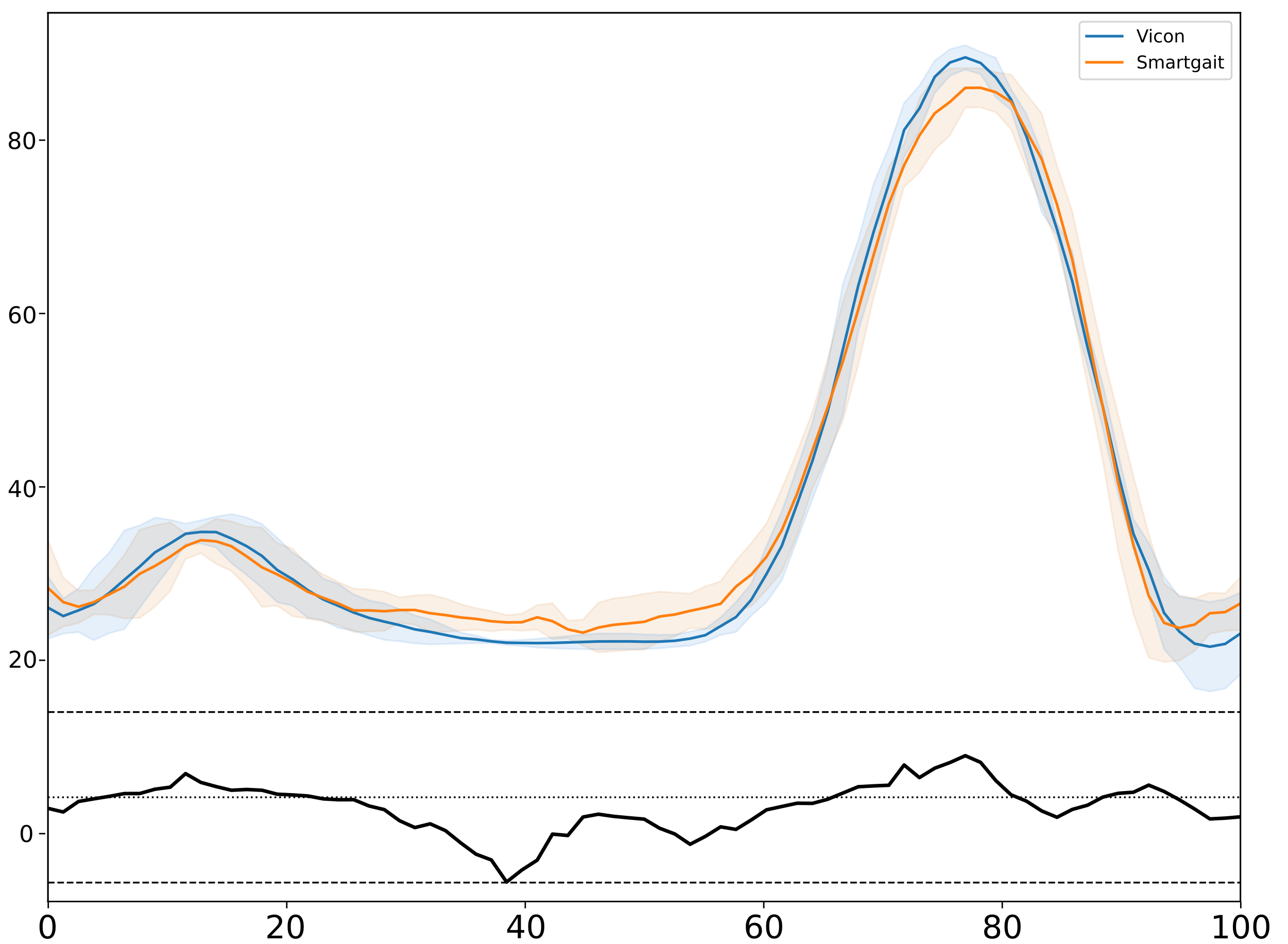

Supplement: Supplementary file 1 [file sensors-24-07819-s001.zip › spm_eval_LU09iL01_sagital/LU09iL01_angle_(2, 5, 5, 8)1.csv_plot_spm_fixed.png]

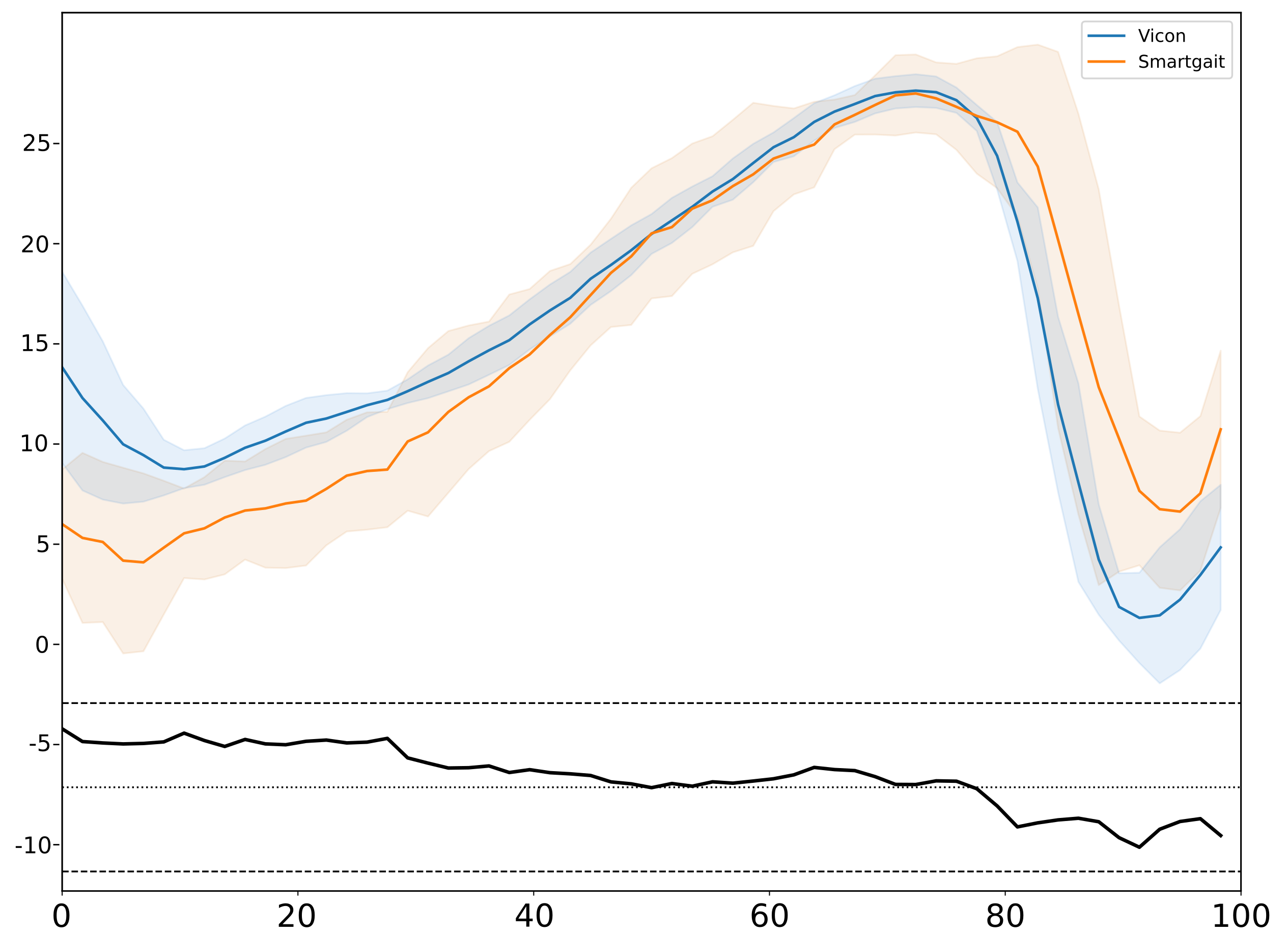

Supplement: Supplementary file 1 [file sensors-24-07819-s001.zip › spm_eval_LU09iL01_sagital/LU09iL01_angle_(5, 8, 8, 11)1.csv_plot_spm_fixed.png]

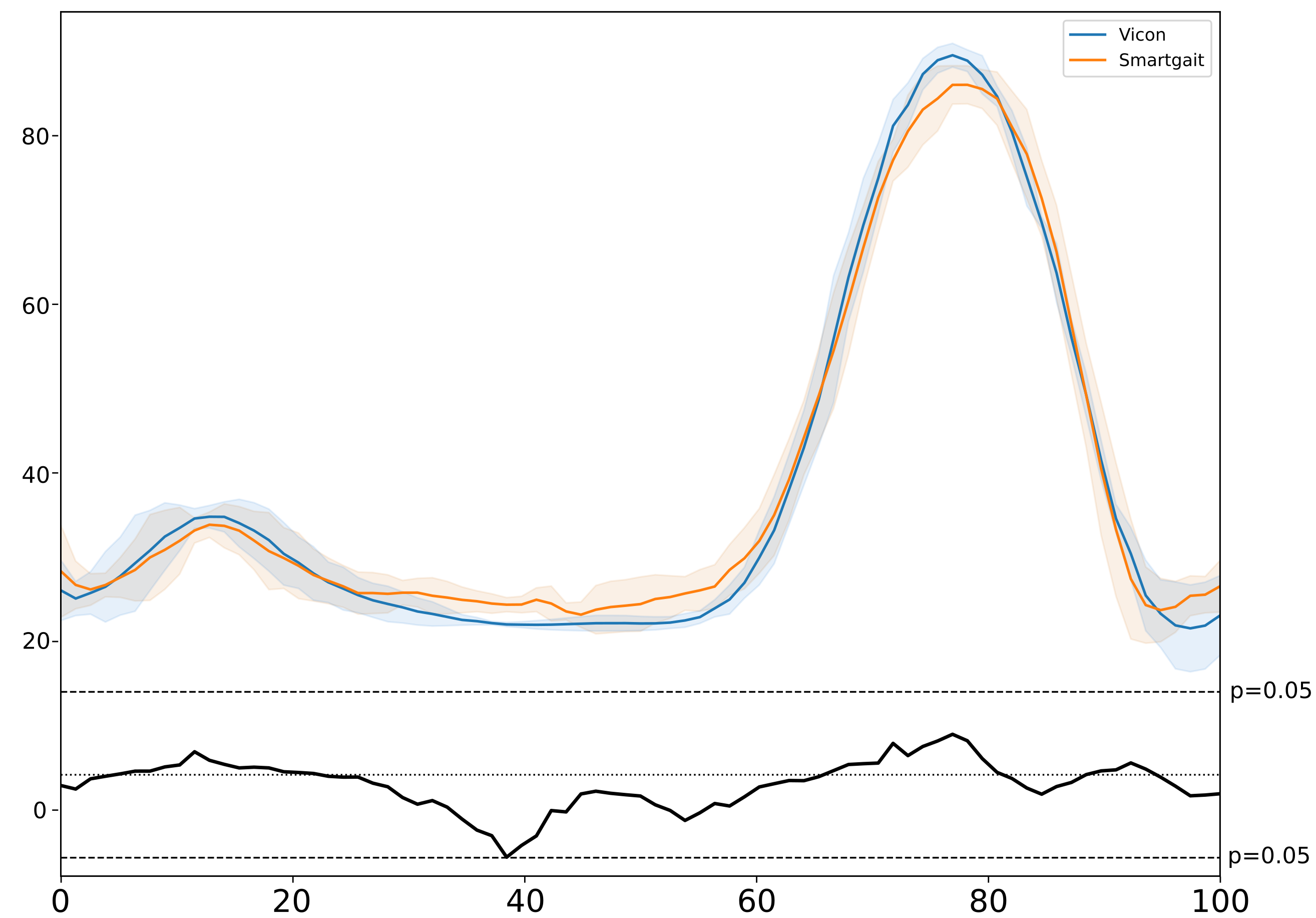

Supplement: Supplementary file 1 [file sensors-24-07819-s001.zip › spm_eval_LU09iL01_sagital/LU09iL01_angle_(2, 5, 5, 8)1.csv_plot_spm_fixed_.png]

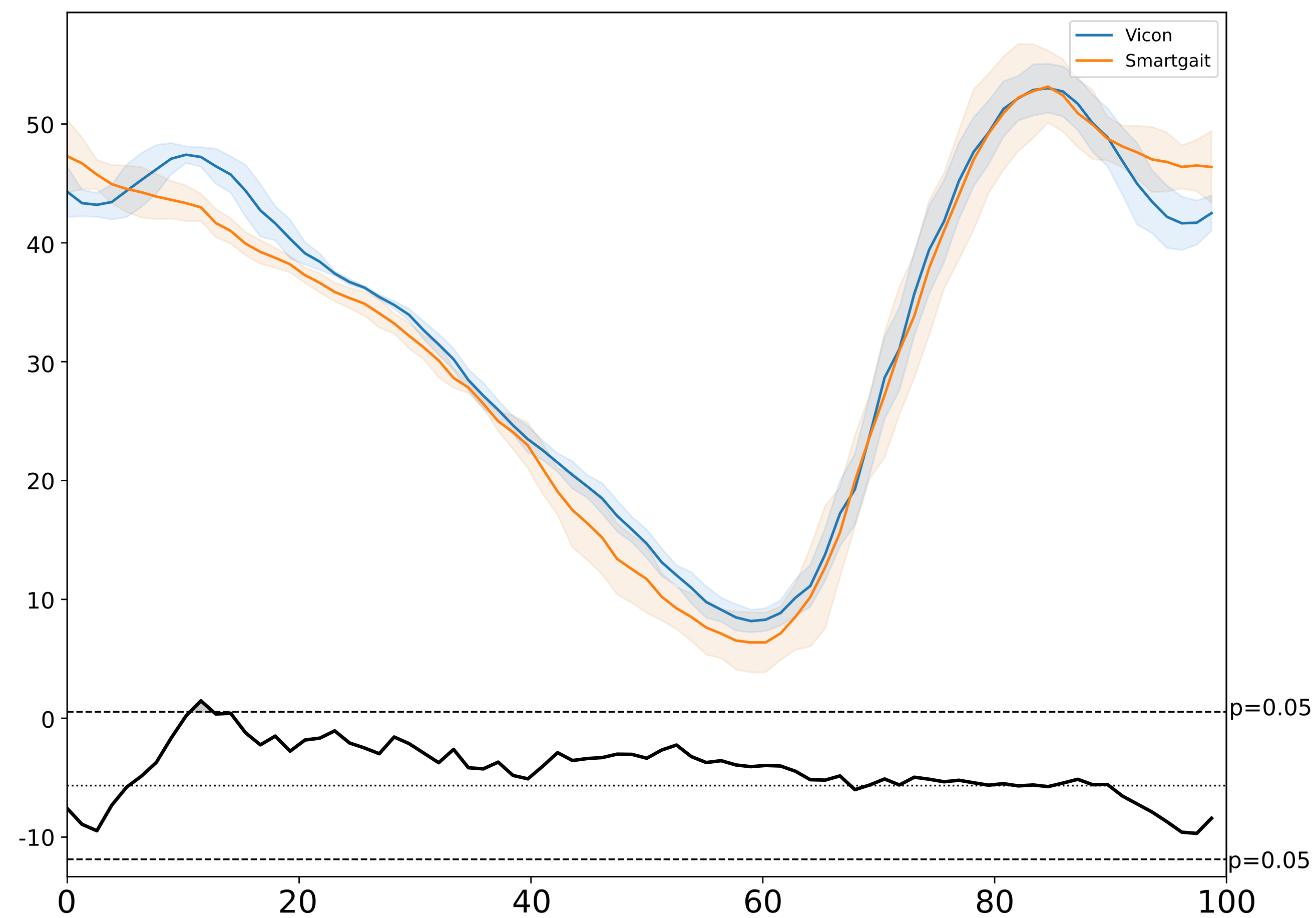

Supplement: Supplementary file 1 [file sensors-24-07819-s001.zip › spm_eval_LU09iL01_sagital/LU09iL01_angle_(2, 5, 12, 0)1.csv_plot_spm_fixed_.png]

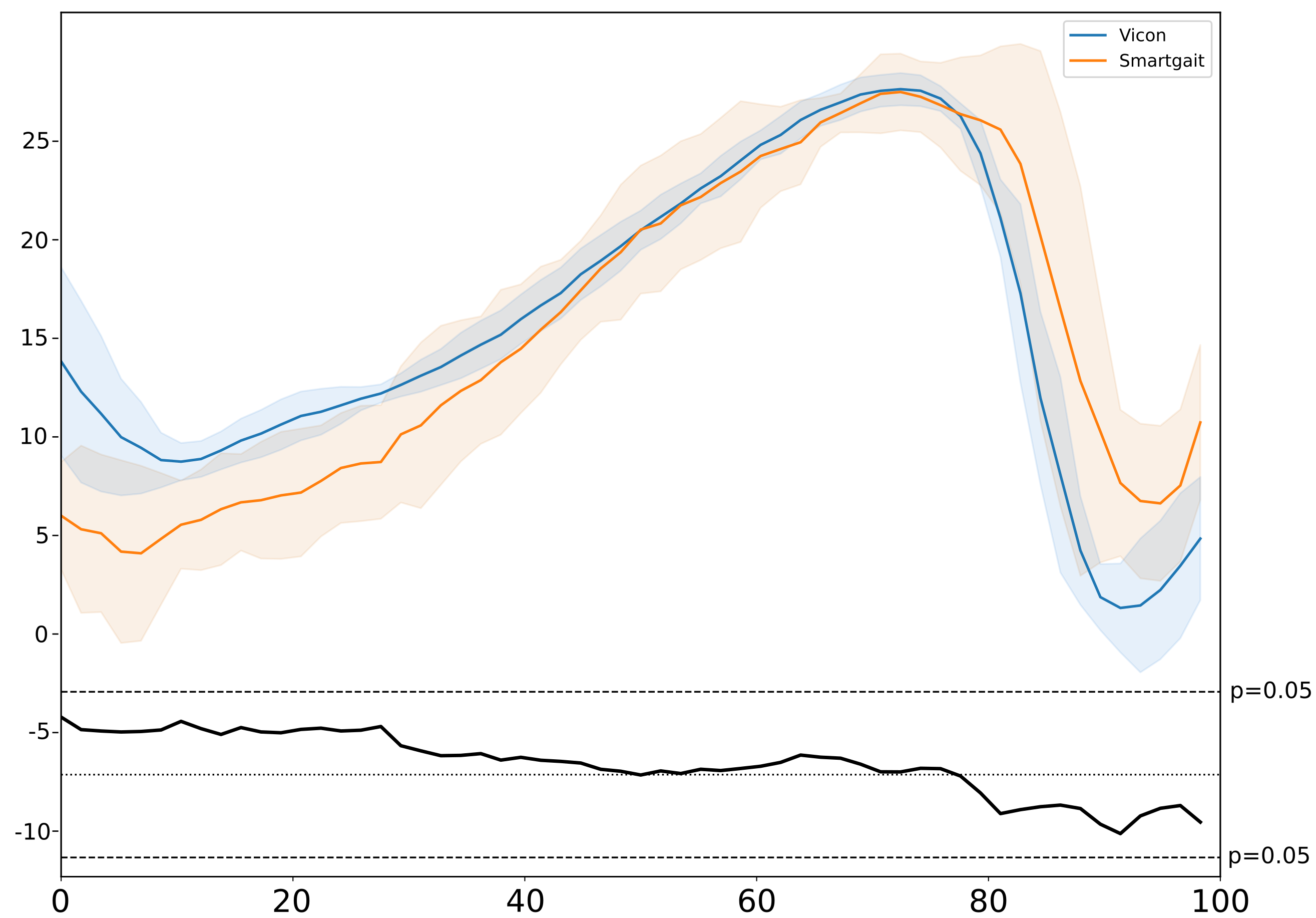

Supplement: Supplementary file 1 [file sensors-24-07819-s001.zip › spm_eval_LU09iL01_sagital/LU09iL01_angle_(5, 8, 8, 11)1.csv_plot_spm_fixed_.png]

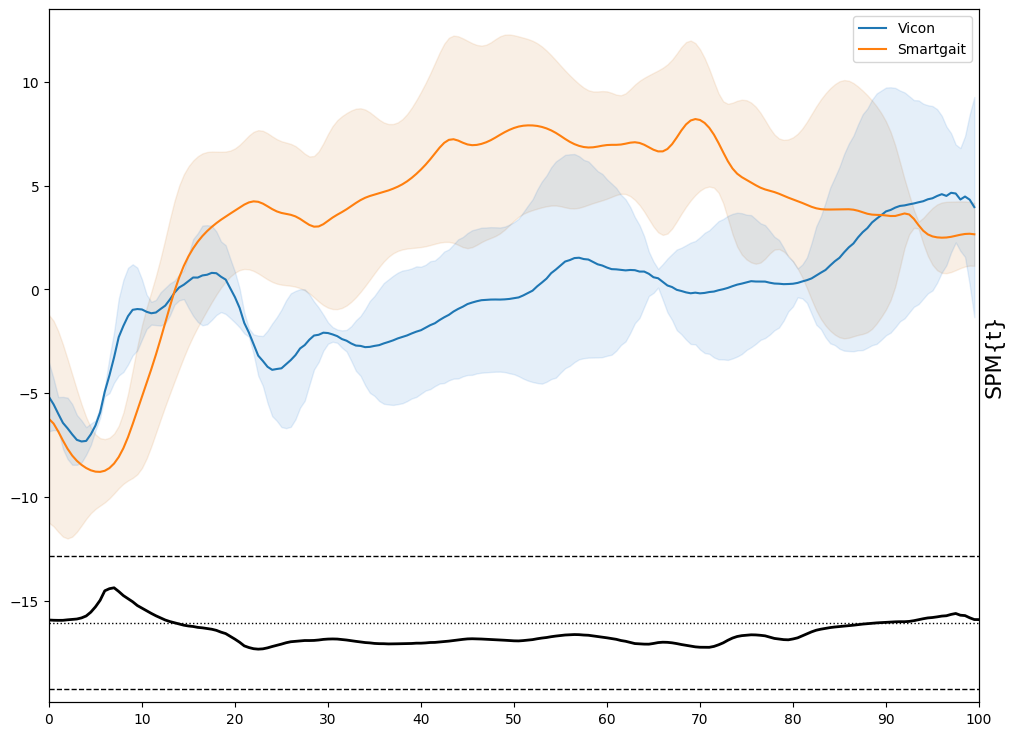

Supplement: Supplementary file 1 [file sensors-24-07819-s001.zip › spm_eval_MA03AL08_frontal/MA03AL08_angle_(2, 5, 12, 0)2.csv_plot_spm.png]

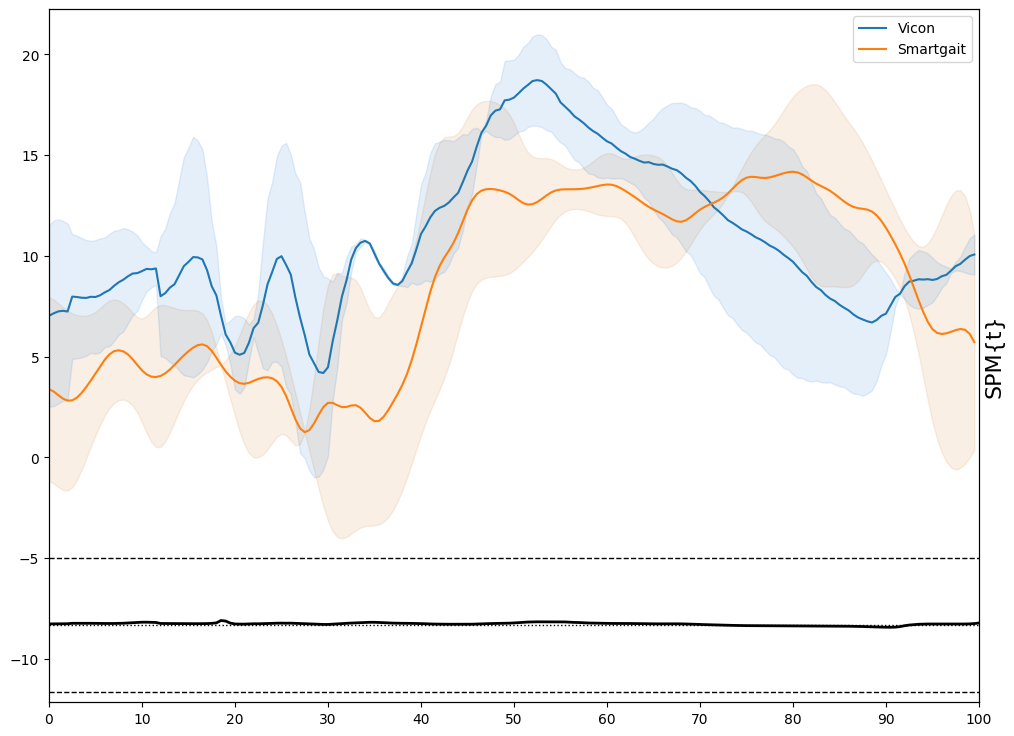

Supplement: Supplementary file 1 [file sensors-24-07819-s001.zip › spm_eval_MA03AL08_frontal/MA03AL08_angle_(2, 5, 5, 8)1.csv_plot_spm.png]

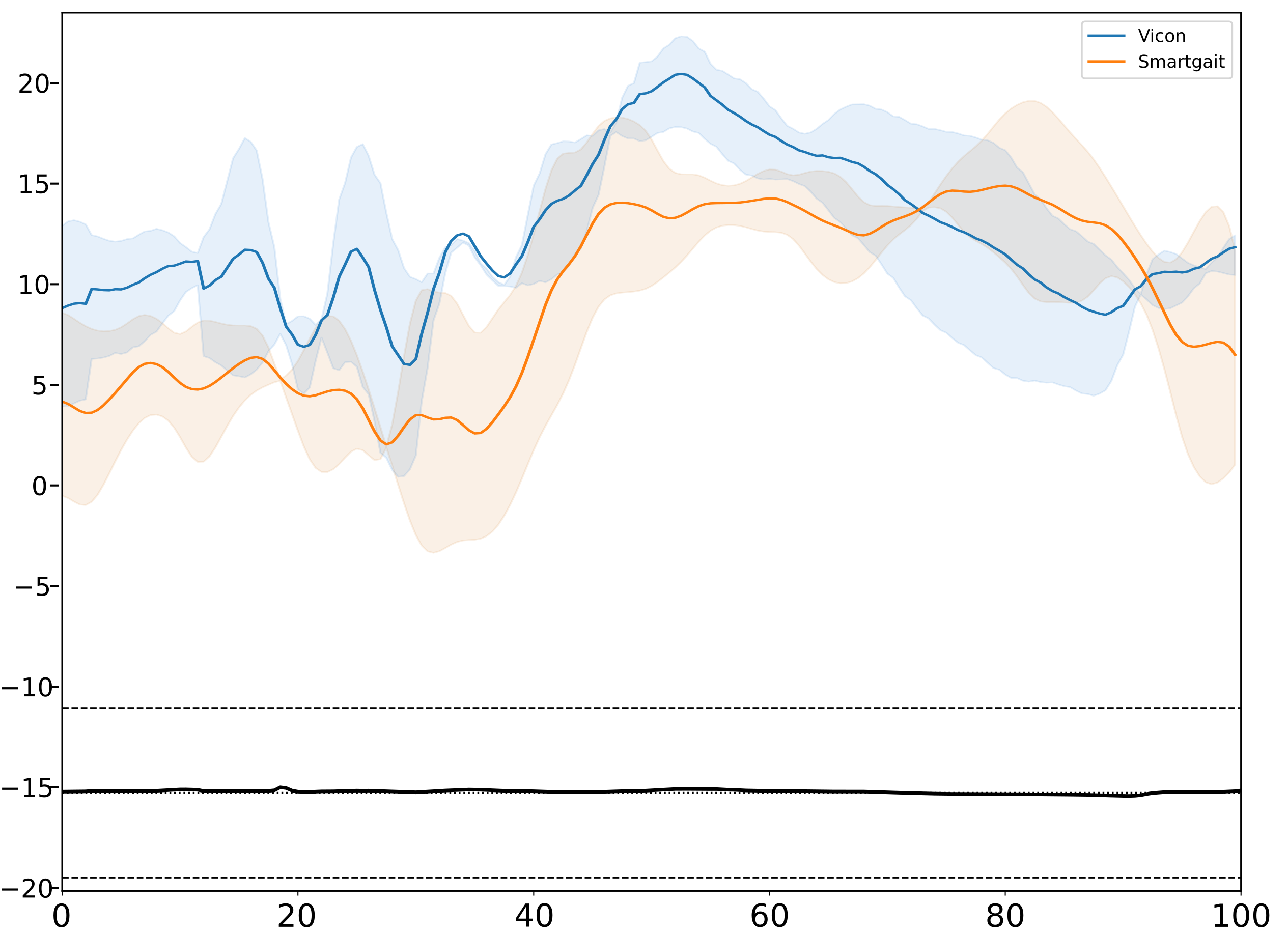

Supplement: Supplementary file 1 [file sensors-24-07819-s001.zip › spm_eval_MA03AL08_frontal/MA03AL08_angle_(2, 5, 5, 8)1.csv_plot_spm_fixed.png]

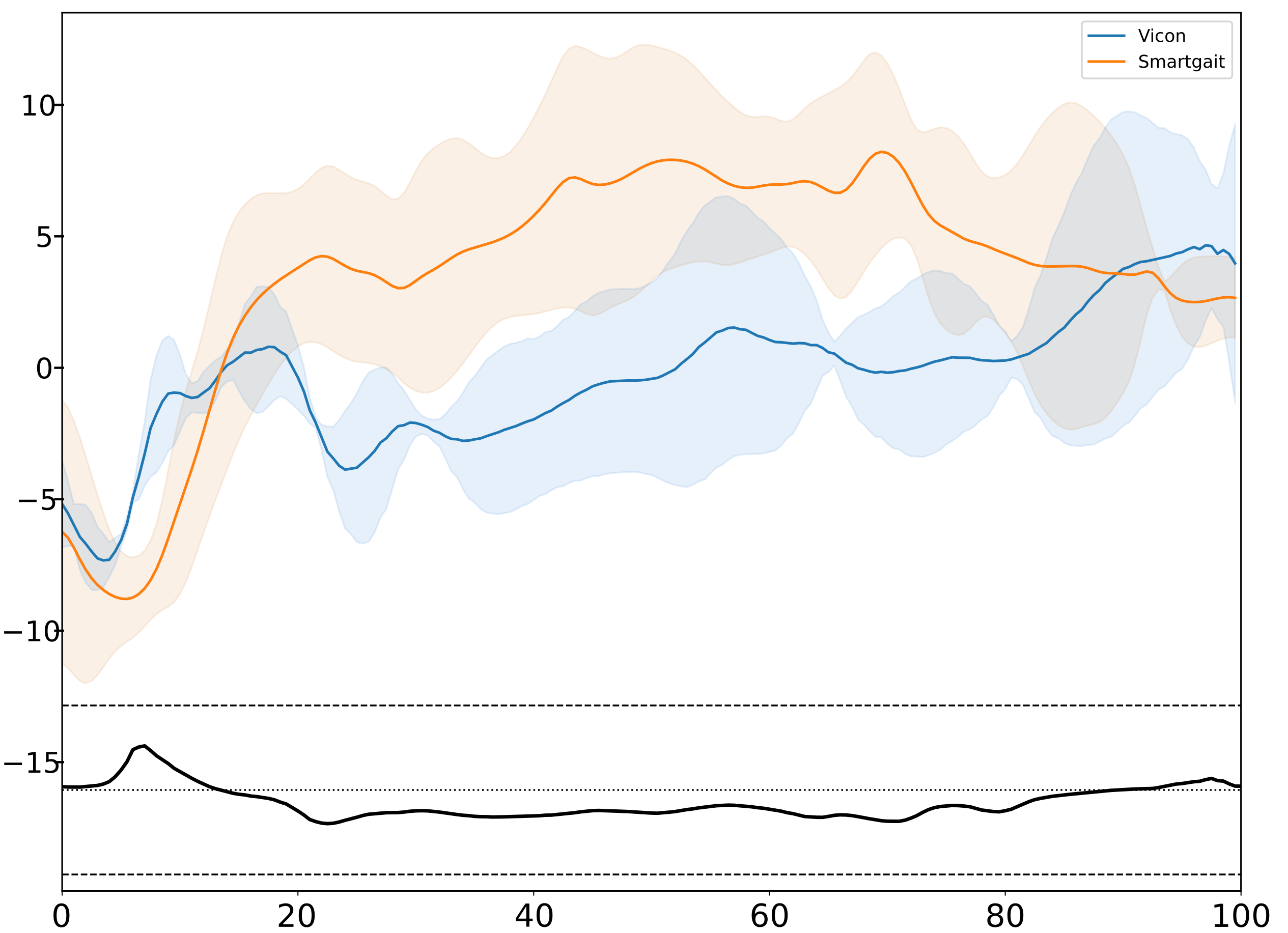

Supplement: Supplementary file 1 [file sensors-24-07819-s001.zip › spm_eval_MA03AL08_frontal/MA03AL08_angle_(2, 5, 12, 0)2.csv_plot_spm_fixed.png]

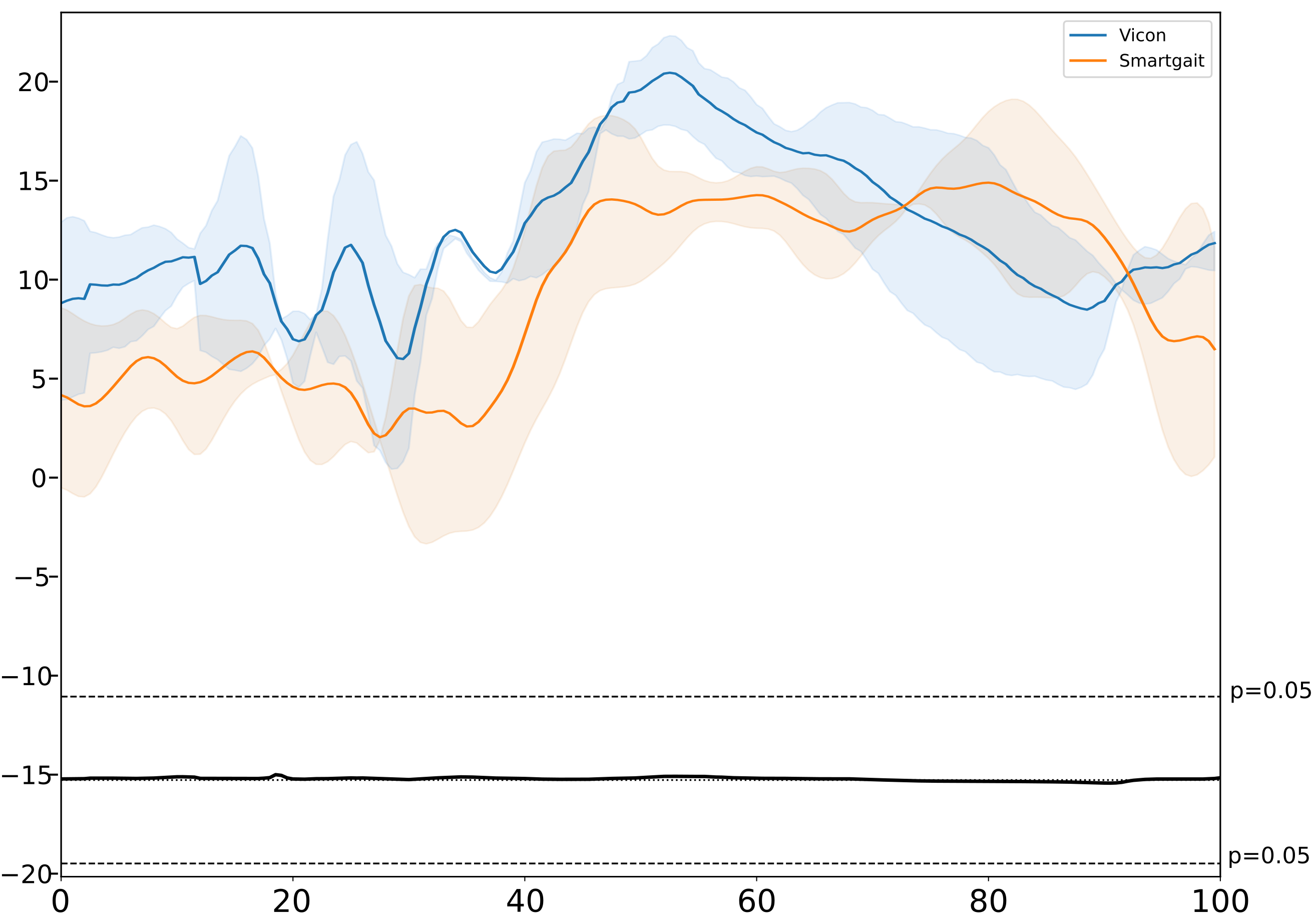

Supplement: Supplementary file 1 [file sensors-24-07819-s001.zip › spm_eval_MA03AL08_frontal/MA03AL08_angle_(2, 5, 5, 8)1.csv_plot_spm_fixed_.png]

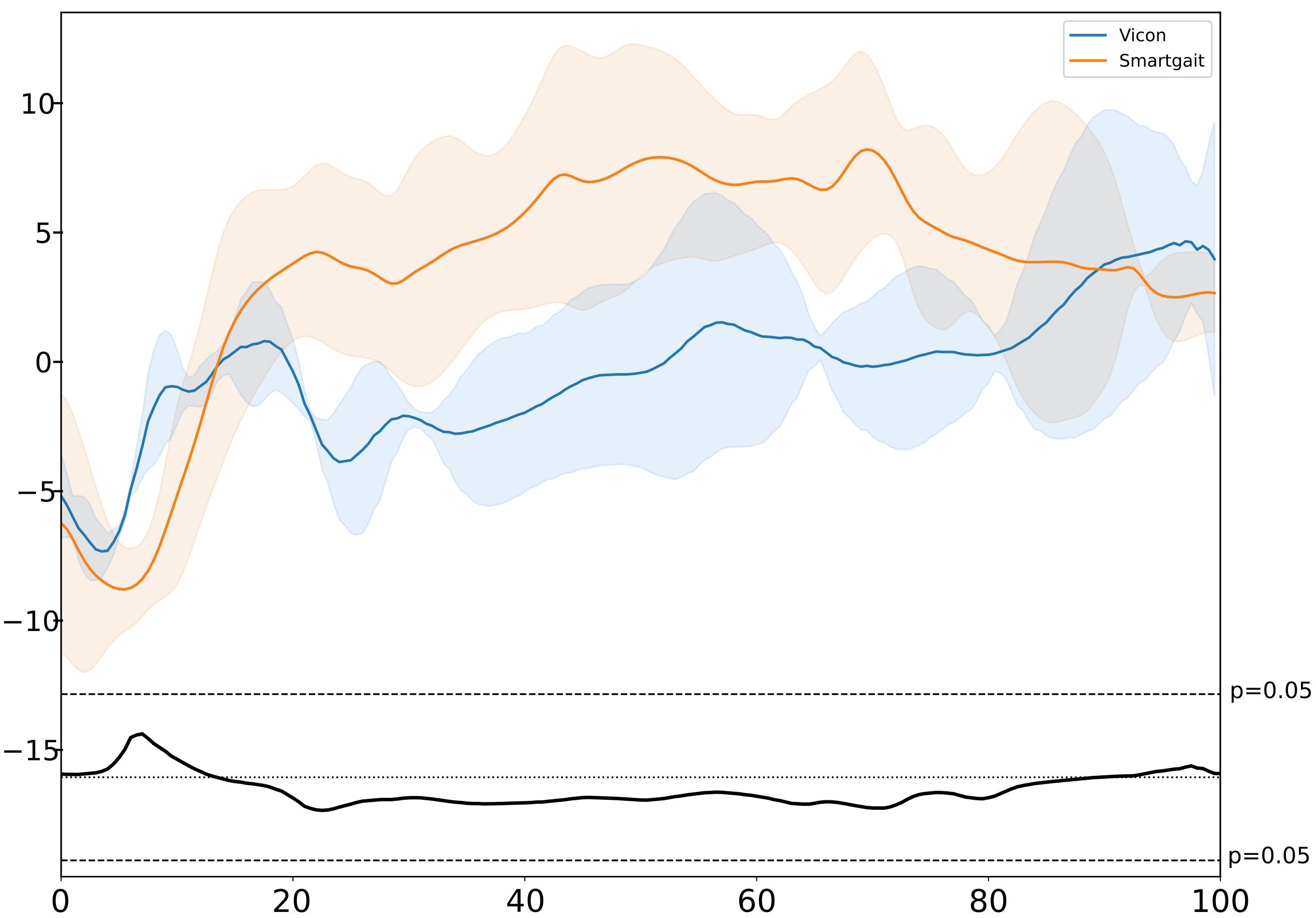

Supplement: Supplementary file 1 [file sensors-24-07819-s001.zip › spm_eval_MA03AL08_frontal/MA03AL08_angle_(2, 5, 12, 0)2.csv_plot_spm_fixed_.png]

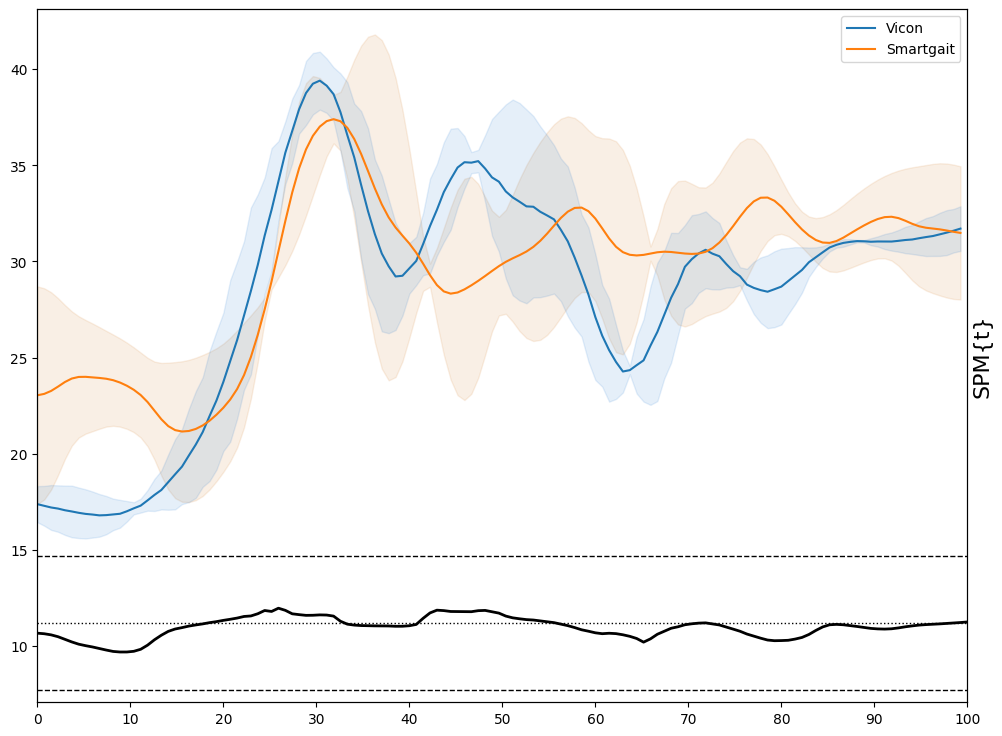

Supplement: Supplementary file 1 [file sensors-24-07819-s001.zip › spm_eval_MA03AL08_sagital/MA03AL08_angle_(2, 5, 5, 8)2.csv_plot_spm.png]

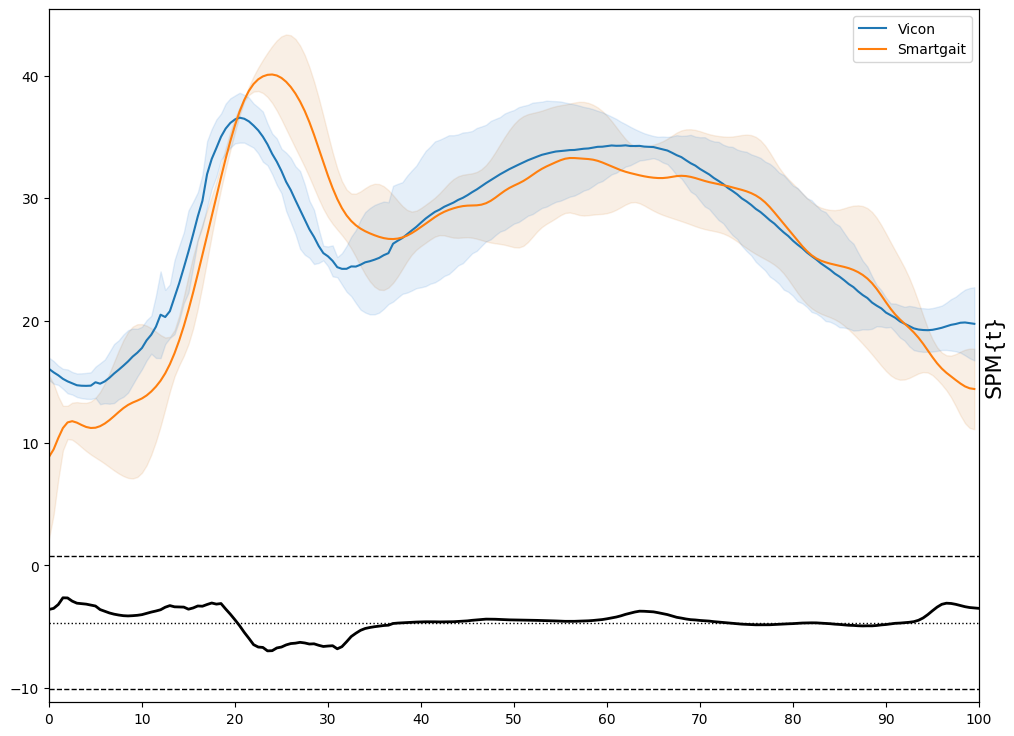

Supplement: Supplementary file 1 [file sensors-24-07819-s001.zip › spm_eval_MA03AL08_sagital/MA03AL08_angle_(2, 5, 12, 0)2.csv_plot_spm.png]

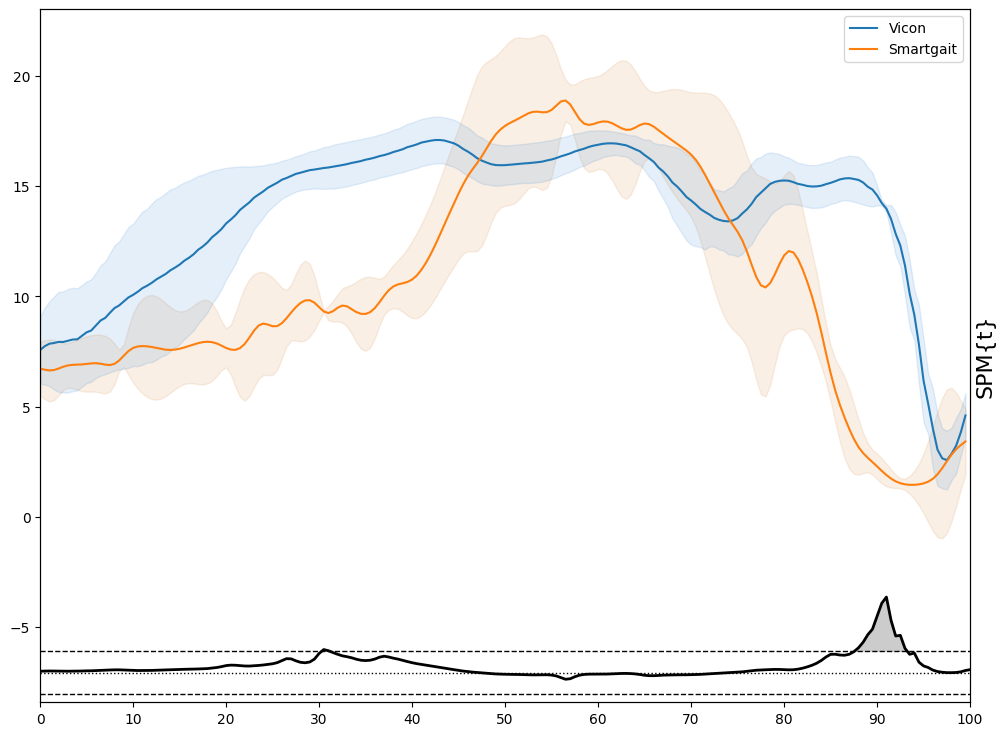

Supplement: Supplementary file 1 [file sensors-24-07819-s001.zip › spm_eval_MA03AL08_sagital/MA03AL08_angle_(5, 8, 8, 11)2.csv_plot_spm.png]

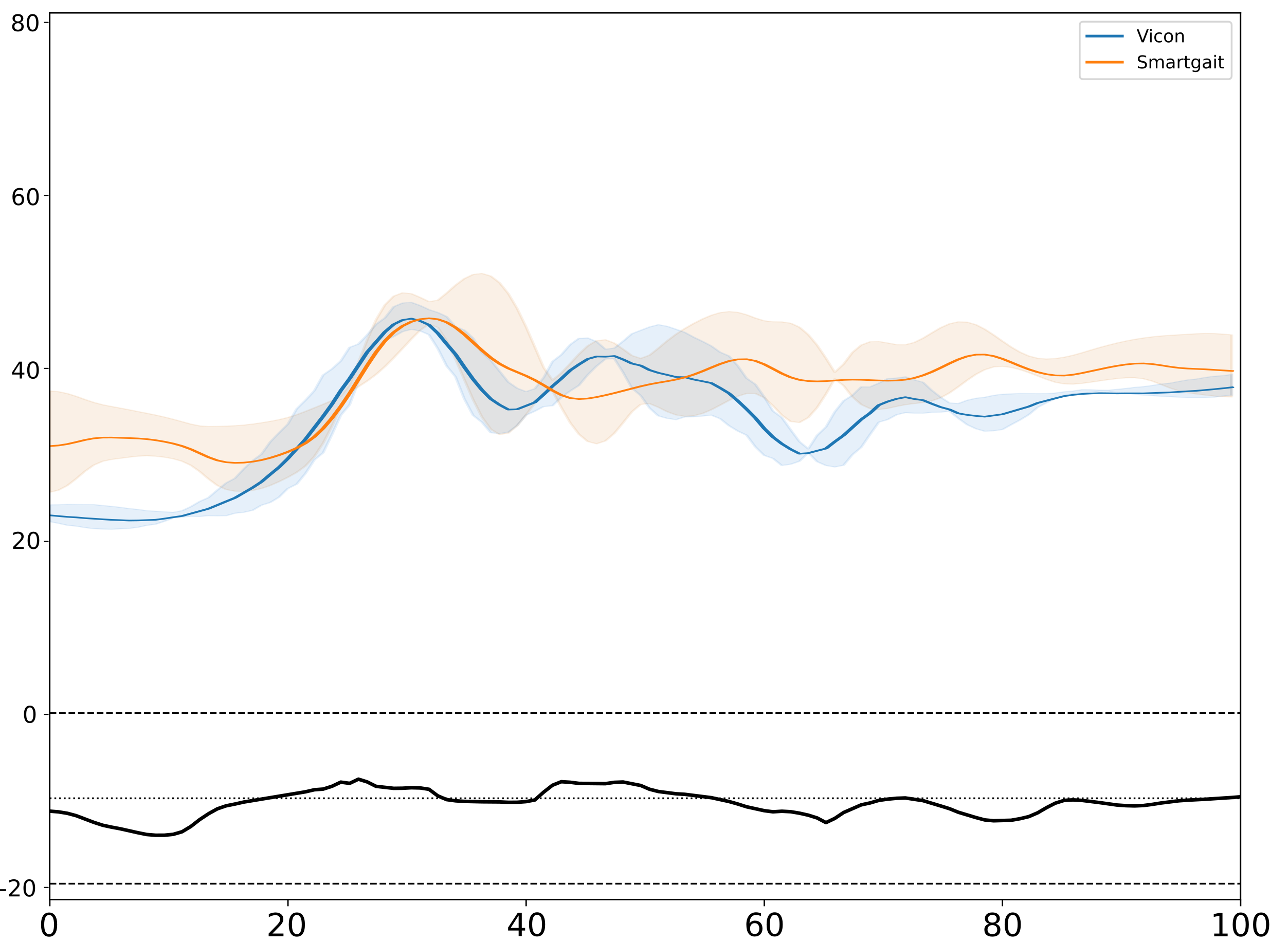

Supplement: Supplementary file 1 [file sensors-24-07819-s001.zip › spm_eval_MA03AL08_sagital/MA03AL08_angle_(2, 5, 5, 8)2.csv_plot_spm_fixed.png]

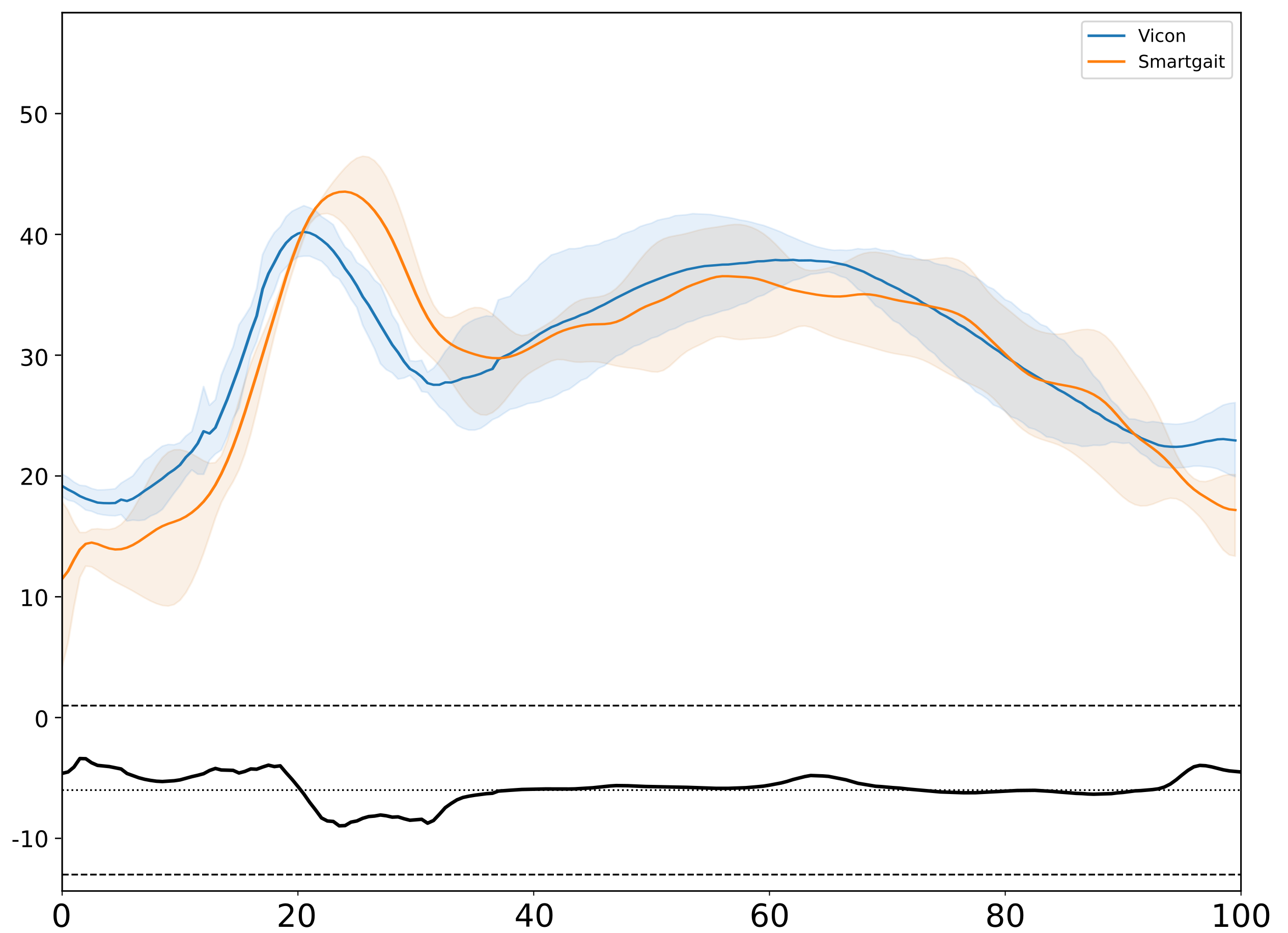

Supplement: Supplementary file 1 [file sensors-24-07819-s001.zip › spm_eval_MA03AL08_sagital/MA03AL08_angle_(2, 5, 12, 0)2.csv_plot_spm_fixed.png]

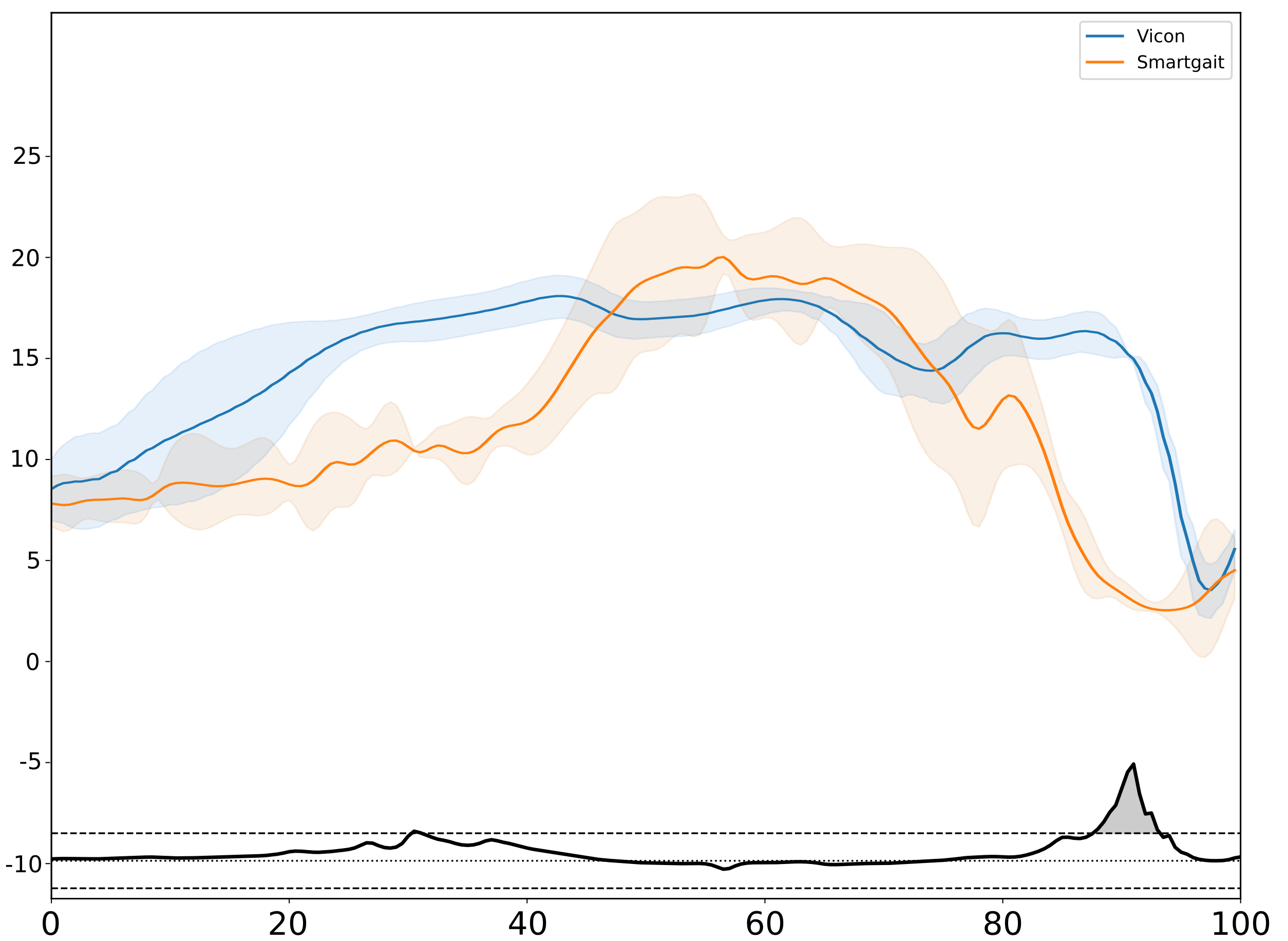

Supplement: Supplementary file 1 [file sensors-24-07819-s001.zip › spm_eval_MA03AL08_sagital/MA03AL08_angle_(5, 8, 8, 11)2.csv_plot_spm_fixed.png]

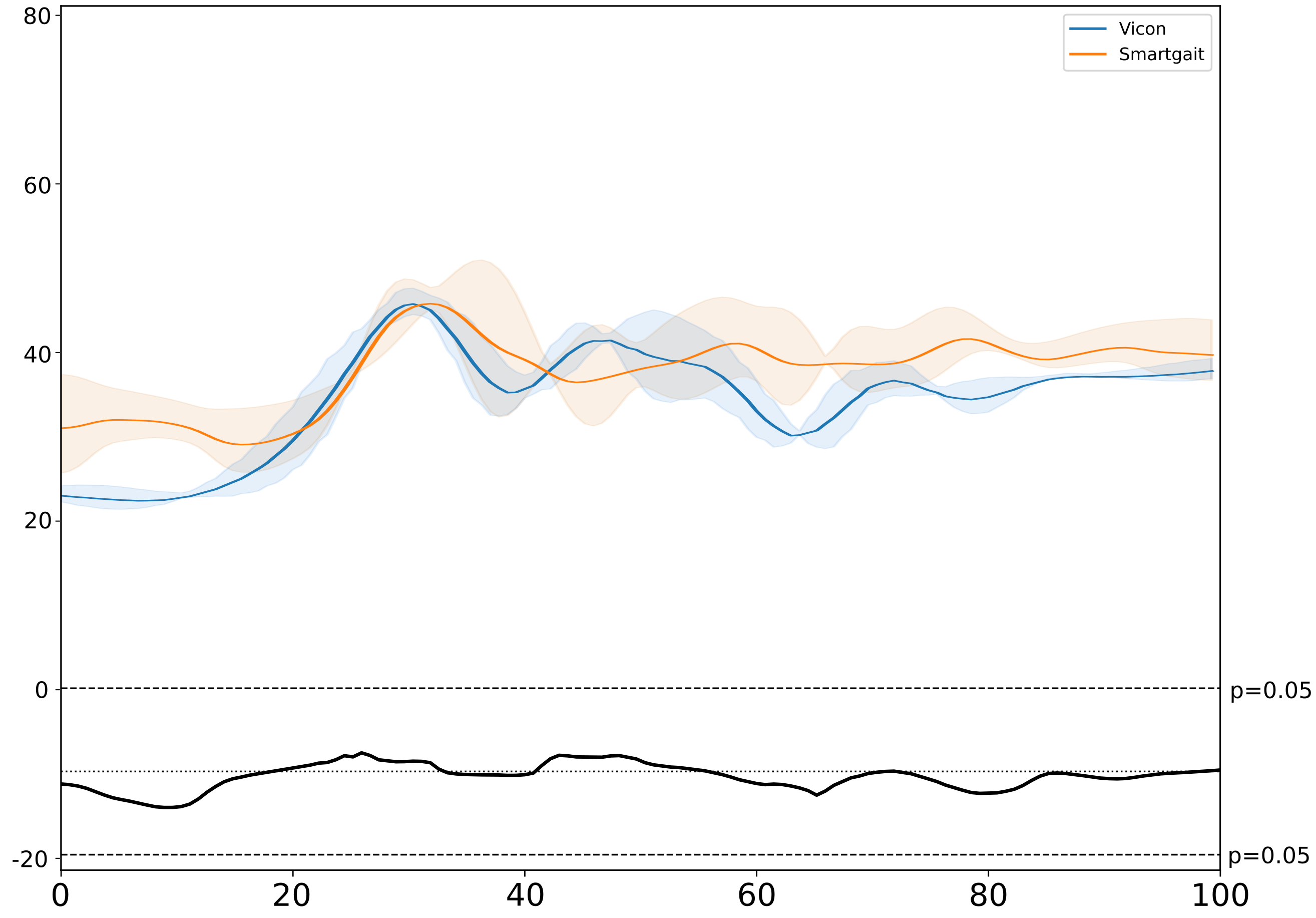

Supplement: Supplementary file 1 [file sensors-24-07819-s001.zip › spm_eval_MA03AL08_sagital/MA03AL08_angle_(2, 5, 5, 8)2.csv_plot_spm_fixed_.png]

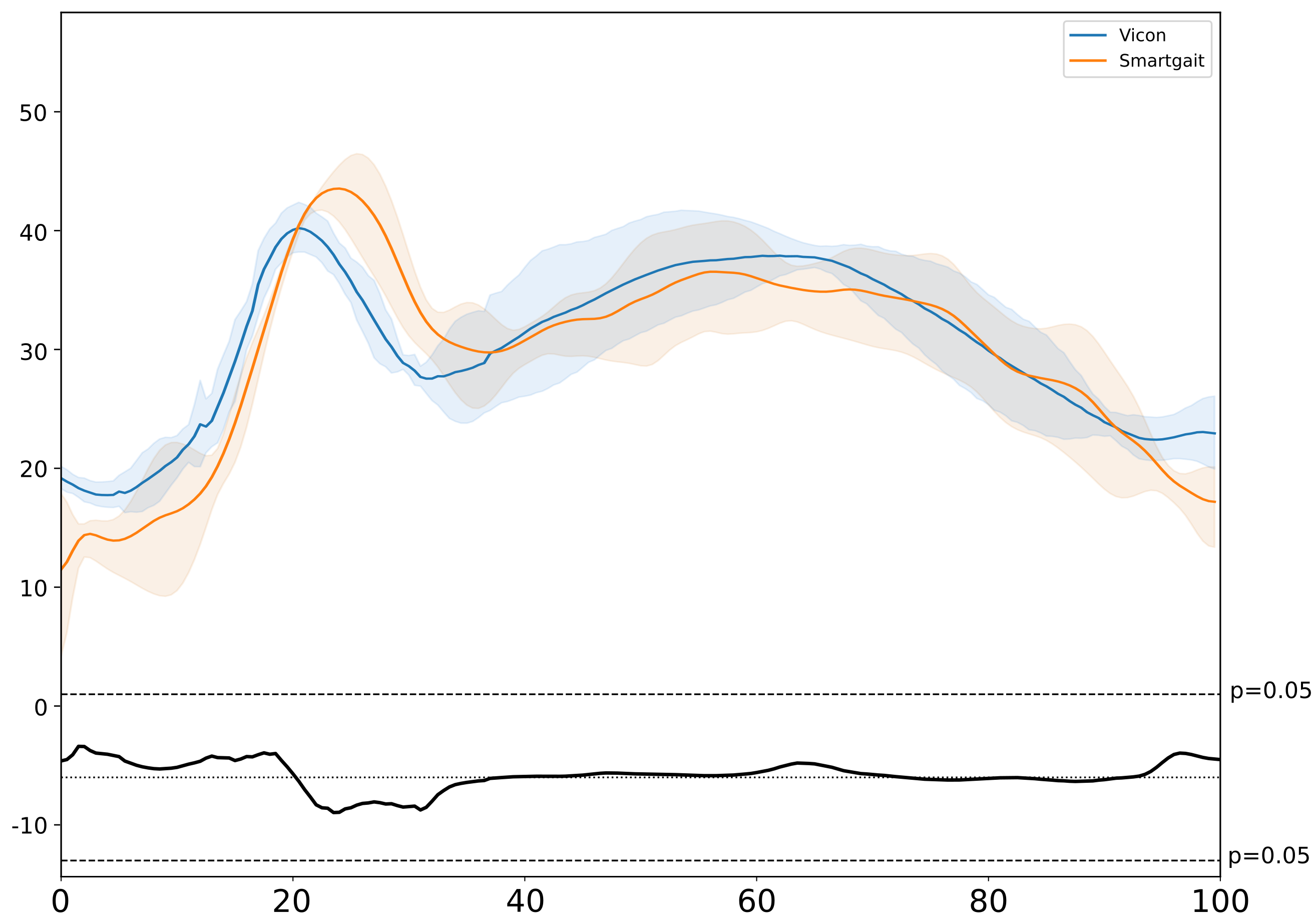

Supplement: Supplementary file 1 [file sensors-24-07819-s001.zip › spm_eval_MA03AL08_sagital/MA03AL08_angle_(2, 5, 12, 0)2.csv_plot_spm_fixed_.png]

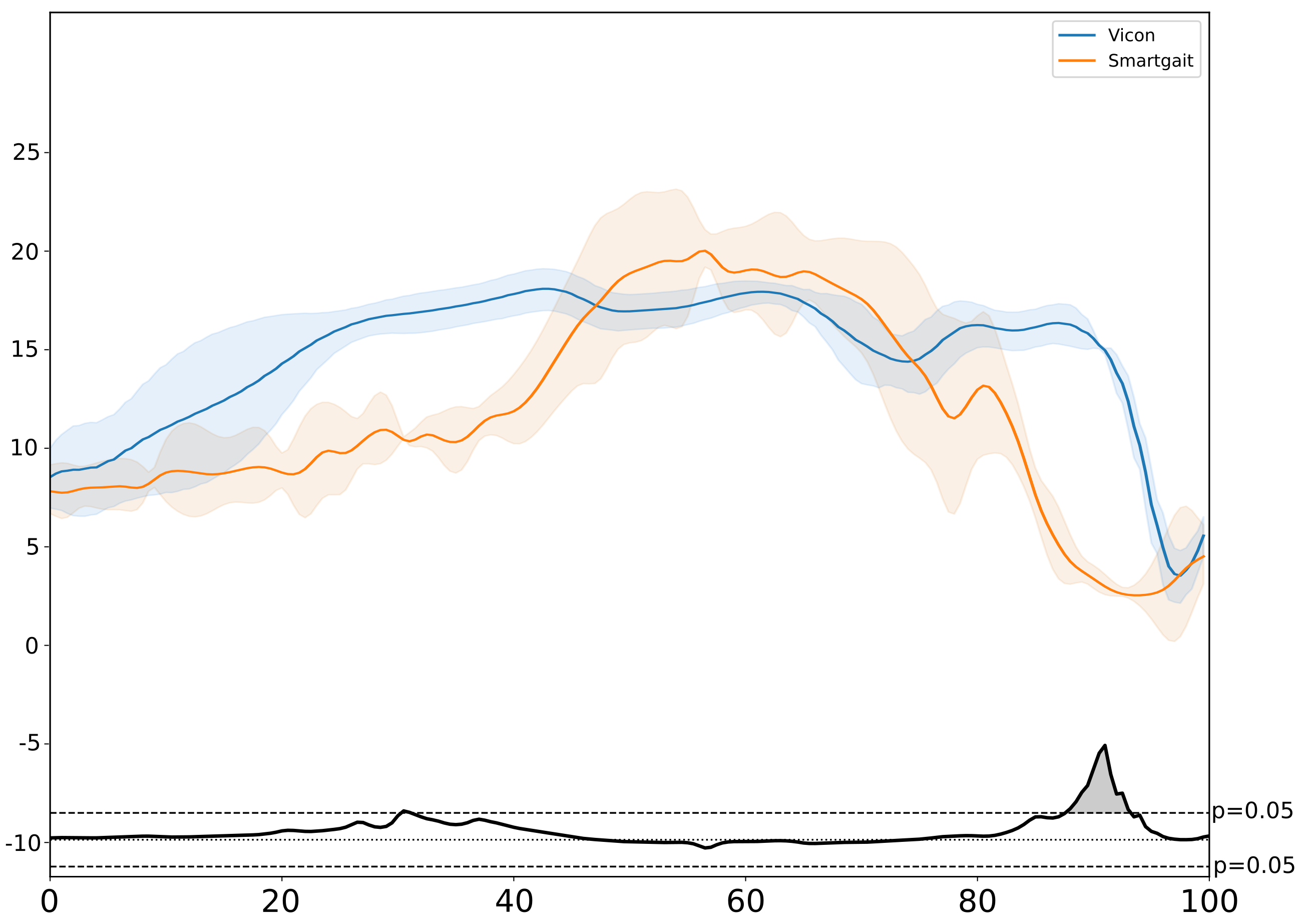

Supplement: Supplementary file 1 [file sensors-24-07819-s001.zip › spm_eval_MA03AL08_sagital/MA03AL08_angle_(5, 8, 8, 11)2.csv_plot_spm_fixed_.png]

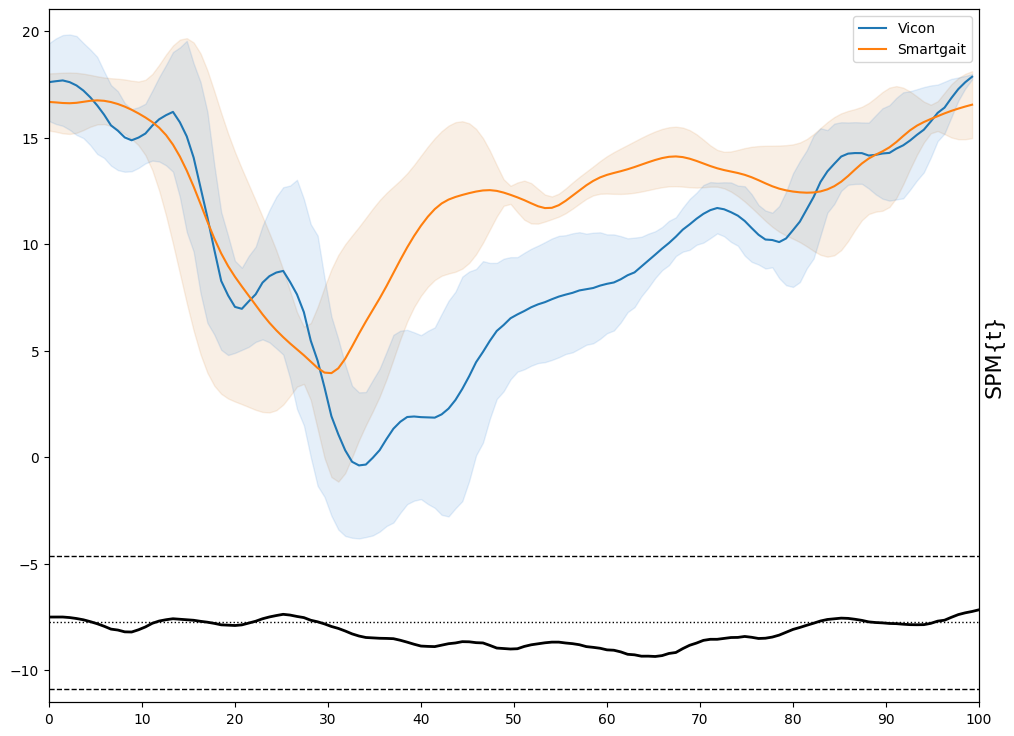

Supplement: Supplementary file 1 [file sensors-24-07819-s001.zip › spm_eval_SE05CH16_frontal/SE05CH16_angle_(2, 5, 12, 0)2.csv_plot_spm.png]

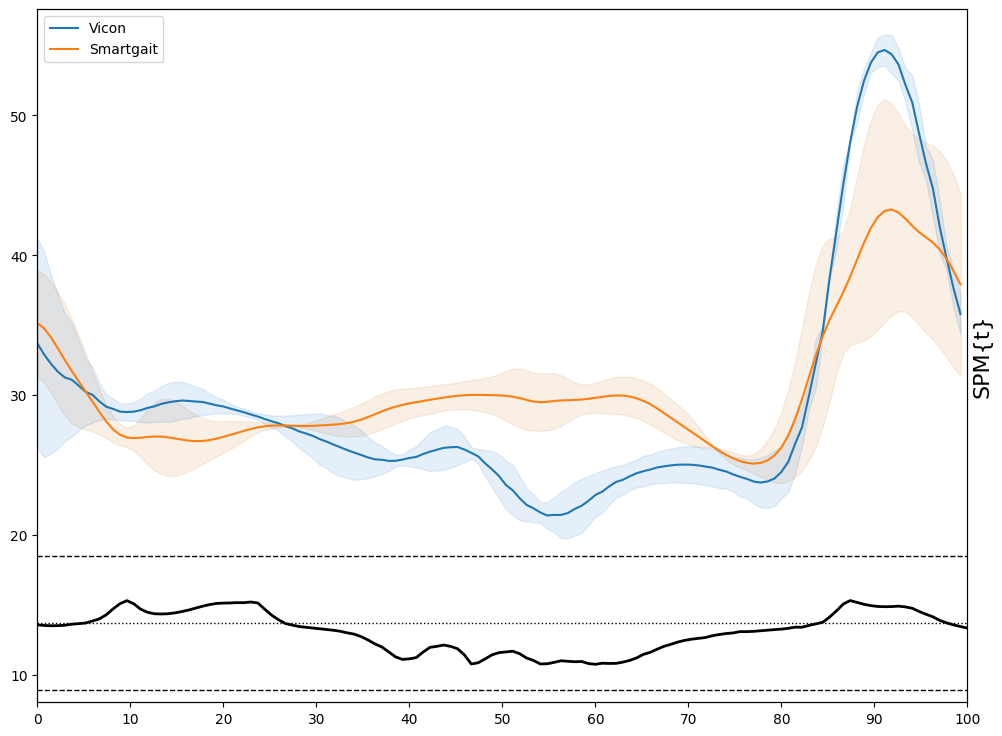

Supplement: Supplementary file 1 [file sensors-24-07819-s001.zip › spm_eval_SE05CH16_frontal/SE05CH16_angle_(2, 5, 5, 8)2.csv_plot_spm.png]

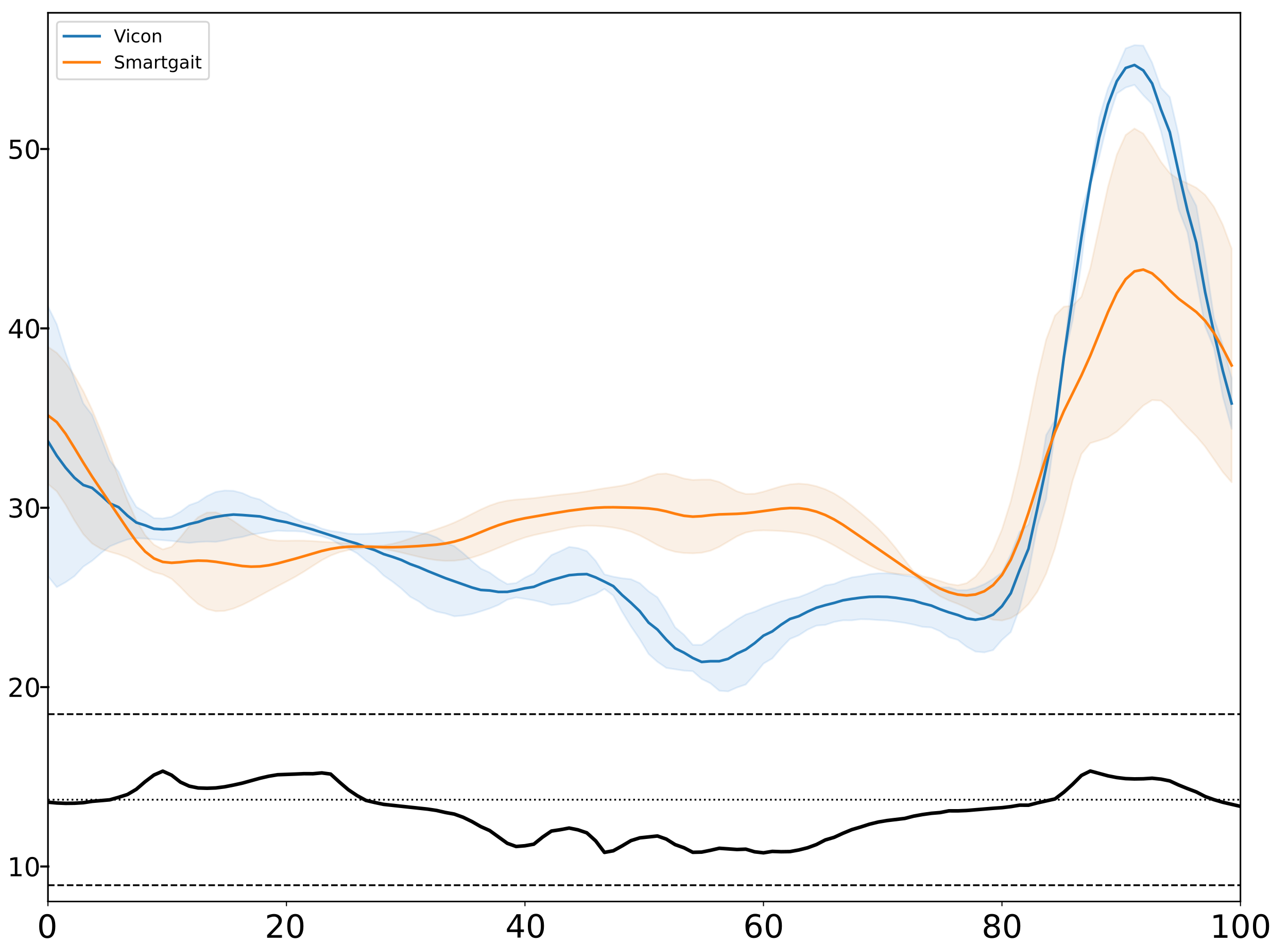

Supplement: Supplementary file 1 [file sensors-24-07819-s001.zip › spm_eval_SE05CH16_frontal/SE05CH16_angle_(2, 5, 5, 8)2.csv_plot_spm_fixed.png]

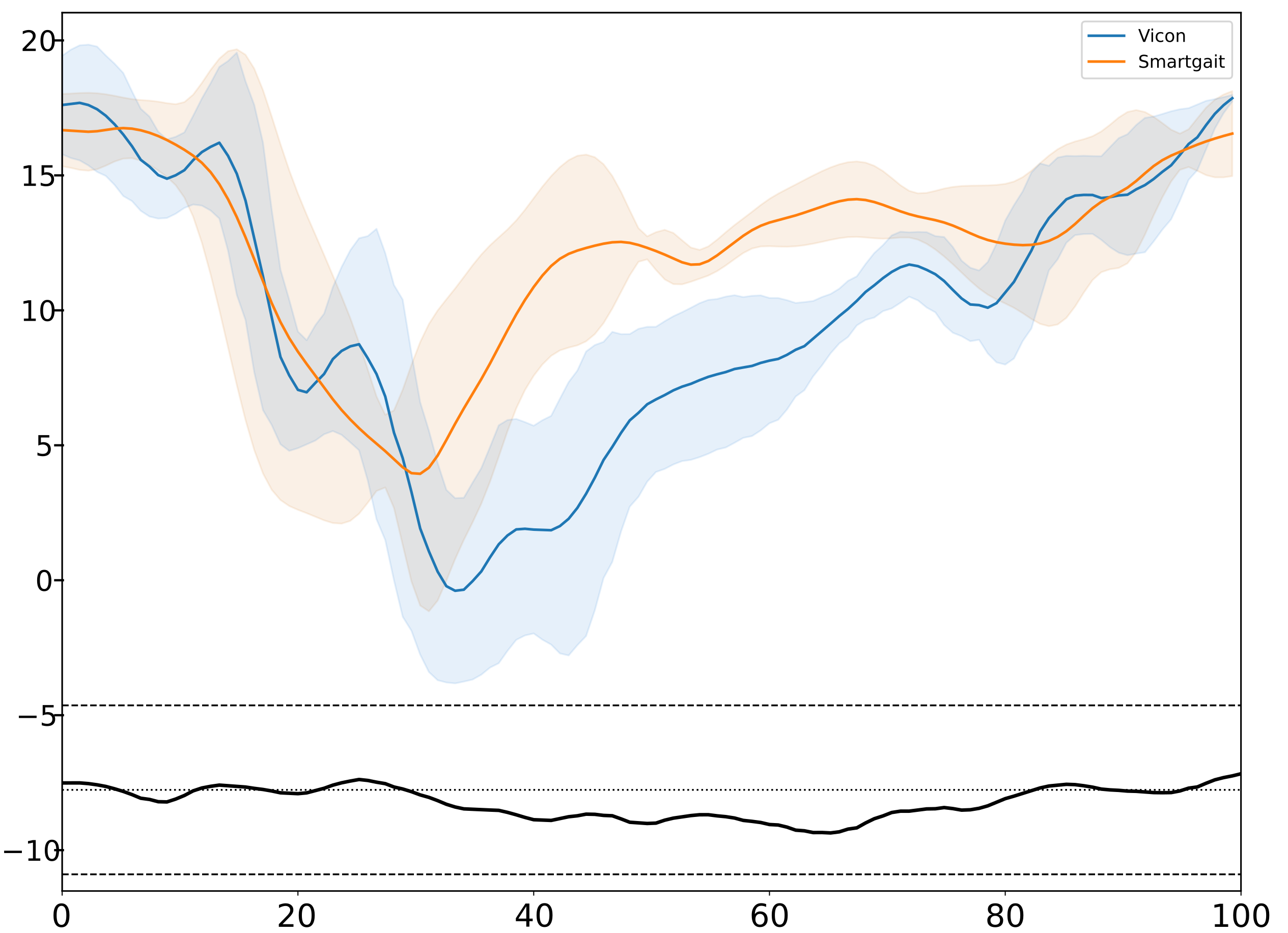

Supplement: Supplementary file 1 [file sensors-24-07819-s001.zip › spm_eval_SE05CH16_frontal/SE05CH16_angle_(2, 5, 12, 0)2.csv_plot_spm_fixed.png]

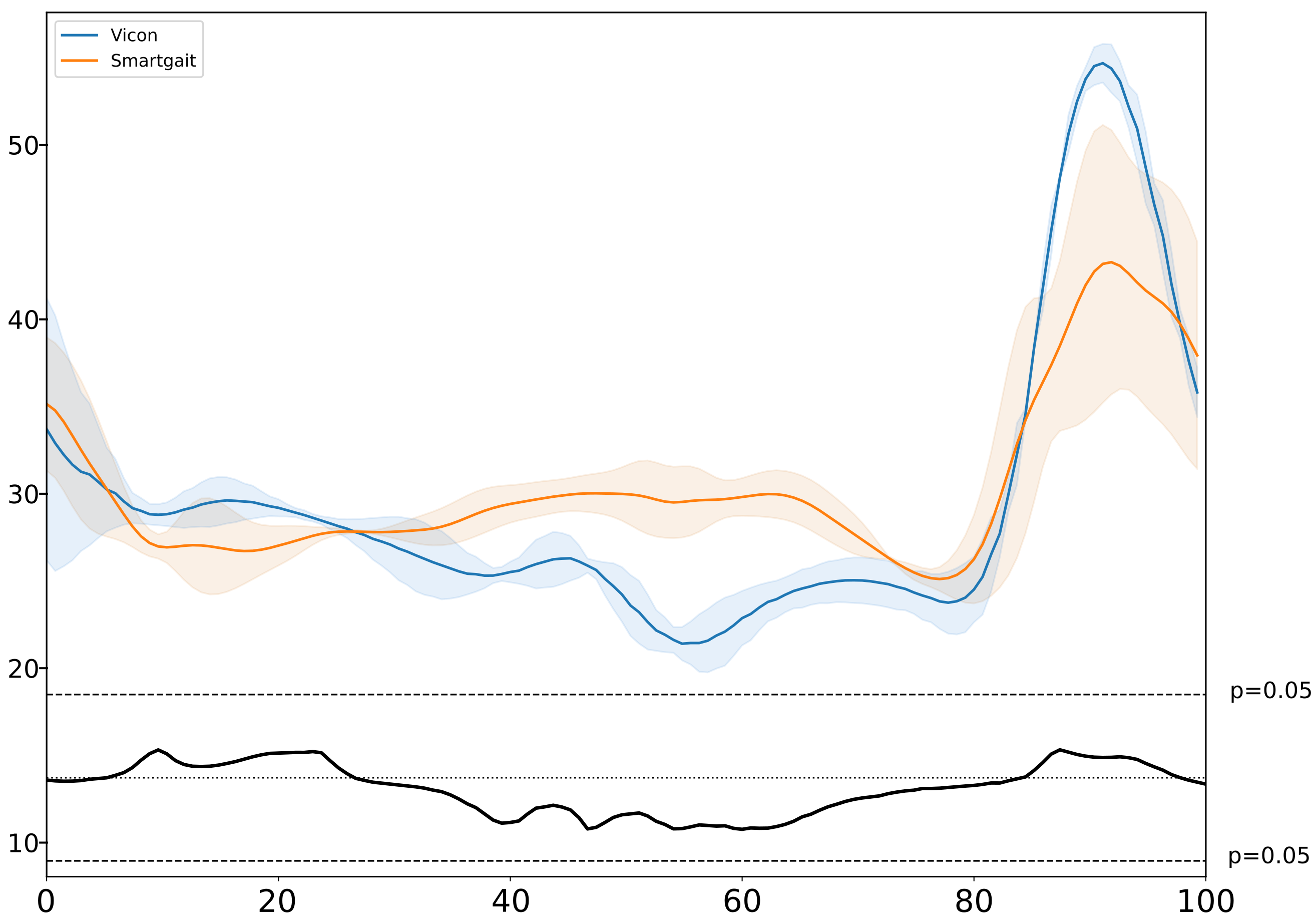

Supplement: Supplementary file 1 [file sensors-24-07819-s001.zip › spm_eval_SE05CH16_frontal/SE05CH16_angle_(2, 5, 5, 8)2.csv_plot_spm_fixed_.png]

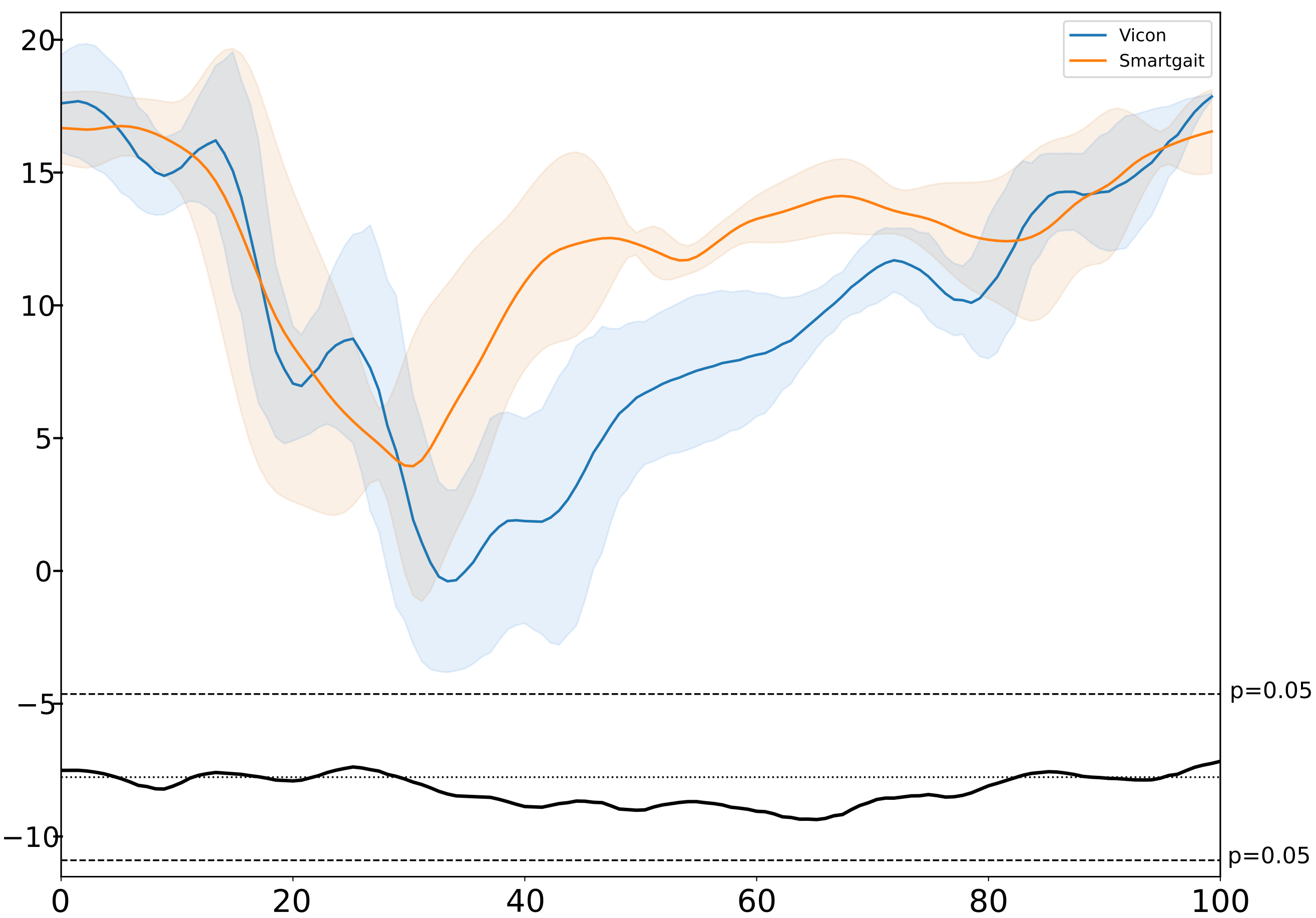

Supplement: Supplementary file 1 [file sensors-24-07819-s001.zip › spm_eval_SE05CH16_frontal/SE05CH16_angle_(2, 5, 12, 0)2.csv_plot_spm_fixed_.png]

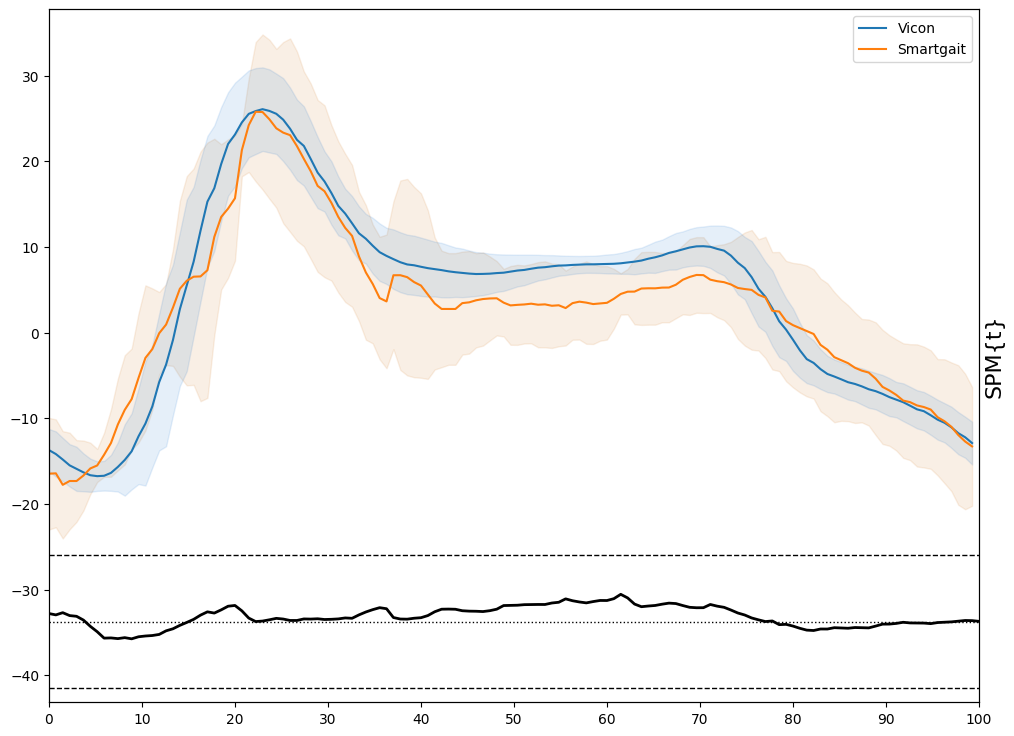

Supplement: Supplementary file 1 [file sensors-24-07819-s001.zip › spm_eval_SE05CH16_sagital/SE05CH16_angle_(2, 5, 12, 0)3.csv_plot_spm.png]

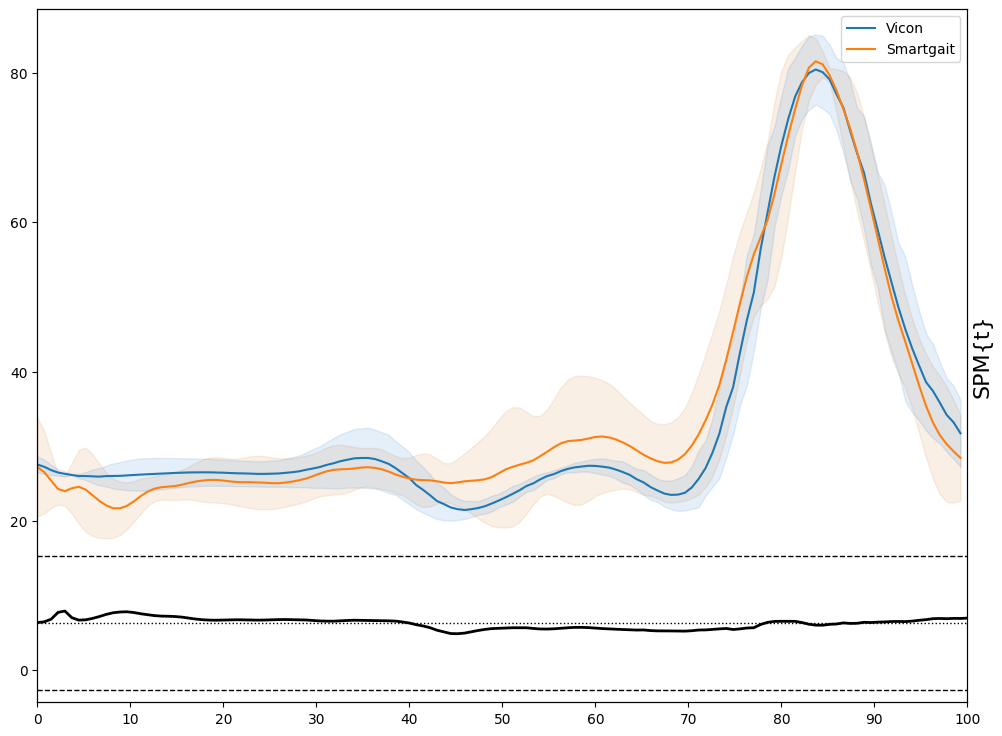

Supplement: Supplementary file 1 [file sensors-24-07819-s001.zip › spm_eval_SE05CH16_sagital/SE05CH16_angle_(2, 5, 5, 8)2.csv_plot_spm.png]

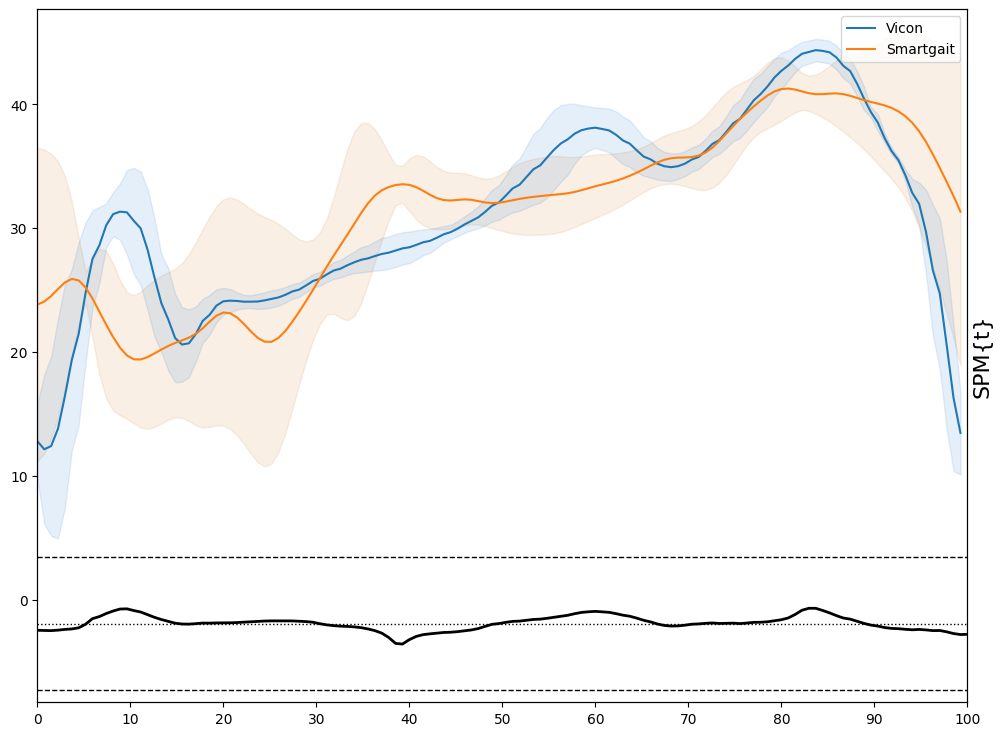

Supplement: Supplementary file 1 [file sensors-24-07819-s001.zip › spm_eval_SE05CH16_sagital/SE05CH16_angle_(5, 8, 8, 11)2.csv_plot_spm.png]
